# Local QSAR based on quantum chemistry calculations for the stability of nitrenium ions to reduce false positive outcomes from standard QSAR systems for the mutagenicity of primary aromatic amines

**DOI:** 10.1186/s41021-024-00318-4

**Published:** 2024-11-21

**Authors:** Shigeharu Muto, Ayako Furuhama, Mika Yamamoto, Yasuteru Otagiri, Naoki Koyama, Seiji Hitaoka, Yusuke Nagato, Hirofumi Ouchi, Masahiro Ogawa, Kisako Shikano, Katsuya Yamada, Satoshi Ono, Minami Hoki, Fumiya Ishizuka, Soichiro Hagio, Chiaki Takeshita, Hisayoshi Omori, Kiyohiro Hashimoto, Satsuki Chikura, Masamitsu Honma, Kei-ichi Sugiyama, Masayuki Mishima

**Affiliations:** 1 Translational Research Division, Chugai Pharmaceutical Co., Ltd., 216-Banchi Totsuka-Cho, Totsuka-Ku, Yokohama, Kanagawa 244-8602 Japan; 2 Division of Genome Safety Science, National Institute of Health Sciences, 3–25–26, Tonomachi, Kawasaki-Ku, Kawasaki, Kanagawa 210–9501 Japan; 3 Non-Clinical Biomedical Science, Applied Research & Operations, Astellas Pharma Inc., 21, Miyukigaoka, Tsukuba-Shi, Ibaraki, 305-8585 Japan; 4 Human Translational Research Group, EA Pharma Co., Ltd., 2-1-1 Irifune, Chuo-Ku, Tokyo, 104-0042 Japan; 5 Eisai Co., Ltd., 5-1-3 Tokodai, Tsukuba-Shi, Ibaraki, 300-2635 Japan; 6 Toyama Research and Development Center, FUJIFILM Toyama Chemical Co., Ltd., 4-1, Shimookui 2-Chome, Toyama, 930-8508 Japan; 7 Toxicology Research Lab., Central Pharmaceutical Research Institute, Japan Tobacco Inc., 1-13-2 Fukuura, Kanazawa-Ku, Yokohama, Kanagawa 236-0004 Japan; 8 Life Science Research Institute, Kumiai Chemical Industry Co., Ltd., 276 Tamari, Kakegawa, Shizuoka, 436-0011 Japan; 9 Safety Research Laboratories, Sohyaku. Innovative Research Division, Mitsubishi Tanabe Pharma Corporation, 2-26-1, Muraoka-Higashi, Fujisawa, Kanagawa 251-8555 Japan; 10 Discovery Technology Laboratories, Innovative Research Division, Mitsubishi Tanabe Pharma Corporation, 1000, Kamoshida-Cho, Aoba-Ku, Yokohama, 227-0033 Japan; 11 Research Division, Nihon Nohyaku Co., Ltd., 345, Oyamada-Cho, Kawachinagano-Shi, Osaka, 586-0094 Japan; 12 Safety Assessment Department, Nippon Shinyaku Co., Ltd., 14, Nishinosho-Monguchi-Cho, Kisshoin, Minami-Ku, Kyoto, 601-8550 Japan; 13 Biological Research Laboratories, Nissan Chemical Corporation, 1470 Shiraoka, Shiraoka-Shi, Saitama, 349-0294 Japan; 14 Safety Research Laboratories, Ono Pharmaceutical Co., Ltd, 3-1-1, Sakurai, Shimamoto-Cho, Mishima-Gun, Osaka, 618-8585 Japan; 15 Preclinical Basic Research, Discovery and Preclinical Research Division, Taiho Pharmaceutical Co., Ltd., 3, Okubo, Tsukuba, Ibaraki, 300-2611 Japan; 16 Drug Safety Research and Evaluation, Research, Takeda Pharmaceutical Company Limited, Kanagawa, 251-8555 Japan; 17 Teijin Pharma Limited, 4-3-2 Asahigaoka, Hino, Tokyo 191-8512 Japan

**Keywords:** Primary aromatic amine, QSAR, Mutagenicity, Nitrenium ion, Stability, Quantum chemistry, False positive, ICH M7

## Abstract

**Background:**

Primary aromatic amines (PAAs) present significant challenges in the prediction of mutagenicity using current standard quantitative structure activity relationship (QSAR) systems, which are knowledge-based and statistics-based, because of their low positive prediction values (PPVs). Previous studies have suggested that PAAs are metabolized into genotoxic nitrenium ions. Moreover, ddE, a relative-energy based index derived from quantum chemistry calculations that measures the stability nitrenium ions, has been correlated with mutagenicity. This study aims to further examine the ability of the ddE-based approach in improving QSAR mutagenicity predictions for PAAs and to develop a refined method to decrease false positive predictions.

**Results:**

Information on 1,177 PAAs was collected, of which 420 were from public databases and 757 were from in-house databases across 16 laboratories. The total dataset included 465 Ames test-positive and 712 test-negative chemicals. For internal PAAs, detailed Ames test data were scrutinized and final decisions were made using common evaluation criteria. In this study, ddE calculations were performed using a convenient and consistent protocol. An optimal ddE cutoff value of -5 kcal/mol, combined with a molecular weight ≤ 500 and ortho substitution groups yielded well-balanced prediction scores: sensitivity of 72.0%, specificity of 75.9%, PPV of 65.6%, negative predictive value of 80.9% and a balanced accuracy of 74.0%. The PPV of the ddE-based approach was greatly reduced by the presence of two ortho substituent groups of ethyl or larger, as because almost all of them were negative in the Ames test regardless of their ddE values, probably due to steric hindrance affecting interactions between the PAA and metabolic enzymes. The great majority of the PAAs whose molecular weights were greater than 500 were also negative in Ames test, despite ddE predictions indicating positive mutagenicity.

**Conclusions:**

This study proposes a refined approach to enhance the accuracy of QSAR mutagenicity predictions for PAAs by minimizing false positives. This integrative approach incorporating molecular weight, ortho substitution patterns, and ddE values, substantially can provide a more reliable basis for evaluating the genotoxic potential of PAAs.

**Supplementary Information:**

The online version contains supplementary material available at 10.1186/s41021-024-00318-4.

## Introduction

Primary aromatic amines (PAAs) are important materials, reagents, or intermediates involved in the synthesis of various chemical products and are key substructures in many chemicals used in daily life. Because some PAAs are genotoxic carcinogens [1], it is critical to conduct risk assessments for PAA-containing products that come in contact with humans. Notably, the migration of PAAs from food packaging materials and kitchenware has been identified as a major source of human exposure [2–6]. Regulations set by the European Union mandates that the release of PAAs in food should not exceed 2 μg/kg, which corresponds to the practical detection limit [7]. Additionally, pharmaceutical impurities are another potential source of human PAA exposure. The International Council for Harmonization of Technical Requirements for Pharmaceuticals for Human Use (ICH) established the M7 guideline, which calls for a quantitative structure–activity relationship (QSAR) assessment of DNA reactive (mutagenic) impurities in pharmaceuticals to minimize potential carcinogenic risk [8] using knowledge-based and statistics-based systems. Because numerous products contain various types of PAAs, applying QSAR predictions to safety assessment is a realistic solution. The adoption of QSAR systems has been expanding in chemical regulatory environments outside of the pharmaceutical industry [9–11].

Despite great efforts [12–20] to improve QSAR predictions, PAAs remain a significant challenge for QSAR mutagenicity assessment. Widely used QSAR systems overestimate the mutagenicity of PAAs [21, 22]. Approximately half of PAAs identified as mutagenic by QSAR systems were empirically non-mutagenic in the Ames test, which is supported by a survey conducted by Patel et al. [22]. They examined PAAs from a group of pharmaceutical companies with three commonly-used QSAR tools. The positive prediction value (PPV) / negative prediction value (NPV) for each of the three QSAR tool was 36/81%, 49/83%, and 61/84%. This demonstrates that standard QSAR assessments result in considerably frequent false positive calls. Like false negative predictions, false positive predictions can also misrepresent actual risk and, further, lead to practical drawbacks. These include delays in the launch of new drugs and increased purification costs, which ultimately raises drug prices and altogether disadvantages the patients. Improving the PPV of QSAR predictions is therefore not only a scientific priority but also a necessity for improving the cost-efficiency and speed of drug development.

Great efforts to predict mutagenicity and carcinogenicity based on chemical structure have been ongoing since before the release of ICH M7 (reviewed in [23]). Various parameters, such as classical physicochemical properties, topological and geometric indices, and quantum chemical descriptors, have all been reported as useful. Bentzien et al. [24] proposed a method for predicting the mutagenicity of aromatic amines based on the stability (ddE values) of nitrenium ions derived from quantum mechanics calculations. This approach is grounded in the nitrenium ion hypothesis [25], which posits that the stability of a transient nitrenium ions—enzymatically converted from an aromatic amines—is correlated with mutagenicity. Their examination of 257 PAAs indicated that this method could provide complementary information to enhance the predictivity of widely used QSAR tools like Derek Nexus (Lhasa ltd.), CASE Ultra (MultiCASE inc.) and Leadscope (Insteam). Furukawa et al. [26] recently applied Bentzien’s method to 85 in-house PAAs, conveniently utilizing MOE software for ddE calculations. Their findings showed a good correlation between ddE values and Ames test results, further validating the efficacy of this approach.

The aim of our study was to further evaluate the feasibility of employing ddE-based local QSAR models for PAAs and to propose a refined approach to support the expert reviews of the outcomes of the current standard QSAR systems. In this study, we collected information on 1,177 PAAs, reviewed the detailed Ames test results, and utilized Furukawa’s method to calculate ddE. We then compared these ddE values with the Ames test results and devised an approach for rectifying false positive predictions within existing QSAR flameworks.

## Materials and methods

### Ames test data collection for primary aromatic amines

Ames test data for PAA (Table 1) were collected from public databases, 15 pharmaceutical and chemical companies, and the National Institute of Health Sciences (NIHS). The Ames test data for in-house compounds had not been disclosed publicly. Because the detailed chemical structures for the many of the collected PAAs (mainly in-house compounds) were not available, data integrity was verified using the following acceptance criteria. 1) Data were obtained using the standard Ames test method. Data from modified assays like mini-Ames tests or fluctuation Ames assays were excluded. 2) Ames tests were performed using at least two tester strains, *Salmonella typhimurium* TA98 and TA100, both in the presence and absence of metabolic activation systems and within reasonable concentration ranges. 3) Duplication was eliminated by checking molecular weights and other chemical properties. 4) PAAs with additional structure alerts from common QSAR systems, beyond the aromatic amine alert, were excluded.

**Table 1:** Dataset of primary aromatic amines in this study

	Information source	Ames test result		Total
		Positive	Negative	
Total dataset^a^		465	712	1177
	Public database	249	171	420
	In-house	216	541	757
Working dataset^b^		322	510	832

^a^Sum of public and internal compounds with reliable Ames test data

^b^Excluding the compounds with non-aromatic amine alerts, MW > 500, ring-opening remarks, two or more amine groups

All Ames test data were meticulously reviewed by several authors to determine mutagenic activity using common evaluation criteria. The review process led to some revisions of the original Ames test conclusions; for example, some results were changed from negative to positive due to weak but reproducible dose-dependent increases in the number of revertant colonies. The total dataset included 1,177 compounds that had reliable Ames test results. The working dataset composed of 832 compounds was prepared by excluding compounds that had additional structure alerts other than aromatic amines, molecular weights greater than 500, ring-opening remarks from ddE calculations, or two or more amine groups (Table 1).

### ddE calculation for the stability of nitrenium ions

The ddE, an indicator of nitrenium ion stability, was calculated by running a Scientific Vector Language (SVL) script for the Molecular Operating Environment (MOE) named "mut_nitre.svl" provided by MOLSIS Inc., in accordance with a previously described method, using MOE 2019.01 software (Chemical Computing Group ULC, Canada) [26]. We utilized the heat of formation energies of AM1-optimized structures calculated with MOPAC v7.1 in MOE 2019.01. To compute ddE, we created a 3D structure of the molecule using MOE, washed the structure, performed conformational sampling with LowModeMD using MMFF94x force field, and optimized geometry using AM1 Hamiltonian. The most stable conformer was used to determine the nitrenium ion species by replacing an amine hydrogen with a dummy atom X and re-optimizing the geometry with CHARGE = + 1. The lowest ddE value was recorded. Aniline’s ddE was set to 0 kcal/mol. If geometry optimization failed, a NaN (Not a Number) was assigned.

### Analysis of prediction performance with ddE values

The prediction performance of various ddE cutoff values were assessed using several metrics: accuracy, sensitivity, specificity, PPV, NPV, the Matthews correlation coefficient (MCC), and coverage, as previously described [27]. A receiver operating characteristic (ROC) curve was drawn using the roc_curve function in the sklearn package in Python 3.10.12 on Google Collaboratory, and Youden’s index was calculated using Microsoft Excel 2021 to determine the optimal cutoff value [28].

The positive and negative likelihood ratio (LR + and LR-) of the ddE approach were calculated as previously described [29].

## Results

### Prediction performances on the total dataset

The results of the prediction performance analysis of the total dataset at various ddE cutoff values are shown in Table 2 (details are provided in the Supplementary data). The cutoff values increased stepwise from -10 kcal/mol to + 10 kcal/mol in increments of 2.5 kcal/mol. The balanced accuracy increased to 71.6% at ddE = -5 kcal/mol when the MCC was 0.35. A cutoff value of ddE = 0 kcal/mol gave the best MCC of 0.38 and a balanced accuracy of 69.8%.

**Table 2:** Prediction performance with various ddE threshold for total dataset

ddE cut-off value (kcal/moll)	10	7.5	5	2.5	0	-2.5	-5	-7.5	-10
Sensitivity (%)	93.0	90.6	88.2	85.9	83.7	79.1	74.1	64.5	57.6
Specificity (%)	30.9	36.7	43.0	49.4	55.9	62.6	69.1	72.6	76.5
Positive prediction value (%)	48.1	49.7	51.6	53.9	56.7	59.4	62.3	61.8	62.8
Negative prediction value (%)	86.6	85.1	84.1	83.5	83.3	81.3	79.5	74.8	72.3
Accuracy (%)	56.3	58.7	61.4	64.3	67.2	69.4	71.1	69.3	68.8
Balanced accuracy (%)	62.0	63.7	65.6	67.6	69.8	70.9	71.6	68.5	67.0
Mathews correlation coefficient	0.34	0.35	0.36	0.37	0.38	0.37	0.35	0.29	0.26
Positive likelihood ratio	1.35	1.43	1.55	1.70	1.90	2.11	2.40	2.35	2.45
Negative likelihood ratio	0.23	0.26	0.27	0.29	0.29	0.33	0.37	0.49	0.55
Coverage^a^ (%)	86.8								

^a^Percentage of ddE computable PAAs, of which 40.8% Ames positive

### Ring opening remarks in ddE calculations

**Fig. 1:**
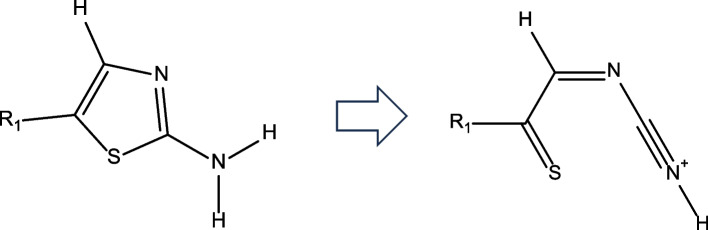
Example of ring-opening compound. Nitrenium ion becomes non-aromatic form

Ring-opening remarks appeared in the ddE calculations for 53 compounds, two of which had structural alerts other than aromatic amines using the common QSAR system. An example of ring opening was shown in Fig. 1. When applying the -5 kcal/mol ddE cutoff to the 51 compounds with only aromatic amine alerts, the number of true positives was 5/40, the number of false positives was 35/40, the number of true negatives was 10/11, and the number of false negatives was 1/11 (Table 3).

**Table 3:** Ames test results and ddE of compounds with ring opening remarks

	ddE value	Ames test		Total
		Positive	Negative	
ddE value	≤ -5 kcal/mol	5	35	40
	> -5 kcal/mol	1	10	11
Total		6	45	51

Prediction performance of ddE values for compounds larger and smaller than a molecular weight (MW) of 500.

The ddE-based prediction in this study very frequently generated false positive calls for large MW PAAs. Of the 68 compounds with MWs > 500, 40 were predicted to be positive because their ddE values were less than -5 kcal/mol, whereas 36/40 of them were negative in the Ames test. Table 4 lists the 40 compounds with ddE < -5 kcal/mol and MWs > 500. Table 5 shows a comparison of the prediction results for ddE between PAAs with MWs > 500 and those with MWs ≤ 500 using a cutoff value of -5 kcal/mol ddE. For the MWs > 500 PAA, the ddE approach resulted in very poor PPV of only 10%. For the MWs ≤ 500 PAA, the PPV was 66.9%. The ddE approach led to many false positive predictions for large PAAs with MWs > 500.

**Table 4:** Comparison of prediction performance of ddE approach between MW > 500 and ≤ 500

MW	Ames	ddE (cutoff -5 kcal/mol)		Sum	
		+	-		
> 500	+	4	4	8	50.0% sensitivity
	-	36	24	60	40.0% specificity
		10.0%^a^	85.7%^b^		41.2% accuracy
≤ 500	+	305	104	409	74.6% sensitivity
	-	151	394	545	72.3% specificity
		66.9%^a^	79.1%^b^		73.3% accuracy

^a^Positive prediction value

^b^Negative prediction value

**Table 5:** False positive rate of ddE based decision on PAAs with ortho substitution

	Example	False positive rate^a^
Class 1: two CH2CH3 or larger substituents at both R1 and R2		88.9% (8/9)
Class 2: one CH2CH3 or larger substituent at ortho position with and without the other ortho substitution		55.0% (44/80)
Class 3: Smaller substituents at R1 and/or R2		17.9% (5/28)
Class 4: No ortho substitution		29% (29/100)

^a^-5 kcal/mol ddE cutoff

### Influence of ortho-position substitution

As described in Table 5, the PAA with ddE values of less than -5 kcal/mol were classified based on their ortho-position substituents into four categories: Class 1 includes PAA with CH_2_CH_3_ or larger substituents at both ortho positions; Class 2 includes PAAs with CH_2_CH_3_ or a larger substituent at one ortho position with and without a smaller substituent at the other ortho position; Class 3 includes PAAs with one or two ortho substituents smaller than CH_2_CH_3_; and Class 4 includes PAAs with no ortho substituents. Positive calls from the ddE approach with a -5 kcal/mol cutoff value resulted in 88.9% false positives for Class 1, 55.0% for Class 2, 17.9% for Class 3, and 29% for Class 4 (Table 5). These results indicate that large substitutions at both ortho positions frequently caused false positives in ddE-based predictions.

### ROC analysis with the working dataset

**Fig. 2:**
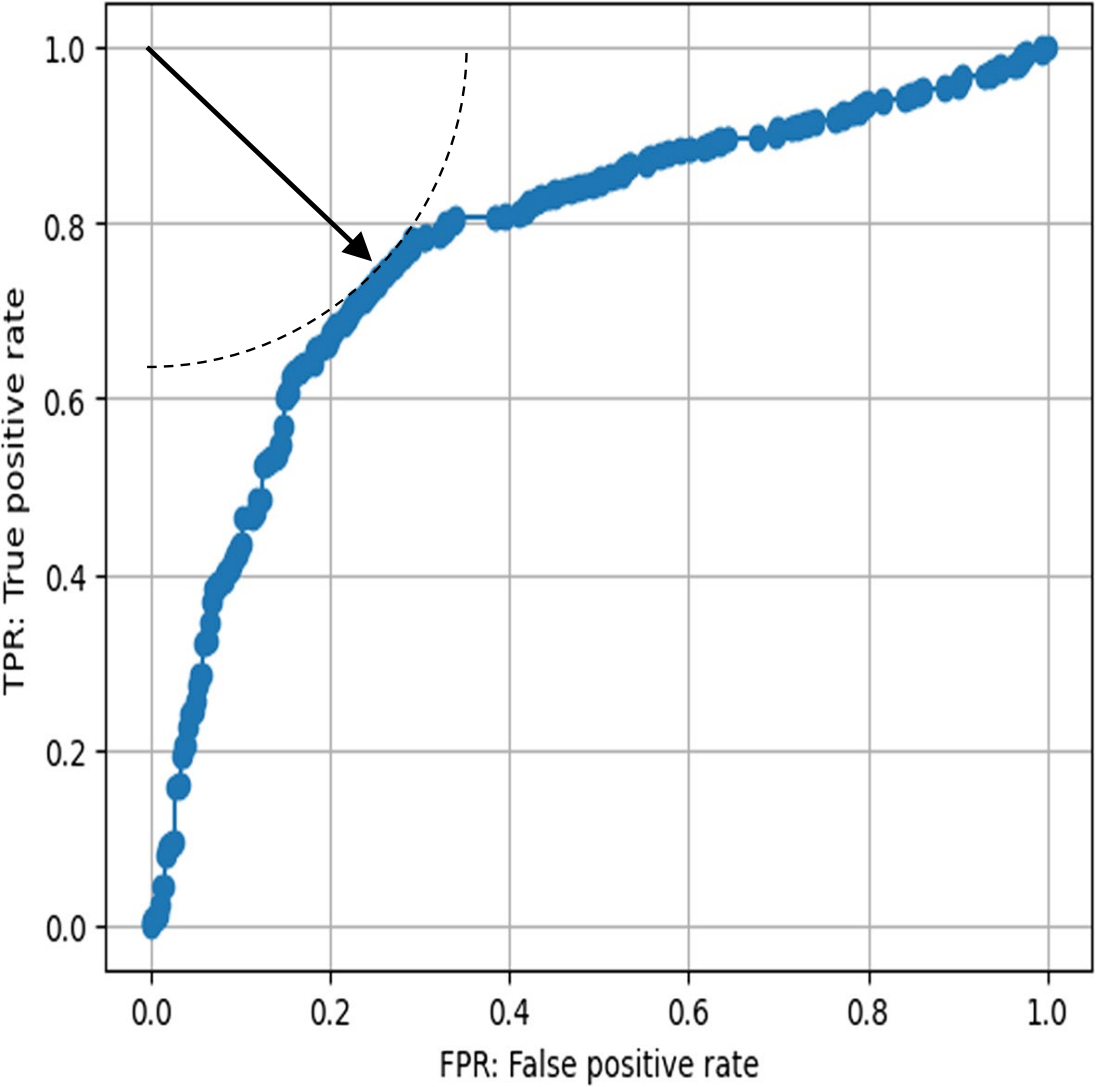
ROC curve of prediction with various cutoff values of ddE of working dataset. Distance between the left upper corner and each plot on the ROC curve were shown in Fig. 2

**Fig. 3:**
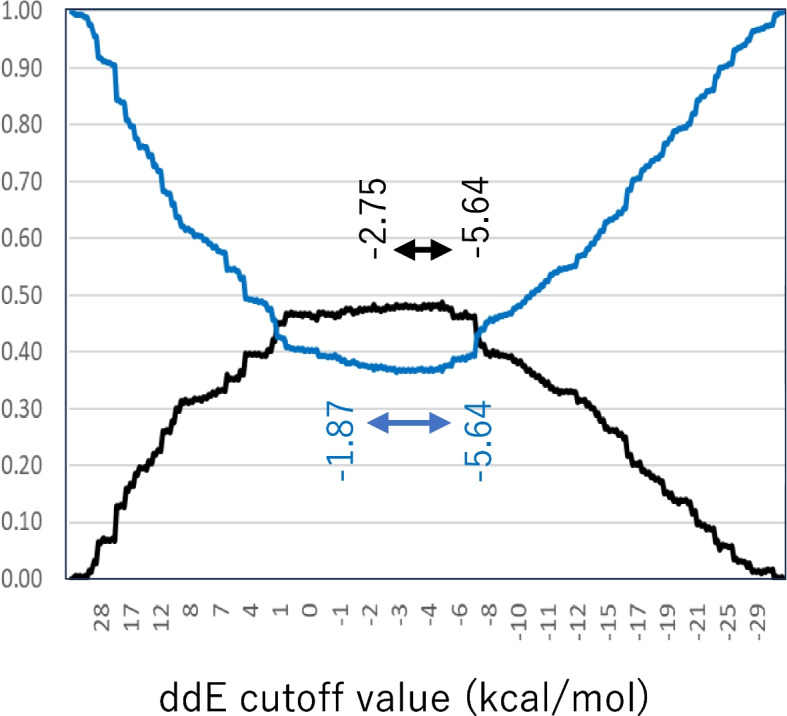
Indicators for optimal cutoff value of ddE. Black line represents Youden’s index and blue line represents distance between the left upper corner, 100% true positive rate and 0% false positive rate, and plots on the ROC curve. Youden’s index became plateau exceeding 0.48 at a range from -5.64 to -2.75 kcal/mol cutoff values and the biggest at -4.86 kcal/mol. The distance were less than 0.38 from -5.64 to -1.87 kcal/mol cutoff values and became the shortest at -4.86 kcal/mol

An ROC curve was drawn in Fig. 2 using the working dataset, which consisted of 832 PAAs derived from the total dataset by excluding compounds with non-aromatic amine alerts, ring opening remarks, CH_2_CH_3_ or larger ortho substituents, or MWs > 500. The distance between the ROC curve and the left upper corner, which represents 100% true positives and 0% false positives, and Youden’s index at various ddE cutoff values are shown in Fig. 3. The distance below the bottom line was 0.38 when the ddE cutoff values were between -1.87 kcal/mol and -5.64 kcal/mol, with the shortest distance having a cutoff value of -4.86 kcal/mol. Youden’s index increased to 0.48 and plateaued when the cutoff values ranged from -2.75 kcal/mol to -5.64 kcal/mol, reaching its highest value at the -4.86 kcal/mol cutoff.

### Prediction performances for 824 PAAs finally assessed by ddE values

**Fig. 4:**
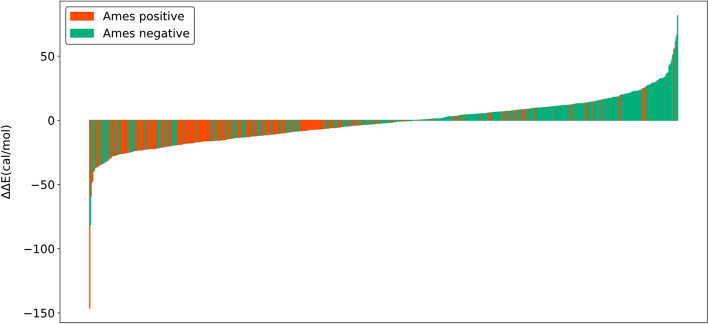
Ames test results and ddE values of 824 PAAs in Table 6

Table 6 displays the performance metrics at various ddE cutoff values with 2.5 kcal/mol separation of 824 ddE computable PAAs that were MW ≤ 500 without two or more amine groups, non-PAA structure alert, two large ortho substituted groups, or ring opening system. Both balanced accuracy and overall accuracy were highest when the cutoff value was -5 kcal/mol. The MCC was the highest at the 0 kcal/mol cutoff value. Ames test results and ddE values were visualized in Fig. 4.

**Table 6:** Prediction performance with various ddE threshold values with ddE-classified PAAs

ddE cut-off value (kcal/moll)	10	7.5	5	2.5	0	-2.5	-5	-7.5	-10
Sensitivity (%)	93.8	91.0	88.2	85.4	82.6	77.3	72.0	61.1	54.2
Specificity (%)	33.0	40.0	47.3	54.9	62.2	69.4	75.9	78.3	81.3
Positive prediction value (%)	47.2	49.2	51.6	54.7	58.2	61.7	65.6	64.3	64.9
Negative prediction value (%)	89.2	87.4	86.2	85.4	84.8	82.7	80.9	75.9	73.6
Accuracy (%)	56.7	59.8	63.2	66.7	70.1	72.5	74.4	71.6	70.8
Balanced accuracy (%)	63.4	65.5	67.7	70.1	72.4	73.3	74.0	69.7	67.8
Mathews correlation coefficient	0.37	0.37	0.38	0.38	0.39	0.37	0.36	0.29	0.25
Positive likelihood ratio	1.40	1.52	1.67	1.89	2.19	2.53	2.99	2.82	2.90
Negative likelihood ratio	0.19	0.23	0.25	0.27	0.28	0.33	0.37	0.50	0.56
Coverage^a^ (%)	100.0								

^a^Percentage of ddE computable PAAs, of which 40.0% Ames positive

## Discussion

**Fig. 5:**
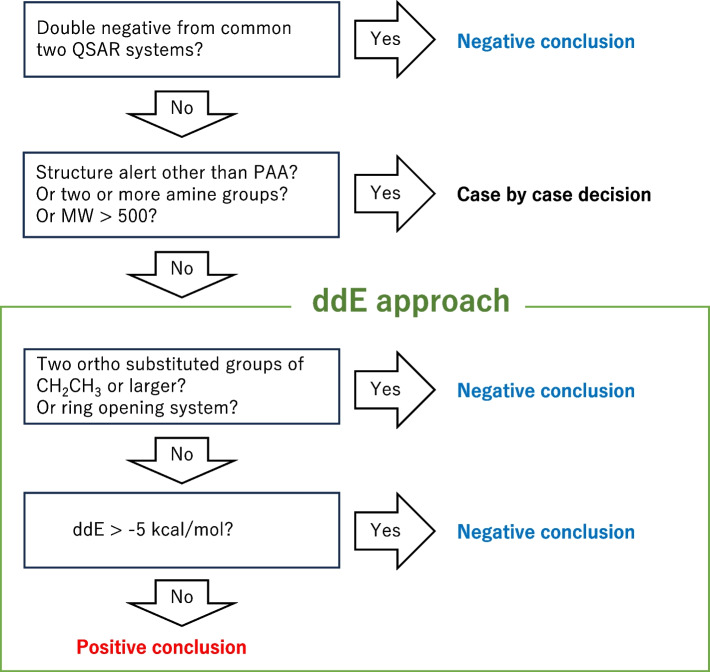
Proposed scheme of integration of the ddE approach into QSAR mutagenicity assessment of PAA

The results of this study indicated that the ddE approach improved the reliability of positive QSAR predictions. In order to compare the results in this study and literature data, we calculated the likelihood ratios that are not affected by prevalences of datasets [29]. LR + is a measure how much more likely a positive test result is in patients with the disease compared to those without it. In this study, a higher LR + indicates a higher probability that a query compound with positive prediction will test positive in the Ames test. A lower LR- indicates a higher probability that a query compound with negative prediction will be Ames test negative. LR + and LR- with -5 kcal/mol cutoff were 2.99 and 0.37 in this study (Table 6). When calculated from the previous report predicting Ames results of PAAs with commercially available three measure QSAR systems [22], LR + /LR- were 2.33/0.51, 1.46/0.61 and 2.62/0.34. This suggests that the proposed approach (Fig. 5) can provide helpful information for correcting false positive outcomes for PAAs from commonly used QSAR tools.

The calculated best cutoff value of ddE from the ROC curve (Fig. 2) was -4.86 kcal/mol. Youden’s index also indicated that -4.86 kcal/mol was the best-balanced cutoff value (Fig. 3). We determined that the suitable ddE cutoff point was -5 kcal/mol, rounded from -4.86 kcal/mol. The two indicators suggested that an appropriate range of cutoff value was between -2.75 kcal/mol and -5.64 kcal/mol. Our results were similar to those of Bentzien et al. [24] who reported that a -5.5 kcal/mol cutoff point for ddE led to a well-balanced prediction. Furukawa et al. [26] reported the best cutoff value of 2.5 kcal/mol via analysis of their internal PAAs, which was outside the reasonable range suggested in this study. Compared to the two cutoff values of 2.5 kcal/mol and -5 kcal/mol obtained in Table 6, the -5 kcal/mol cutoff point improved the PPV from 54.7% to 65.6%, with a reduction in the NPV from 85.4% to 80.9%. We consider an NPV of approximately 80% to be acceptable, as the reproducibility of Ames test results among research facilities was reported to be 80–85% [30]. Thus, a predictivity of approximately 80% is a realistic goal predicting Ames mutagenicity.

The results in this study revealed limitations of the ddE approach. The ddE approach was not effective to PAAs with MW > 500, two ortho substituted groups of CH_2_CH_3_ or larger, or ring opening system. The sensitivity, specificity, PPV, and NPV for PAAs with MWs > 500 were 50.0%, 40.0%, 10.0% and 85.7%, respectively (Table 4). The majority of positive calls from the ddE classification were false positives. Two reasons for the very low positive prediction performance of the ddE-classification were considered. One was permeability through the bacterial outer membrane. The ability of a test compound to permeate Ames tester strain cells was verified by its sensitivity to crystal violet (MW = 408) [31], and the size limit of molecules able to permeate the outer membrane has not yet been clarified [32]. The other reason may be difficulties in ddE calculations due to the structural complexity of larger molecules. A previous study of the ddE approach by Bentzien et al. [24] used only PAAs with a MW 500 and less. Although the threshold of MW could be more optimized, the dataset was insufficient in this study.

The presence of compounds with ring openings in ddE calculations can cause many false positives (Table 3). The ring opening data suggested that the nitrenium ion derived from the query compound was more stable in an open ring form, which is no longer an aromatic amine (Fig. 1). This explains the very low percentage of Ames-positive compounds (6/51 of ring-opening compounds).

The influence of ortho substituent groups should be considered in expert reviews together with ddE values. The ddE approach was based on the nitrenium ion hypothesis that PAAs are metabolized into nitrenium ions, which then react with DNA components. Steric hindrance from ortho position substituents may inhibit N-hydroxylation, the first step in the metabolic activation of aromatic amines [33]. Shamovsky et al. [34] suggested that structural modifications to prevent H-bonding or geometric fitting of PAA to CYP1A2 effectively prevents mutagenicity. Table 5 shows that large substituted groups at ortho positions increased the false positive predictions of the ddE approach.

We propose integrating the ddE-based approach into the QSAR mutagenicity assessment scheme for PAAs (Fig. 5). When two widely-used QSAR systems (knowledge-based and statistics-based) under ICH M7 provided consensus results on a PAA query, the NPV was 89%, and the PPV was 55% [22]. The consensus negative prediction from the two QSAR systems could be acceptable. If no consensus negative outcome is given, even if a consensus positive outcome is reached, further assessment is useful. The query compounds with a non-PAA alert, e.g., quinoline, aromatic nitro, or acid halide, derived from common QSAR systems or MWs > 500 falls beyond the scope of the ddE approach, and case-by-case decisions are needed.

For the query compounds with MWs ≤ 500, ddE values are calculated. Ortho substituents of ethyl-sized or larger groups increased Ames negative probability regardless of ddE values (Table 5). A ring opening remark also increased the probability of Ames negative results (Table 3). Either can be a good reason for negative conclusion. When the ddE value exceeds -5 kcal/mol, a negative conclusion is reasonable. When ddE is -5 kcal/mol or less for the query compounds with no or small ortho substituents, a positive conclusion is most plausible. Because the PPV was 65.6% in the ddE approach in this study (Table 6), one-third of the PAAs in this category were Ames negative, making the Ames test a good option.

The decision tree shown in Fig. 5 is one of the currently applicable approaches. The discussion on the activating and deactivating features of substituted groups of PAAs is an important part of the QSAR expert review. This study can provide a better understanding within the frequently required discussions in cases where activating and deactivating features exist simultaneously; however, the accuracy of QSAR prediction could be further improved. The influence of one large substituent at an ortho position remains unclear. The ddE calculation is not useful for estimating CYP inhibition. Feeney et al. [35] noted the difficulties of predicting metabolically activated mutagens such as PAAs and suggested the potential use of multiple-instance learning to determine metabolic contributions. These new approaches will enable the further improvement of QSAR predictions of the mutagenicity of PAAs.

## Conclusions

The results of this study suggest that the ddE approach is effective in reducing false positive calls in QSAR mutagenicity assessments for PAAs. We propose the integration of the ddE approach into the existing QSAR mutagenicity assessment scheme for PAAs.

## Supplementary Information

Additional file 1.

## Data Availability

The SVL script “mut_nitre.svl” can be obtained from MOLSIS Inc. Please contact ccg@molsis.co.jp to request it. Additional information on the PAAs used in this study can be found in the Supplemental data.

## Abbreviations

PAA: Primary aromatic amine
ICH: The International Council for Harmonisation of Technical Requirements for Pharmaceuticals for Human Use
MCC: Matthews correlation coefficient
MOE: Molecular operation environment
NIHS: National Institute of Health Sciences
NPV: Negative prediction value
PPV: Positive predictive value
QSAR: Quantitative structure–activity relationship
ROC: Receiver operating characteristic
