# Supplementary material for: Local QSAR based on quantum chemistry calculations for the stability of nitrenium ions to reduce false positive outcomes from standard QSAR systems for the mutagenicity of primary aromatic amines
Source: Genes Environ. 2024 Nov 21;46:24. doi: 10.1186/s41021-024-00318-4 (PMC11580225; doi:10.1186/s41021-024-00318-4)
Supplement: Supplementary file 1 — Additional file 1. [file 41021_2024_318_MOESM1_ESM.doc]

**Local QSAR based on quantum chemistry calculations for the stability of nitrenium ions to reduce false positive outcomes from standard QSAR systems for the mutagenicity of primary aromatic amines**

**Supplementary information of 1177 primary aromatic amines used in this study**

| ID | CAS No. | SMILES | MW | Ames | ddE  kcal/mol | remark | Ref |
| --- | --- | --- | --- | --- | --- | --- | --- |
|  | 57-67-0 | C=1C=C(C=CC=1N)S(NC(=N)N)(=O)=O | 214.24 | Negative | 16.7 |  | (1) |
|  | 57-68-1 | CC1=CC(C)=NC(=N1)NS(C2=CC=C(C=C2)N)(=O)=O | 278.33 | Negative | 12.9 |  | https://tools.niehs.nih.gov/cebs3/ntpViews/?activeTab=detail&studyNumber=768024 |
|  | 58-61-7 | C(C3C(C(C(N2C=NC1=C(N)N=CN=C12)O3)O)O)O | 267.24 | Negative | 8.7 |  | https://echa.europa.eu/nl/registration-dossier/-/registered-dossier/23062/7/7/2 |
|  | 60-27-5 | O=C1N=C(N)N(C)C1 | 113.12 | Negative | 66.5 |  | https://echa.europa.eu/registration-dossier/-/registered-dossier/16337/7/7/2 |
|  | 61-82-5 | \| 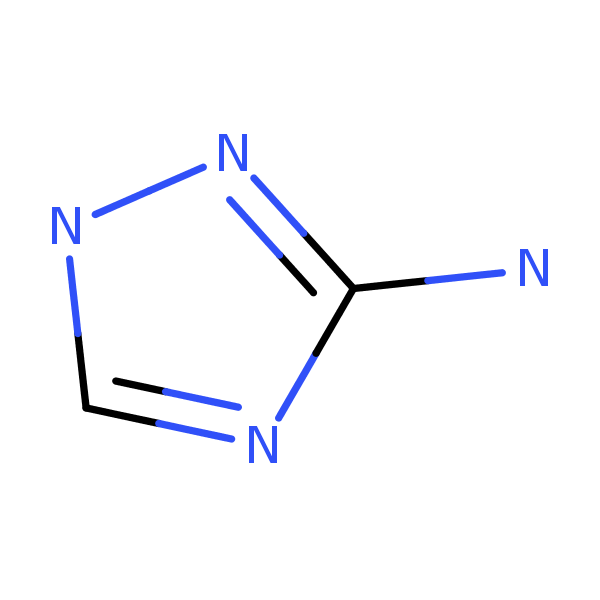C1=NC(N)=NN1 \| \| --- \| | 84.08 | Negative | 6.5 |  | https://echa.europa.eu/nl/registration-dossier/-/registered-dossier/21685/7/7/1 |
|  | 63-74-1 | C=1C=C(C=CC=1N)S(N)(=O)=O | 172.20 | Negative | 15.5 |  | (2) |
|  | 72-14-0 | C=1C=C(C=CC=1N)S(NC2=NC=CS2)(=O)=O | 255.32 | Negative | 13.1 |  | (2) |
|  | 81-49-2 | C1=CC=C3C(=C1)C(C=2C(=CC(=C(C=2C3=O)N)Br)Br)=O | 381.02 | Positive | 12.3 |  | http://tools.niehs.nih.gov/cebs3/ntpViews/?activeTab=detail&studyNumber=685556 |
|  | 82-28-0 | \| 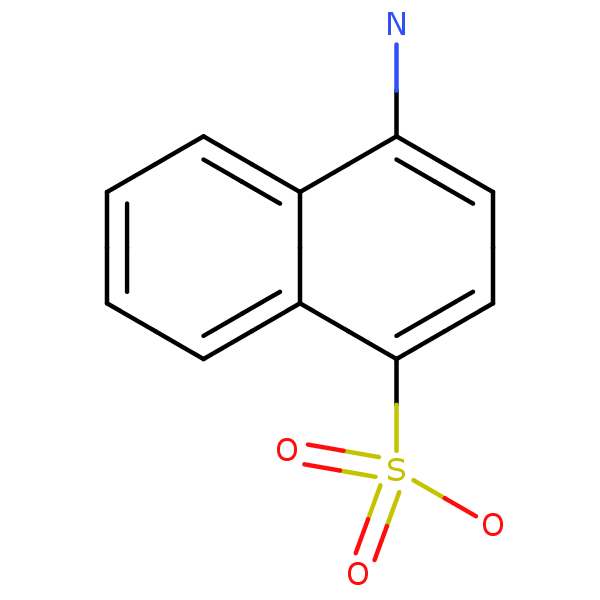CC=1C=CC3=C(C=1N)C(C2=CC=CC=C2C3=O)=O \| \| --- \| | 237.25 | Positive | 0.0 |  | https://tools.niehs.nih.gov//cebs3/ntpViews/?activeTab=detail&studyNumber=217835 |
|  | 84-86-6 | C1=CC=C2C(=C1)C(=CC=C2S(O)(=O)=O)N | 223.25 | Negative | 7.9 |  | https://echa.europa.eu/registration-dossier/-/registered-dossier/22359/7/7/2 |
|  | 85-84-7 | C1=CC=C(C=C1)N=NC=2C3=CC=CC=C3C=CC=2N | 247.30 | Negative | -24.2 |  | https://pubchem.ncbi.nlm.nih.gov/bioassay/1259407#sid=363901143 |
|  | 87-25-2 | CCOC(C1=CC=CC=C1N)=O | 165.19 | Negative | 6.5 |  | https://tools.niehs.nih.gov/cebs3/ntpViews/?activeTab=detail&studyNumber=824808 |
|  | 87-29-6 | C1=CC=C(C=C1)C=CCOC(C2=CC=CC=C2N)=O | 253.30 | Negative | 6.7 |  | https://tools.niehs.nih.gov/cebs3/ntpViews/?activeTab=detail&studyNumber=334090 |
|  | 87-59-2 | CC=1C=CC=C(C=1C)N | 121.18 | Positive | -6.9 |  | https://echa.europa.eu/registration-dossier/-/registered-dossier/11559/7/7/2 |
|  | 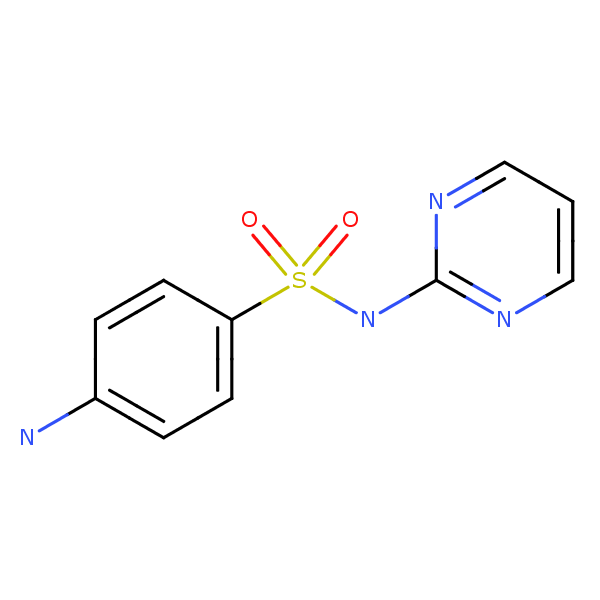   \| 87-60-5 \| \| --- \| | CC1=C(C=CC=C1N)Cl | 141.60 | Negative | -0.2 |  | https://tools.niehs.nih.gov/cebs3/ntpViews/?activeTab=detail&studyNumber=A84599 |
|  | 87-62-7 | CC=1C=CC=C(C)C=1N | 121.18 | Positive | -9.8 |  | https://echa.europa.eu/registration-dossier/-/registered-dossier/13610/7/7/2 |
|  | 88-05-1 | CC1=CC(C)=C(C(C)=C1)N | 135.21 | Positive | -15.4 |  | https://pubchem.ncbi.nlm.nih.gov/bioassay/1259407#sid=363900821 |
|  | 88-17-5 | C1=CC=C(C(=C1)C(F)(F)F)N | 161.12 | Negative | 14.4 |  | https://echa.europa.eu/registration-dossier/-/registered-dossier/11316/7/7/2 |
|  | 88-21-1 | C1=CC=C(C(=C1)N)S(O)(=O)=O | 173.19 | Negative | 20.2 |  | https://echa.europa.eu/registration-dossier/-/registered-dossier/10511/7/7/2 |
|  | 88-23-3 | C1=C(C=C(C(=C1N)O)S(O)(=O)=O)Cl | 223.63 | Negative | 0.2 |  | https://tools.niehs.nih.gov/cebs3/ntpViews/?activeTab=detail&studyNumber=496798 |
|  | 88-44-8 | CC=1C=CC(=C(C=1)S(O)(=O)=O)N | 187.22 | Negative | 13.1 |  | https://echa.europa.eu/registration-dossier/-/registered-dossier/13513/7/7/2 |
|  | 88-51-7 | CC1=CC(=C(C=C1Cl)N)S(O)(=O)=O | 221.66 | Negative | 17.9 |  | https://echa.europa.eu/registration-dossier/-/registered-dossier/13749/7/7/2 |
|  | 88-53-9 | CC1=CC(=C(C=C1Cl)S(O)(=O)=O)N | 221.66 | Negative | 18.1 |  | https://echa.europa.eu/registration-dossier/-/registered-dossier/16441/7/7/2 |
|  | 89-57-6 | C1=CC(=C(C=C1N)C(O)=O)O | 153.14 | Negative | -8.1 |  | https://echa.europa.eu/registration-dossier/-/registered-dossier/5887/7/7/2 |
|  | 90-04-0 | COC1=CC=CC=C1N | 123.15 | Positive | -15.8 |  | https://echa.europa.eu/nl/registration-dossier/-/registered-dossier/13498/7/7/2 |
|  | 91-59-8 | C1=CC=C2C=C(C=CC2=C1)N | 143.19 | Positive | -11.0 |  | https://tools.niehs.nih.gov/cebs3/ntpViews/?activeTab=detail&studyNumber=828041 |
|  | 92-36-4 | CC=1C=CC3=C(C=1)SC(C2=CC=C(C=C2)N)=N3 | 240.32 | Positive | -10.9 |  | https://echa.europa.eu/nl/registration-dossier/-/registered-dossier/10982/7/7/2 |
|  | 92-67-1 | C1=CC=C(C=C1)C2=CC=C(C=C2)N | 169.22 | Positive | -12.5 |  | https://tools.niehs.nih.gov//cebs3/ntpViews/?activeTab=detail&studyNumber=121112 |
|  | 93-05-0 | CCN(CC)C=1C=CC(=CC=1)N | 164.25 | Positive | -36.2 |  | https://tools.niehs.nih.gov/cebs3/ntpViews/?activeTab=detail&studyNumber=236524 |
|  | 94-25-7 | CCCCOC(C1=CC=C(C=C1)N)=O | 193.24 | Negative | 8.8 |  | https://tools.niehs.nih.gov/cebs3/ntpViews/?activeTab=detail&studyNumber=795473 |
|  | 94-45-1 | CCOC=1C=CC2=C(C=1)SC(N)=N2 | 194.25 | Positive | -17.6 |  | http://tools.niehs.nih.gov/cebs3/ntpViews/?activeTab=detail&studyNumber=248723 |
|  | 94-70-2 | CCOC1=CC=CC=C1N | 137.18 | Positive | -17.8 |  | https://tools.niehs.nih.gov/cebs3/ntpViews/?activeTab=detail&studyNumber=557646 |
|  | 95-23-8 | C1=CC2=C(C=C1N)NC(N2)=O | 149.15 | Negative | -14.0 |  | https://echa.europa.eu/nl/registration-dossier/-/registered-dossier/1949/7/7/2 |
|  | 95-51-2 | C1=CC=C(C(=C1)Cl)N | 127.57 | Negative | 1.1 |  | https://echa.europa.eu/nl/registration-dossier/-/registered-dossier/13785/7/7/2 |
|  | 95-53-4 | CC1=CC=CC=C1N | 107.15 | Positive | -5.6 |  | https://echa.europa.eu/nl/registration-dossier/-/registered-dossier/14441/7/7/2 |
|  | 95-55-6 | C1=CC=C(C(=C1)N)O | 109.13 | Positive | -12.5 |  | https://tools.niehs.nih.gov/cebs3/ntpViews/?activeTab=detail&studyNumber=523472 |
|  | 95-64-7 | CC1=CC=C(C=C1C)N | 121.18 | Positive | -8.0 |  | https://tools.niehs.nih.gov/cebs3/ntpViews/?activeTab=detail&studyNumber=181962 |
|  | 95-68-1 | CC=1C=CC(=C(C)C=1)N | 121.18 | Positive | -11.5 |  | https://tools.niehs.nih.gov/cebs3/ntpViews/?activeTab=detail&studyNumber=330987 |
|  | 95-69-2 | CC1=CC(=CC=C1N)Cl | 141.60 | Positive | -5.4 |  | (3) |
|  | 95-74-9 | CC=1C=CC(=CC=1Cl)N | 141.60 | Negative | -1.6 |  | https://tools.niehs.nih.gov/cebs3/ntpViews/?activeTab=detail&studyNumber=991791 |
|  | 95-76-1 | C1=CC(=C(C=C1N)Cl)Cl | 162.02 | Negative | 4.8 |  | https://tools.niehs.nih.gov/cebs3/ntpViews/?activeTab=detail&studyNumber=623426 |
|  | 95-78-3 | CC=1C=CC(C)=C(C=1)N | 121.18 | Positive | -7.2 |  | https://tools.niehs.nih.gov/cebs3/ntpViews/?activeTab=detail&studyNumber=330481 |
|  | 95-79-4 | CC=1C=CC(=CC=1N)Cl | 141.60 | Negative | -0.6 |  | https://tools.niehs.nih.gov/cebs3/ntpViews/?activeTab=detail&studyNumber=092475 |
|  | 95-82-9 | C1=CC(=C(C=C1Cl)N)Cl | 162.02 | Negative | 6.0 |  | https://tools.niehs.nih.gov/cebs3/ntpViews/?activeTab=detail&studyNumber=512154 |
|  | 95-84-1 | CC=1C=CC(=C(C=1)N)O | 123.15 | Positive | -14.2 |  | https://tools.niehs.nih.gov/cebs3/ntpViews/?activeTab=detail&studyNumber=206555 |
|  | 95-85-2 | C1=CC(=C(C=C1Cl)N)O | 143.57 | Positive | -8.1 |  | https://tools.niehs.nih.gov/cebs3/ntpViews/?activeTab=detail&studyNumber=646474 |
|  | 96-50-4 | C1=CSC(N)=N1 | 100.14 | Positive | -8.0 |  | https://tools.niehs.nih.gov/cebs3/ntpViews/?activeTab=detail&studyNumber=636072 |
|  | 97-56-3 | CC1=CC=CC=C1N=NC2=CC=C(C(C)=C2)N | 225.29 | Positive | -14.4 |  | https://tools.niehs.nih.gov/cebs3/ntpViews/?activeTab=detail&studyNumber=222957 |
|  | 98-16-8 | C1=CC(=CC(=C1)N)C(F)(F)F | 161.12 | Negative | 11.5 |  | https://tools.niehs.nih.gov/cebs3/ntpViews/?activeTab=detail&studyNumber=758178 |
|  | 98-30-6 | CS(C=1C=CC(=C(C=1)N)O)(=O)=O | 187.22 | Negative | -3.8 |  | https://tools.niehs.nih.gov/cebs3/ntpViews/?activeTab=detail&studyNumber=433469 |
|  | 98-33-9 | CC1=CC(=CC=C1N)S(O)(=O)=O | 187.22 | Negative | 17.4 |  | https://echa.europa.eu/nl/registration-dossier/-/registered-dossier/17643/7/7/2 |
|  | 98-37-3 | C1=CC(=C(C=C1S(O)(=O)=O)N)O | 189.19 | Negative | -0.8 |  | https://tools.niehs.nih.gov/cebs3/ntpViews/?activeTab=detail&studyNumber=941907 |
|  | 99-88-7 | CC(C)C1=CC=C(C=C1)N | 135.21 | Positive | -7.1 |  | https://echa.europa.eu/nl/registration-dossier/-/registered-dossier/10013/7/7/2 |
|  | 99-92-3 | \| 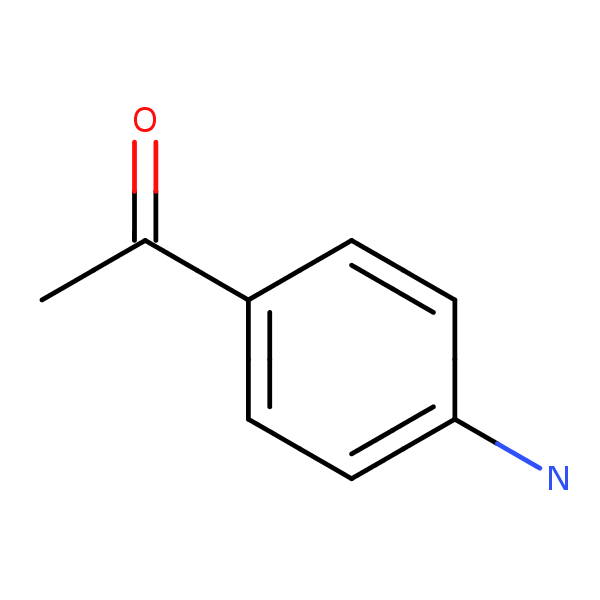CC(C1=CC=C(C=C1)N)=O \| \| --- \| | 135.16 | Negative | 7.4 |  | https://pubchem.ncbi.nlm.nih.gov/bioassay/1259407#sid=363903011 |
|  | 99-98-9 | CN(C)C1=CC=C(C=C1)N | 136.19 | Positive | -34.3 |  | https://tools.niehs.nih.gov/cebs3/ntpViews/?activeTab=detail&studyNumber=655233 |
|  | 101-54-2 | C1=CC=C(C=C1)NC2=CC=C(C=C2)N | 184.24 | Negative | -32.7 |  | https://tools.niehs.nih.gov/cebs3/ntpViews/?activeTab=detail&studyNumber=231064 |
|  | 102-28-3 | CC(NC1=CC=CC(=C1)N)=O | 150.18 | Positive | -1.6 |  | https://tools.niehs.nih.gov/cebs3/ntpViews/?activeTab=detail&studyNumber=908000 |
|  | 102-50-1 | CC1=CC(=CC=C1N)OC | 137.18 | Positive | -20.4 |  | https://tools.niehs.nih.gov/cebs3/ntpViews/?activeTab=detail&studyNumber=469273 |
|  | 102-56-7 | COC=1C=CC(=C(C=1)N)OC | 153.18 | Negative | -12.9 |  | https://echa.europa.eu/nl/registration-dossier/-/registered-dossier/5256/7/7/2 |
|  | 104-23-4 | C=1C=C(C=CC=1N)N=NC2=CC=C(C=C2)S(O)(=O)=O | 277.30 | Negative | -2.9 |  | https://echa.europa.eu/nl/registration-dossier/-/registered-dossier/5637/7/7/2 |
|  | 104-94-9 | COC1=CC=C(C=C1)N | 123.15 | Positive | -16.7 |  | https://pubchem.ncbi.nlm.nih.gov/bioassay/1259407#sid=363898867 |
|  | 106-40-1 | C=1C=C(C=CC=1Br)N | 172.02 | Negative | 3.8 |  | https://tools.niehs.nih.gov//cebs3/ntpViews/?activeTab=detail&studyNumber=900944 |
|  | 106-47-8 | C=1C=C(C=CC=1Cl)N | 127.57 | Positive | 0.4 |  | https://tools.niehs.nih.gov/cebs3/ntpViews/?activeTab=detail&studyNumber=619172 |
|  | 106-49-0 | \| 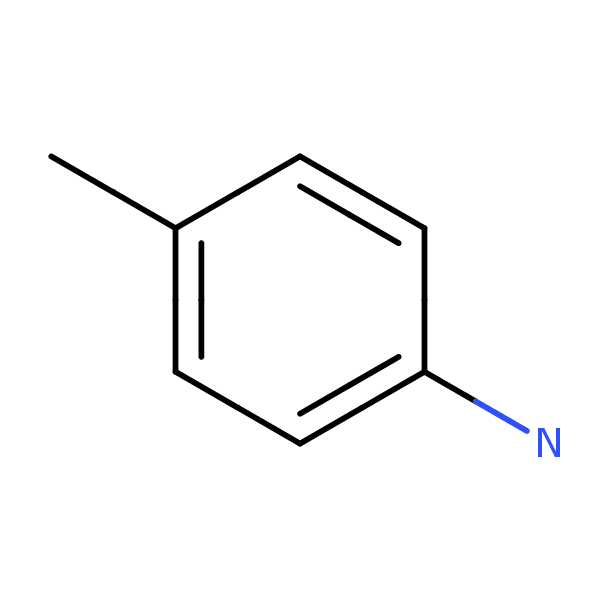CC1=CC=C(C=C1)N \| \| --- \| | 107.15 | Positive | -6.8 |  | https://echa.europa.eu/nl/registration-dossier/-/registered-dossier/14581/7/7/2 |
|  | 108-42-9 | C1=CC(=CC(=C1)N)Cl | 127.57 | Negative | 5.4 |  | https://tools.niehs.nih.gov/cebs3/ntpViews/?activeTab=detail&studyNumber=309708 |
|  | 108-44-1 | CC=1C=CC=C(C=1)N | 107.15 | Negative | -1.6 |  | https://echa.europa.eu/registration-dossier/-/registered-dossier/18872/7/7/2 |
|  | 108-69-0 | CC1=CC(C)=CC(=C1)N | 121.18 | Positive | -3.1 |  | https://echa.europa.eu/registration-dossier/-/registered-dossier/12529/7/7/2 |
|  | 116-83-6 | COC=1C=CC(=C3C=1C(C2=CC=CC=C2C3=O)=O)N | 253.25 | Negative | 0.4 |  | https://pubchem.ncbi.nlm.nih.gov/bioassay/1259408#sid=363895345 |
|  | 116-85-8 | C1=CC=C3C(=C1)C(C2=C(C=CC(=C2C3=O)O)N)=O | 239.23 | Positive | -7.9 |  | https://pubchem.ncbi.nlm.nih.gov/bioassay/1259407#sid=363903551 |
|  | 117-79-3 | C1=CC=C3C(=C1)C(C=2C=CC(=CC=2C3=O)N)=O | 223.23 | Positive | 8.5 |  | https://pubchem.ncbi.nlm.nih.gov/bioassay/1259407#sid=363897973 |
|  | 118-92-3 | C1=CC=C(C(=C1)C(O)=O)N | 137.14 | Negative | 10.0 |  | https://pubchem.ncbi.nlm.nih.gov/bioassay/1259407#sid=363897999 |
|  | 120-71-8 | CC=1C=CC(=C(C=1)N)OC | 137.18 | Positive | -17.0 |  | http://tools.niehs.nih.gov/cebs3/ntpViews/?activeTab=detail&studyNumber=496780 |
|  | 121-47-1 | C1=CC(=CC(=C1)S(O)(=O)=O)N | 173.19 | Negative | 10.7 |  | https://pubchem.ncbi.nlm.nih.gov/bioassay/1259407#sid=363900710 |
|  | 121-50-6 | C1=CC(=C(C=C1C(F)(F)F)N)Cl | 195.57 | Negative | 11.7 |  | https://pubchem.ncbi.nlm.nih.gov/bioassay/1259407#sid=363900766 |
|  | 121-57-3 | C=1C=C(C=CC=1N)S(O)(=O)=O | 173.19 | Negative | 24.1 |  | http://tools.niehs.nih.gov/cebs3/ntpViews/?activeTab=detail&studyNumber=248635 |
|  | 122-80-5 | CC(NC1=CC=C(C=C1)N)=O | 150.18 | Positive | -22.5 |  | https://tools.niehs.nih.gov/cebs3/ntpViews/?activeTab=detail&studyNumber=122116 |
|  | 123-30-8 | C=1C=C(C=CC=1N)O | 109.13 | Positive | -12.7 |  | https://echa.europa.eu/nl/registration-dossier/-/registered-dossier/13807/7/7/2 |
|  | 127-69-5 | CC=1C(C)=NOC=1NS(C2=CC=C(C=C2)N)(=O)=O | 267.30 | Negative | 21.5 |  | http://tools.niehs.nih.gov/cebs3/ntpViews/?activeTab=detail&studyNumber=627464 |
|  | 130-17-6 | CC=1C=CC3=C(C=1S(O)(=O)=O)SC(C2=CC=C(C=C2)N)=N3 | 320.39 | Positive | -7.4 |  | http://tools.niehs.nih.gov/cebs3/ntpViews/?activeTab=detail&studyNumber=672157 |
|  | 132-32-1 | CCN2C3=CC=CC=C3C=1C=C(C=CC=12)N | 210.27 | Positive | -30.6 |  | http://tools.niehs.nih.gov/cebs3/ntpViews/?activeTab=detail&studyNumber=918212 |
|  | 133-18-6 | C1=CC=C(C=C1)CCOC(C2=CC=CC=C2N)=O | 241.29 | Negative | 6.5 |  | https://tools.niehs.nih.gov/cebs3/ntpViews/?activeTab=detail&studyNumber=819460 |
|  | 133-90-4 | C1=C(C=C(C(=C1C(O)=O)Cl)N)Cl | 206.03 | Positive | 10.7 |  | https://tools.niehs.nih.gov/cebs3/ntpViews/?activeTab=detail&studyNumber=537889 |
|  | 134-09-8 | CC(C)C1CCC(C)CC1OC(C2=CC=CC=C2N)=O | 275.39 | Negative | 4.9 |  | https://pubchem.ncbi.nlm.nih.gov/bioassay/1259407#sid=363900418 |
|  | 134-20-3 | \| 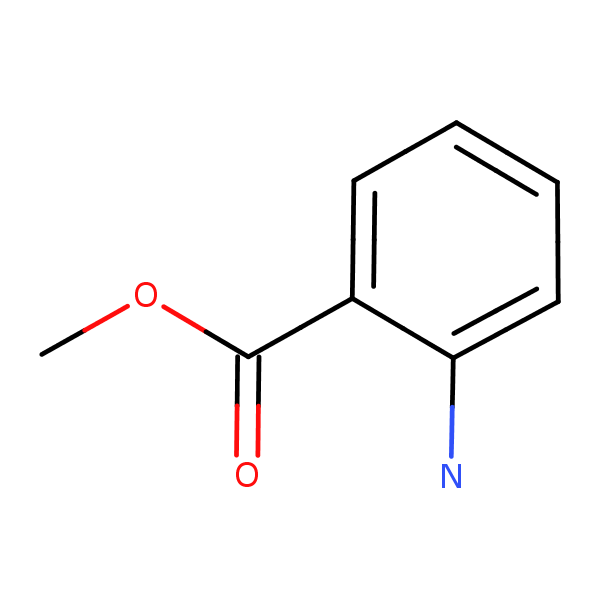COC(C1=CC=CC=C1N)=O \| \| --- \| | 151.16 | Negative | 7.4 |  | https://tools.niehs.nih.gov/cebs3/ntpViews/?activeTab=detail&studyNumber=118427 |
|  | 134-32-7 | C1=CC=C2C(=C1)C=CC=C2N | 143.19 | Positive | -15.5 |  | https://tools.niehs.nih.gov/cebs3/ntpViews/?activeTab=detail&studyNumber=979469 |
|  | 134-50-9 | C=1C=CC=3C(C=1)=C(C2=CC=CC=C2N=3)N | 194.23 | Positive | -16.4 |  | https://pubchem.ncbi.nlm.nih.gov/bioassay/1259407#sid=363904158 |
|  | 135-68-2 | C=1C=C(C=CC=1C2=CC=C(C=C2)N)Cl | 203.67 | Positive | -10.3 |  | https://pubchem.ncbi.nlm.nih.gov/bioassay/1259407#sid=363903094 |
|  | 137-07-5 | C1=CC=C(C(=C1)N)S | 125.19 | Positive | -15.5 |  | https://pubchem.ncbi.nlm.nih.gov/substance/363907043 |
|  | 137-17-7 | CC1=CC(C)=C(C=C1C)N | 135.21 | Positive | -12.8 |  | (4) |
|  | 139-59-3 | C1=CC=C3C(=C1)C=2C=CC(=CC=2N3)N | 182.22 | Positive | -17.4 |  | https://pubchem.ncbi.nlm.nih.gov/bioassay/1259407#sid=363904453 |
|  | 62-53-3 | C1=CC=C(C=C1)N | 93.13 | Negative | 0.0 |  | ICH M7 |
|  | 144-80-9 | CC(NS(C1=CC=C(C=C1)N)(=O)=O)=O | 214.24 | Negative | 23.5 |  | https://tools.niehs.nih.gov/cebs3/ntpViews/?activeTab=detail&studyNumber=411624 |
|  | 144-82-1 | CC2=NN=C(NS(C1=CC=C(C=C1)N)(=O)=O)S2 | 270.33 | Negative | 13.7 |  | https://tools.niehs.nih.gov/cebs3/ntpViews/?activeTab=detail&studyNumber=136744 |
|  | 144-83-2 | C1=CC=NC(=C1)NS(C2=CC=C(C=C2)N)(=O)=O | 249.29 | Negative | 11.4 |  | https://tools.niehs.nih.gov/cebs3/ntpViews/?activeTab=detail&studyNumber=635845 |
|  | 150-13-0 | C=1C=C(C=CC=1C(O)=O)N | 137.14 | Negative | 12.5 |  | https://tools.niehs.nih.gov/cebs3/ntpViews/?activeTab=detail&studyNumber=413543 |
|  | 153-78-6 | C2C=1C=CC=CC=1C=3C2=CC(=CC=3)N | 181.23 | Positive | -16.5 |  | https://pubchem.ncbi.nlm.nih.gov/bioassay/1259407#sid=363897981 |
|  | 156-43-4 | CCOC1=CC=C(C=C1)N | 137.18 | Positive | -18.8 |  | https://tools.niehs.nih.gov/cebs3/ntpViews/?activeTab=detail&studyNumber=276346 |
|  | 209-594-8 | CCC=1C=CC=C(C=1)N | 121.18 | Negative | -2.2 |  | https://tools.niehs.nih.gov/cebs3/ntpViews/?activeTab=detail&studyNumber=G08030 |
|  | 319-24-4 | COC(C=1C=C(C=CC=1N)F)=O | 169.15 | Negative | 6.3 |  | https://echa.europa.eu/nl/registration-dossier/-/registered-dossier/11929/7/7/2 |
|  | 320-51-4 | C1=CC(=C(C=C1N)C(F)(F)F)Cl | 195.57 | Negative | 10.4 |  | https://echa.europa.eu/nl/registration-dossier/-/registered-dossier/18462/7/7/1 |
|  | 348-40-3 | C1=CC2=C(C=C1F)SC(N)=N2 | 168.19 | Negative | -4.6 |  | (5) |
|  | 363-80-4 | C1=C(C=C(C(=C1F)F)N)F | 147.10 | Negative | 17.8 |  | (5) |
|  | 367-25-9 | C1=CC(=C(C=C1F)F)N | 129.11 | Negative | 0.4 |  | https://echa.europa.eu/nl/registration-dossier/-/registered-dossier/24338/7/7/2 |
|  | 367-34-0 | C1=C(C(=CC(=C1F)N)F)F | 147.10 | Negative | 8.7 |  | (5) |
|  | 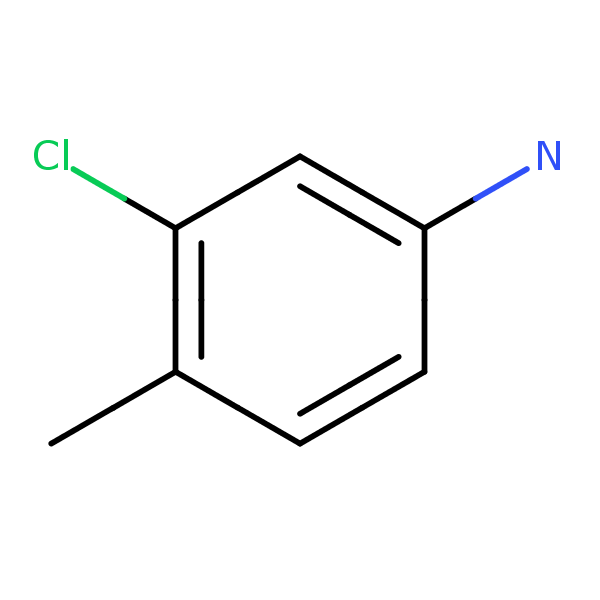   \| 399-95-1 \| \| --- \| | C1=CC(=C(C=C1O)F)N | 127.12 | Positive | -10.9 |  | https://echa.europa.eu/nl/registration-dossier/-/registered-dossier/6643/7/7/2 |
|  | 434-76-4 | C1=CC(=C(C(=C1)N)C(O)=O)F | 155.13 | Negative | 17.1 |  | (6) |
|  | 445-03-4 | \| 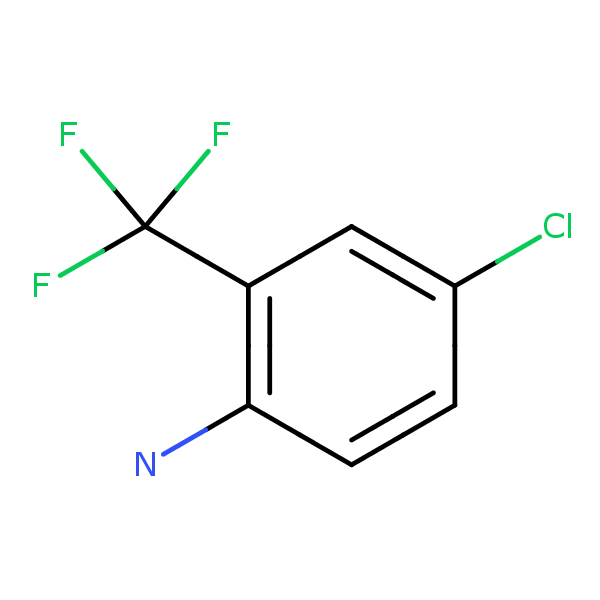C1=CC(=C(C=C1Cl)C(F)(F)F)N \| \| --- \| | 195.57 | Negative | 13.8 |  | https://echa.europa.eu/nl/registration-dossier/-/registered-dossier/12399/7/7/2 |
|  | 446-32-2 | C1=CC(=C(C=C1F)N)C(O)=O | 155.13 | Negative | 19.6 |  | (6) |
|  | 450-91-9 | COC=1C=C(C=CC=1N)F | 141.14 | Positive | -15.0 |  | (5) |
|  | 452-06-2 | C2=C1C(N=CN1)=NC(N)=N2 | 135.13 | Positive | 11.8 |  | https://pubchem.ncbi.nlm.nih.gov/bioassay/1259407#sid=363898709 |
|  | 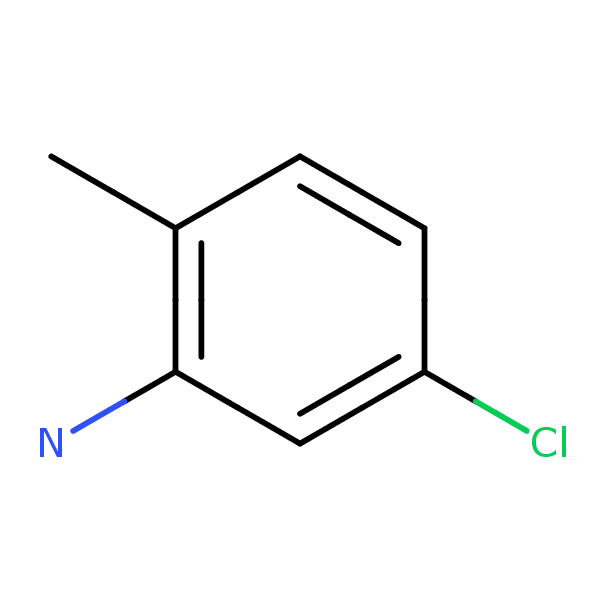452-58-4 | C1=CC(=C(N)N=C1)N | 109.13 | Positive | -27.5 | Two or more amines detected. The first site is used. | (6) |
|  | 455-14-1 | C=1C=C(C=CC=1C(F)(F)F)N | 161.12 | Positive | 16.6 |  | https://echa.europa.eu/registration-dossier/-/registered-dossier/12129/7/7/2 |
|  | 461-82-5 | \| 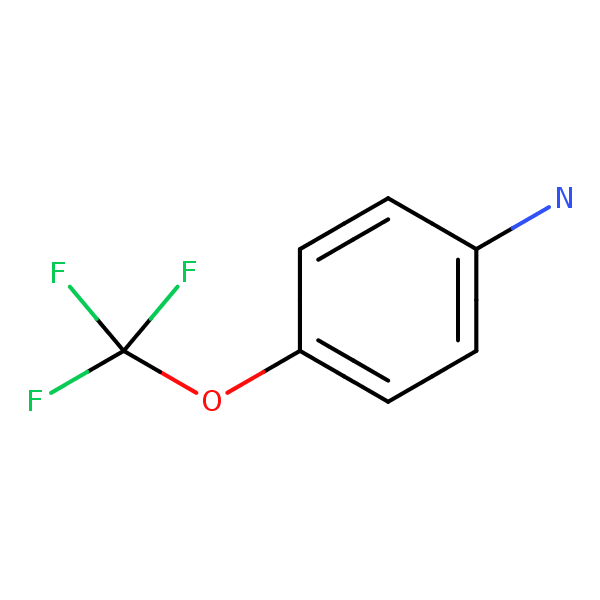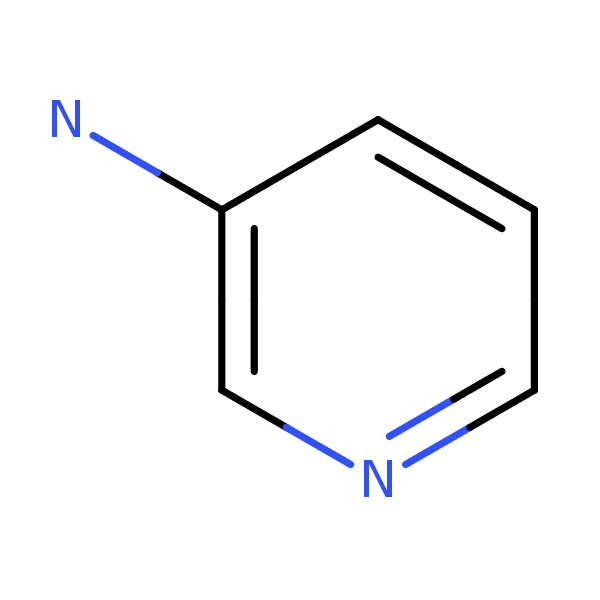C=1C=C(C=CC=1N)OC(F)(F)F \| \| --- \| | 177.12 | Negative | -3.0 |  | https://echa.europa.eu/nl/registration-dossier/-/registered-dossier/11758/7/7/2 |
|  | 462-08-8 | C1=CC(=CN=C1)N | 94.11 | Negative | 5.2 |  | https://echa.europa.eu/nl/registration-dossier/-/registered-dossier/16757/7/7/2 |
|  | 521-31-3 | C1=CC2=C(C(=C1)N)C(NNC2=O)=O | 177.16 | Negative | 13.1 |  | http://tools.niehs.nih.gov/cebs3/ntpViews/?activeTab=detail&studyNumber=108538 |
|  | 527-62-8 | C1=C(C=C(C(=C1Cl)O)N)Cl | 178.02 | Negative | -2.1 |  | (7) |
|  | 536-90-3 | \| 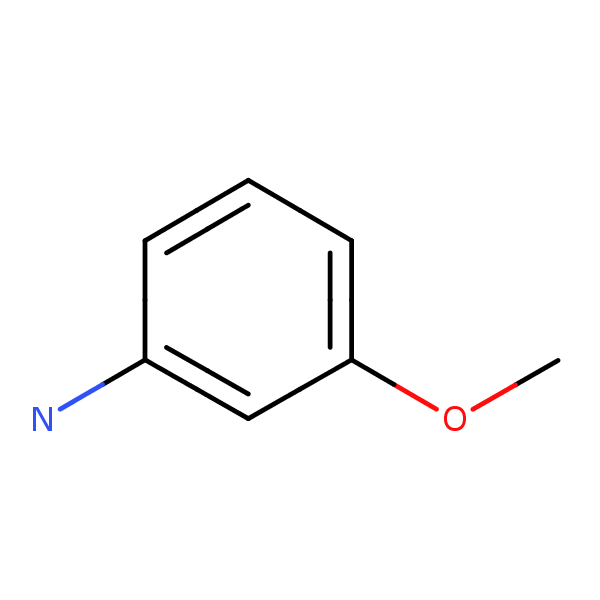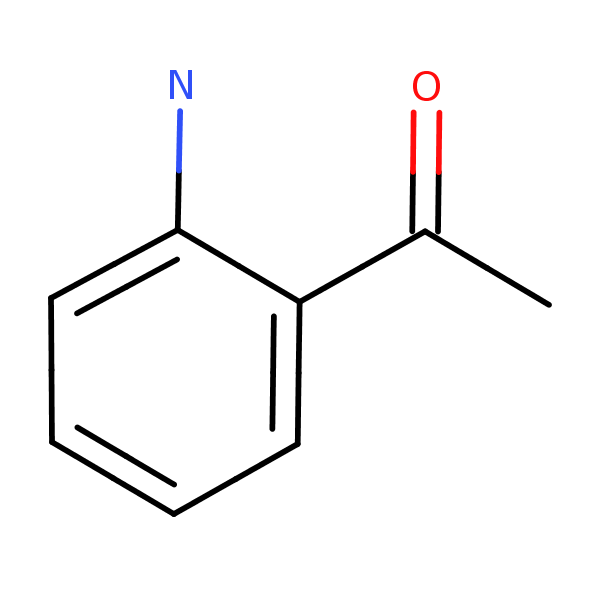COC1=CC=CC(=C1)N \| \| --- \| | 123.15 | Negative | 2.1 |  | http://tools.niehs.nih.gov/cebs3/ntpViews/?activeTab=detail&studyNumber=991373 |
|  | 551-93-9 | CC(C1=CC=CC=C1N)=O | 135.16 | Negative | 5.5 |  | https://echa.europa.eu/registration-dossier/-/registered-dossier/20816/7/7/2 |
|  | 553-24-2 | CC1=CC3=C(C=C1N)N=C2C=C(C=CC2=N3)N(C)C | 252.31 | Positive | -11.4 |  | (8) |
|  | 554-00-7 | C1=CC(=C(C=C1Cl)Cl)N | 162.02 | Negative | 1.3 |  | https://pubchem.ncbi.nlm.nih.gov/bioassay/1259407#sid=363903962 |
|  | 556-18-3 | C=1C=C(C=CC=1C=O)N | 121.14 | Negative | 9.7 |  | https://pubchem.ncbi.nlm.nih.gov/bioassay/1259407#sid=363906834&section=Version |
|  | 565-20-8 | CC(NC1=CC=C(C=C1)S(C2=CC=C(C=C2)N)(=O)=O)=O | 290.34 | Negative | 8.3 |  | (9) |
|  | 578-06-3 | C1=CC=C3C(=C1)C=C2C(=CC=CC2=N3)N | 194.23 | Positive | -15.0 |  | (10) |
|  | 578-54-1 | CCC1=CC=CC=C1N | 121.18 | Negative | -5.6 |  | https://tools.niehs.nih.gov/cebs3/ntpViews/?activeTab=detail&studyNumber=G08029 |
|  | \| 578-66-5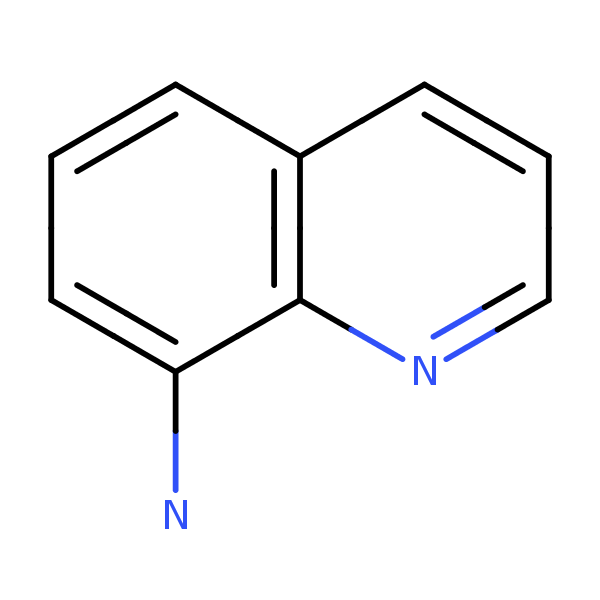 \| \| --- \| | NC1=CC=CC2=C1N=CC=C2 | 144.17 | Positive | -12.8 |  | (11) |
|  | 578-68-7 | C1=CC=C2C(=C1)C(=CC=N2)N | 144.17 | Negative | 3.5 |  | (11) |
|  | 579-66-8 | CCC=1C=CC=C(CC)C=1N | 149.23 | Negative | -10.2 |  | https://tools.niehs.nih.gov/cebs3/ntpViews/?activeTab=detail&studyNumber=G09035 |
|  | 580-15-4 | C2=CC1=CC(=CC=C1N=C2)N | 144.17 | Negative | -6.4 |  | (11) |
|  | 580-17-6 | C1=CC=C2C(=C1)C=C(C=N2)N | 144.17 | Positive | -6.5 |  | (11) |
|  | 580-19-8 | C2=CC=1C=CC(=CC=1N=C2)N | 144.17 | Positive | -2.6 |  | (11) |
|  | 580-22-3 | C1=CC=C2C(=C1)C=CC(N)=N2 | 144.17 | Positive | 4.4 |  | (11) |
|  | 581-28-2 | C1=CC=C3C(=C1)C=C2C=C(C=CC2=N3)N | 194.23 | Positive | -14.8 |  | (10) |
|  | 581-29-3 | C1=CC=C3C(=C1)C=C2C=CC(=CC2=N3)N | 194.23 | Positive | -8.2 |  | (10) |
|  | 608-27-5 | \| 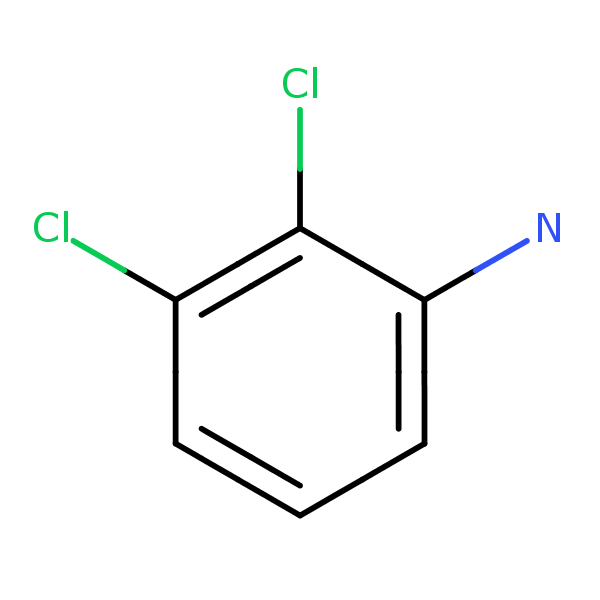C1=CC(=C(C(=C1)N)Cl)Cl \| \| --- \| | 162.02 | Negative | 5.2 |  | https://echa.europa.eu/nl/registration-dossier/-/registered-dossier/11877/7/7/2 |
|  | 610-49-1 | C2=CC=C3C=C1C(C=CC=C1N)=CC3=C2 | 193.24 | Positive | -23.7 |  | (12) |
|  | 611-34-7 | C1=CC(=C2C=CC=NC2=C1)N | 144.17 | Positive | -7.9 |  | (11) |
|  | 613-13-8 | C1=CC=C2C=C3C=C(C=CC3=CC2=C1)N | 193.24 | Positive | -18.4 |  | https://tools.niehs.nih.gov/cebs3/ntpViews/?activeTab=detail&studyNumber=153993 |
|  | 621-33-0 | CCOC1=CC=CC(=C1)N | 137.18 | Negative | 1.1 |  | https://pubchem.ncbi.nlm.nih.gov/bioassay/1259407#sid=363902646 |
|  | 626-43-7 | C1=C(C=C(C=C1Cl)N)Cl | 162.02 | Negative | 10.2 |  | https://tools.niehs.nih.gov/cebs3/ntpViews/?activeTab=detail&studyNumber=A40138 |
|  | 634-67-3 | C1=CC(=C(C(=C1Cl)Cl)Cl)N | 196.46 | Negative | 4.6 |  | https://pubchem.ncbi.nlm.nih.gov/bioassay/1259407#sid=363900347&section=Test-Results |
|  | 634-91-3 | C1=C(C=C(C(=C1Cl)Cl)Cl)N | 196.46 | Negative | 9.3 |  | https://pubchem.ncbi.nlm.nih.gov/bioassay/1259407#sid=363900348 |
|  | 634-93-5 | C1=C(C=C(C(=C1Cl)N)Cl)Cl | 196.46 | Negative | 2.8 |  | https://echa.europa.eu/registration-dossier/-/registered-dossier/16982/7/7/2 |
|  | 636-30-6 | \| 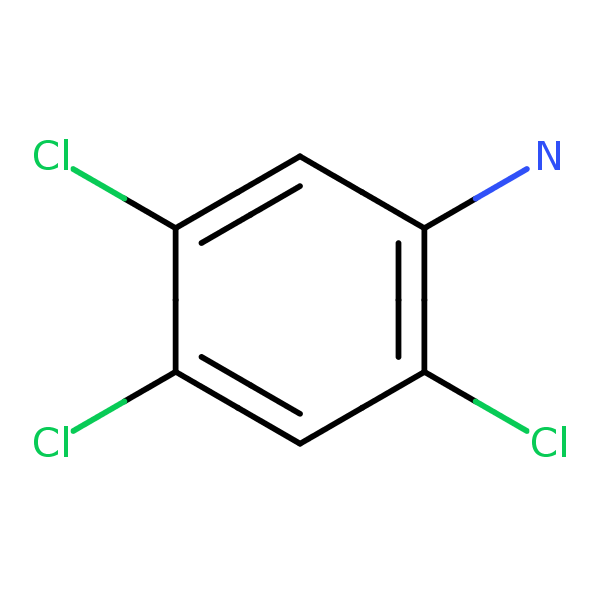C1=C(C(=CC(=C1Cl)N)Cl)Cl \| \| --- \| | 196.46 | Negative | 5.7 |  | https://echa.europa.eu/nl/registration-dossier/-/registered-dossier/13468/7/7/2 |
|  | 700-00-5 | CN2C=NC1=C(N)N=CN=C12 | 149.15 | Positive | 7.0 |  | (13) |
|  | 700-86-7 | C1=CC=2C(C=C1Cl)=NSC=2N | 184.65 | Positive | -16.0 |  | https://pubchem.ncbi.nlm.nih.gov/bioassay/1259407#sid=363906280 |
|  | 723-46-6 | CC1=CC(=NO1)NS(C2=CC=C(C=C2)N)(=O)=O | 253.28 | Negative | 14.3 |  | https://tools.niehs.nih.gov/cebs3/ntpViews/?activeTab=detail&studyNumber=215965 |
|  | 769-92-6 | \| 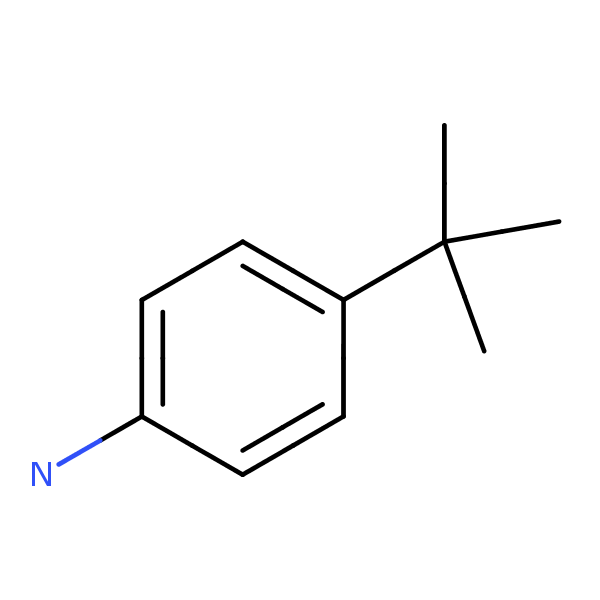CC(C)(C)C1=CC=C(C=C1)N \| \| --- \| | 149.23 | Positive | -8.2 |  | https://echa.europa.eu/nl/registration-dossier/-/registered-dossier/23433/7/7/2 |
|  | 779-03-3 | C=1C=CC=3C(C=1)=CC2=CC=CC=C2C=3N | 193.24 | Positive | -32.8 |  | (14) |
|  | 789-47-9 | C1=CC=C2C(=C1)C=CC=3C4=CC=C(C=C4C=CC2=3)N | 243.30 | Positive | -15.6 |  | (15) |
|  | 874-05-5 | C1=CC=C2C(=C1)C(N)=NN2 | 133.15 | Positive | -19.0 |  | (5) |
|  | 934-32-7 | C1=CC=C2C(=C1)NC(N)=N2 | 133.15 | Positive | -12.1 |  | (16) |
|  | \| 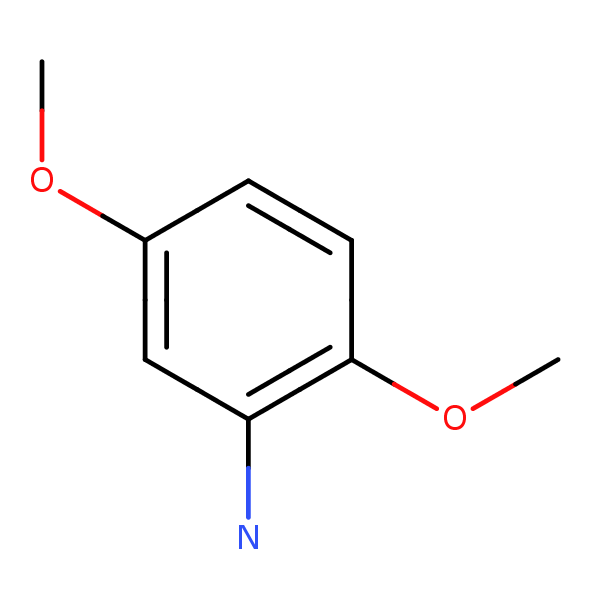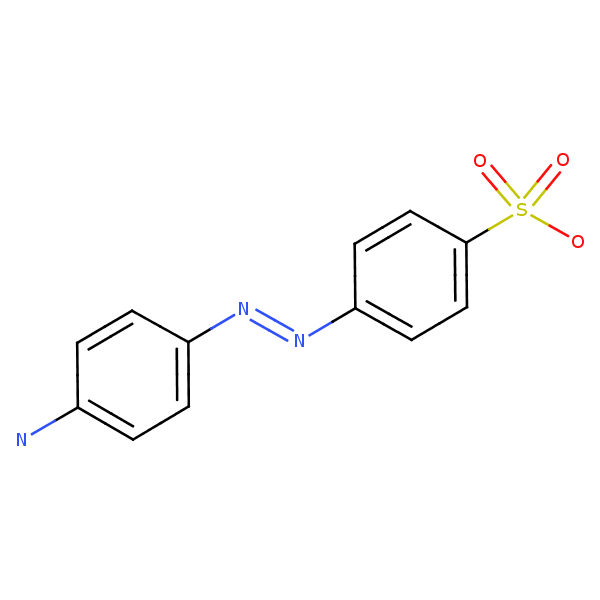947-73-9 \| \| --- \| | C1=CC=C2C(=C1)C=C(C3=CC=CC=C23)N | 193.24 | Positive | -19.8 |  | (14) |
|  | 958-09-8 | C1C(C(CO)OC1N3C=NC2=C(N)N=CN=C23)O | 251.24 | Negative | 9.6 |  | https://pubchem.ncbi.nlm.nih.gov/bioassay/1259407#sid=363899732 |
|  | 1120-99-6 | C1=CN=NC(=N1)N | 96.09 | Negative | 44.7 |  | (5) |
|  | 1134-94-7 | C1=CC=C(C=C1)SC2=CC=CC=C2N | 201.29 | Positive | -21.9 |  | https://echa.europa.eu/nl/registration-dossier/-/registered-dossier/13947/7/7/2/?documentUUID=b1c916cf-9f4f-40f3-9eb1-dc4590861b65 |
|  | 1135-14-4 | C1=CC=C(C=C1)SC2=CC=C(C=C2)N | 201.29 | Positive | -26.1 |  | (17) |
|  | 1137-41-3 | C1=CC=C(C=C1)C(C2=CC=C(C=C2)N)=O | 197.23 | Negative | 3.3 |  | (5) |
|  | 1747-60-0 | COC=1C=C2C(=CC=1)N=C(S2)N | 180.23 | Positive | -15.5 |  | https://tools.niehs.nih.gov//cebs3/ntpViews/?activeTab=detail&studyNumber=353634 |
|  | 1197-55-3 | C=1C=C(C=CC=1CC(O)=O)N | 151.16 | Positive | -0.4 |  | https://pubchem.ncbi.nlm.nih.gov/bioassay/1259407#sid=363901107 |
|  | 1198-40-9 | C1=CC2=C(C=CN=C2C=C1Cl)N | 178.62 | Positive | 7.8 |  | (6) |
|  | 1199-46-8 | CC(C)(C)C=1C=CC(=C(C=1)N)O | 165.23 | Negative | -15.6 |  | (5) |
|  | 1204-41-7 | CC1=CC=CC=C1C2=CC=C(C=C2)N | 183.25 | Positive | -11.2 |  | (18) |
|  | 1455-77-2 | C1(N)=NC(N)=NN1 | 99.09 | Positive | -46.4 | Ring Opening | https://pubchem.ncbi.nlm.nih.gov/bioassay/1259407#sid=363901695 |
|  | 1477-42-5 | CC=1C=CC=C2C=1N=C(N)S2 | 164.23 | Negative | -8.3 |  | https://echa.europa.eu/nl/registration-dossier/-/registered-dossier/24545/7/7/2 |
|  | 1520-21-4 | C=CC1=CC=C(C=C1)N | 119.16 | Positive | -10.0 |  | https://pubchem.ncbi.nlm.nih.gov/bioassay/1259407#sid=363902276 |
|  | 1535-75-7 | C1=CC=C(C(=C1)N)OC(F)(F)F | 177.12 | Negative | -0.3 |  | (5) |
|  | 1603-40-3 | CC=1C=CC=NC=1N | 108.14 | Negative | 4.7 |  | (6) |
|  | 1603-91-4 | CC1=CSC(=N1)N | 114.17 | Negative | -11.0 | Ring Opening | (5) |
|  | 1606-67-3 | C1=CC=2C=CC=3C=CC(=C4C=CC(=C1)C=2C=34)N | 217.27 | Positive | -29.0 |  | (12) |
|  | 1622-57-7 | CN2C1=CC=CC=C1N=C2N | 147.18 | Positive | -15.9 |  | (16) |
|  | 1684-40-8 | C1=CC=C3C(=C1)C(=C2CCCCC2=N3)N | 198.26 | Positive | -5.8 |  | https://tools.niehs.nih.gov//cebs3/ntpViews/?activeTab=detail&studyNumber=423580 |
|  | 1709-39-3 | CCN(CC)S(C1=CC=C(C=C1)N)(=O)=O | 228.31 | Negative | 12.6 |  | (19) |
|  | 1709-52-0 | CNS(C1=CC=C(C=C1)N)(=O)=O | 186.23 | Negative | 14.9 |  | (19) |
|  | 1732-23-6 | C1=CC=2C=CC=3C=C(C=C4C=CC(=C1)C=2C=34)N | 217.27 | Positive | -11.7 |  | (14) |
|  | 1744-22-5 | C1=CC2=C(C=C1OC(F)(F)F)SC(N)=N2 | 234.20 | Negative | -5.6 |  | https://labels.fda.gov/getProprietaryName.cfm?searchfield=Riluzole |
|  | 1805-02-3 | CN2C=1C=CC(=CC=1N=C2N)OC | 177.20 | Positive | -18.8 |  | (16) |
|  | 1818-71-9 | \| 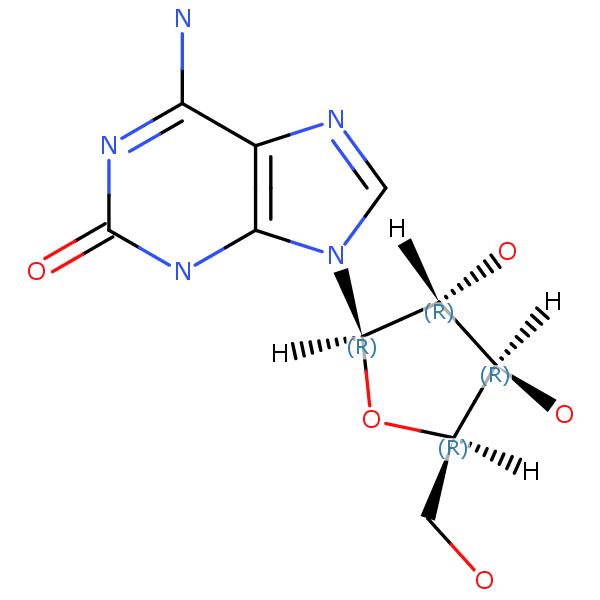C2=NC1=C(NC(=O)N=C1N2C3C(C(C(O3)CO)O)O)N \| \| --- \| | 283.24 | Negative | -3.4 | Ring Opening | (20) |
|  | 1892-54-2 | C=1C=CC2=C(C=1)C=CC=3C2=CC(=CC=3)N | 193.24 | Positive | -16.0 |  | (14) |
|  | 1904-98-9 | C=2NC1=C(N)N=C(N)N=C1N=2 | 150.14 | Positive | -1.7 | Two or more amines detected. The first site is used. | https://echa.europa.eu/nl/registration-dossier/-/registered-dossier/17706/7/7/2 |
|  | 2010-06-2 | C1=CC=C(C=C1)C2=CSC(=N2)N | 176.24 | Positive | -13.8 |  | http://tools.niehs.nih.gov/cebs3/ntpViews/?activeTab=detail&studyNumber=539422 |
|  | 2148-56-3 | C1=CC(=C(C(=C1)N)F)F | 129.11 | Negative | 9.0 |  | (5) |
|  | 2185-92-4 | C1=CC=C(C=C1)C2=CC=CC=C2N | 169.22 | Positive | -10.1 |  | https://tools.niehs.nih.gov/cebs3/ntpViews/?activeTab=detail&studyNumber=799083 |
|  | 2243-47-2 | C1=CC=C(C=C1)C=2C=CC=C(C=2)N | 169.22 | Positive | -3.0 |  | (14) |
|  | 2393-18-2 | C=1C=C(C=CC=1C=CC(O)=O)N | 163.17 | Negative | 0.1 |  | (21) |
|  | 2494-89-5 | C=1C=C(C=CC=1N)S(CCOS(O)(=O)=O)(=O)=O | 281.31 | Negative | 22.8 |  | https://echa.europa.eu/nl/registration-dossier/-/registered-dossier/11342/7/7/2 |
|  | 2642-98-0 | C1=CC=C3C(=C1)C=CC=4C2=CC=CC=C2C(=CC3=4)N | 243.30 | Positive | -25.8 |  | (22) |
|  | 2687-41-4 | CC(NC1=CC=C(C=C1)OC2=CC=C(C=C2)N)=O | 242.27 | Positive | -19.8 |  | (9) |
|  | 2693-46-1 | C1=CC=C2C(=C1)C=3C=CC=C4C(=CC=C2C=34)N | 217.27 | Positive | -20.3 |  | (14) |
|  | 2693-57-4 | C1(=C(C(=C(F)N=C1F)F)N)Cl | 182.53 | Negative | 36.9 |  | (6) |
|  | 2835-68-9 | C=1C=C(C=CC=1C(N)=O)N | 136.15 | Negative | 7.0 |  | https://echa.europa.eu/registration-dossier/-/registered-dossier/11042/7/7/2 |
|  | 2835-77-0 | C1=CC=C(C=C1)C(C2=CC=CC=C2N)=O | 197.23 | Negative | 2.5 |  | (5) |
|  | \| 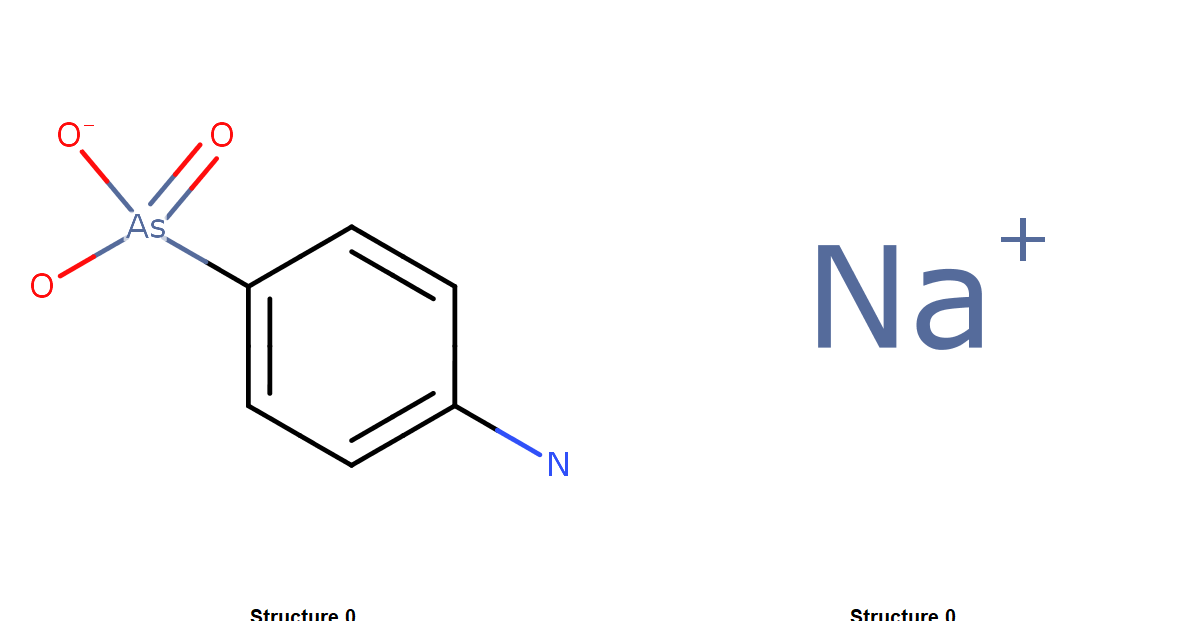2835-78-1 \| \| --- \| | C1=CC=C(C=C1)C(C=2C=CC=C(C=2)N)=O | 197.23 | Negative | 1.3 |  | (5) |
|  | 2835-95-2 | CC=1C=CC(=CC=1O)N | 123.15 | Negative | -4.0 |  | https://echa.europa.eu/nl/registration-dossier/-/registered-dossier/24086/7/7/2/?documentUUID=d63c8aa0-9187-45a8-b2d9-55d036ced352 |
|  | 2835-98-5 | CC=1C=CC(=C(C=1)O)N | 123.15 | Positive | -17.5 |  | (23) |
|  | 2876-22-4 | C=1C=CC=3C(C=1)=NC2=CC=CC(=C2N=3)N | 195.22 | Positive | -12.9 |  | (24) |
|  | 2876-23-5 | C1=CC=C3C(=C1)N=C2C=CC(=CC2=N3)N | 195.22 | Positive | -4.9 |  | (24) |
|  | 2922-83-0 | 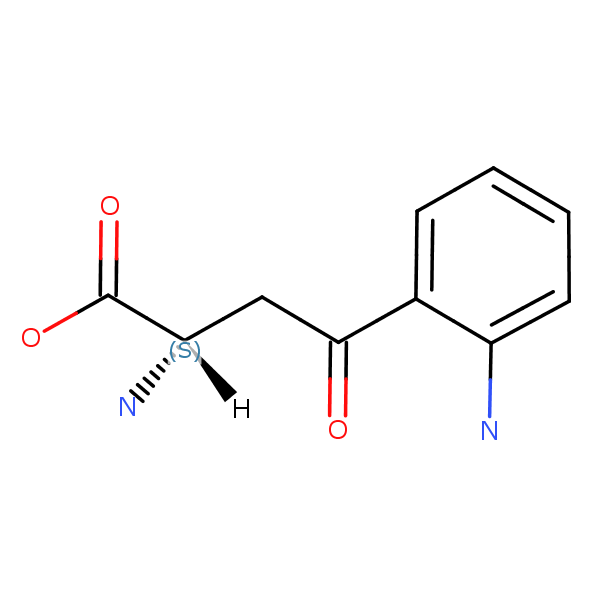   \| C1=CC=C(C(=C1)C(CC(C(O)=O)N)=O)N \| \| --- \| | 208.21 | Negative | 7.0 |  | https://pubchem.ncbi.nlm.nih.gov/bioassay/1259407#sid=363902375 |
|  | 2941-78-8 | C1=CC(=C(C(=C1)N)C(O)=O)Cl | 171.58 | Negative | 13.4 |  | (6) |
|  | \| 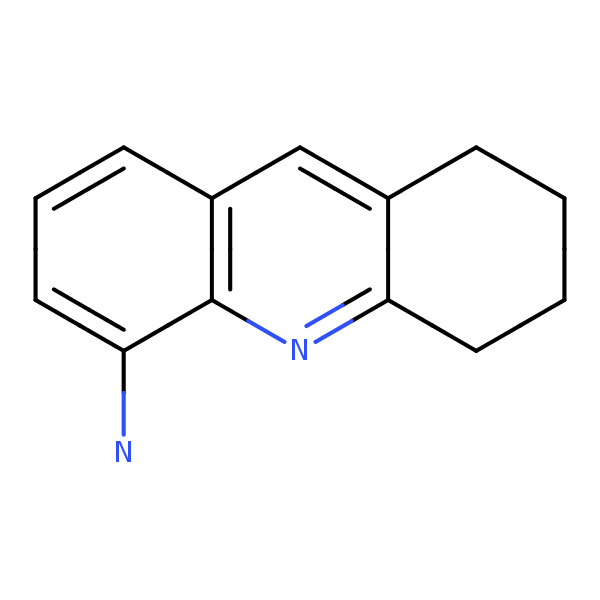3198-41-2 \| \| --- \| | C1CCC=3C(C1)=CC=2C=CC=C(C=2N=3)N | 198.26 | Positive | -18.2 |  | https://pubchem.ncbi.nlm.nih.gov/bioassay/1259407#sid=363900807 |
|  | 3366-61-8 | CC(NC1=CC=C(C=C1)C2=CC=C(C=C2)N)=O | 226.27 | Positive | -20.8 |  | https://pubchem.ncbi.nlm.nih.gov/bioassay/1259407#sid=363903116 |
|  | 3366-65-2 | C1=CC=C3C(=C1)C=CC2=CC(=CC=C23)N | 193.24 | Positive | -13.2 |  | (14) |
|  | 3544-23-8 | COC=1C=C(C=CC=1N)N=NC2=CC=CC=C2 | 227.26 | Positive | -22.1 |  | (25) |
|  | 3577-63-7 | C1=CC(=C(C=C1S(O)(=O)=O)C(O)=O)N | 217.20 | Negative | 33.5 |  | v |
|  | 4005-51-0 | C1=NN=C(S1)N | 101.13 | Negative | 1.3 |  | (5) |
|  | 4097-22-7 | C3CC(N2C=NC1=C(N)N=CN=C12)OC3CO | 235.24 | Negative | 7.0 |  | https://pubchem.ncbi.nlm.nih.gov/bioassay/1259407#sid=363898378 |
|  | 4106-66-5 | C1=CC=C3C(=C1)C=2C=CC(=CC=2O3)N | 183.21 | Positive | -9.9 |  | (26) |
|  | 4122-04-7 | C1=NC=NC(N)=N1 | 96.09 | Negative | 64.4 |  | (5) |
|  | 4176-53-8 | C1=CC=C3C(=C1)C=CC2=C3C=CC=C2N | 193.24 | Positive | -18.6 |  | (14) |
|  | 4261-14-7 | C1=CC=C(C=C1)CN3C=NC2=C(N)N=CN=C23 | 225.25 | Positive | 3.6 |  | (13) |
|  | 4261-16-9 | C1=CC=C(C=C1)CN3C=NC2=C(N)N(=CN=C23)=O | 241.25 | Positive | -10.3 |  | (13) |
|  | 4309-66-4 | C1=CC=C(C=C1)C=CC2=CC=C(C=C2)N | 195.26 | Positive | -18.9 |  | (18) |
|  | 4368-56-3 | C1CCC(CC1)NC2=CC(=C(C4=C2C(C3=CC=CC=C3C4=O)=O)N)S(O)(=O)=O | 400.45 | Negative | -15.7 |  | https://echa.europa.eu/nl/registration-dossier/-/registered-dossier/20754/7/7/2 |
|  | 4389-45-1 | CC=1C=CC=C(C=1N)C(O)=O | 151.16 | Positive | 3.7 |  | (6) |
|  | 4569-77-1 | C1=CC=C3C(=C1)N=C2C=C(C(=CC2=N3)O)N | 211.22 | Positive | -9.9 |  | https://pubchem.ncbi.nlm.nih.gov/bioassay/1259407#sid=363904771 |
|  | 5049-61-6 | C=1C=NC(=CN=1)N | 95.10 | Negative | 18.0 |  | (5) |
|  | 5202-89-1 | COC(=O)C1=C(C=CC(=C1)Cl)N | 185.61 | Negative | 7.5 |  | (6) |
|  | 5329-15-7 | CC(NC=1C=CC(=CC=1OC)N)=O | 180.20 | Positive | -23.6 |  | https://pubchem.ncbi.nlm.nih.gov/bioassay/1259407#sid=363899931 |
|  | 5369-19-7 | CC(C)(C)C=1C=CC=C(C=1)N | 149.23 | Negative | -3.7 |  | (5) |
|  | 5372-81-6 | COC(C=1C=CC(=C(C=1)N)C(=O)OC)=O | 209.20 | Negative | 10.9 |  | https://echa.europa.eu/nl/registration-dossier/-/registered-dossier/10651/7/7/2 |
|  | 5407-87-4 | CC1=CC(C)=NC(=C1)N | 122.17 | Negative | 8.2 |  | (6) |
|  | 5418-93-9 | C1=CC2=C(C=C1Cl)NC(N)=N2 | 167.60 | Negative | -9.9 |  | (16) |
|  | 5443-31-2 | CC2=CC(=C1C=C(C=CC1=N2)N)N | 173.21 | Positive | -20.7 | Two or more amines detected. The first site is used. | (6) |
|  | 5464-79-9 | COC=1C=CC=C2C=1N=C(N)S2 | 180.23 | Positive | -10.7 |  | http://tools.niehs.nih.gov/cebs3/ntpViews/?activeTab=detail&studyNumber=178190 |
|  | 5623-11-0 | CC(NC1=CC=CC(=C1C(O)=O)N)=O | 194.19 | Positive | 5.5 |  | (27) |
|  | 5794-88-7 | C1=CC(=C(C=C1Br)C(O)=O)N | 216.03 | Positive | 13.5 |  | (6) |
|  | 5869-25-0 | C1=CC=2C=CC=C3C=4C=C(C=CC=4C(=C1)C=23)N | 217.27 | Positive | -17.8 |  | (14) |
|  | 5900-58-3 | COC(C=1C=CC(=CC=1N)Cl)=O | 185.61 | Negative | 12.9 |  | (6) |
|  | 6201-87-2 | C1=C(C=C(C(=C1C(O)=O)O)S(O)(=O)=O)N | 233.20 | Negative | 4.0 |  | http://tools.niehs.nih.gov/cebs3/ntpViews/?activeTab=detail&studyNumber=181228 |
|  | 6219-89-2 | CC1=CC(=CC=C1N)NC2=CC=C(C=C2)O | 214.26 | Positive | -36.2 |  | http://tools.niehs.nih.gov/cebs3/ntpViews/?activeTab=detail&studyNumber=027403 |
|  | 6232-91-3 | COC=1C=CC2=C(C=1)N=C(N)N2 | 163.18 | Negative | -14.7 |  | (16) |
|  | \| 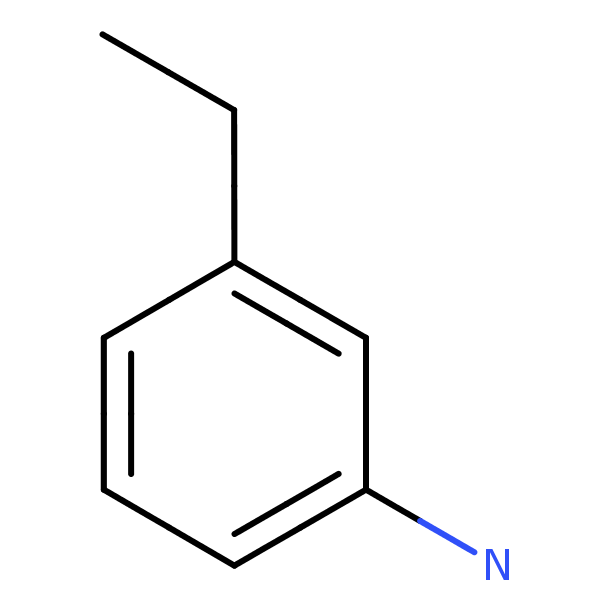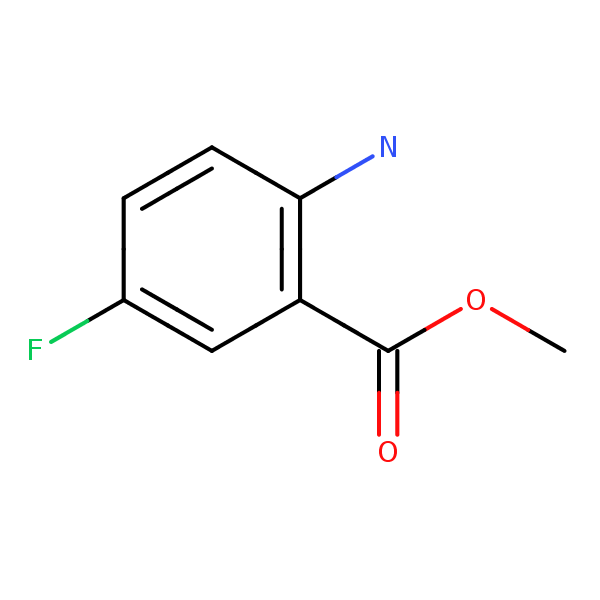6285-68-3 \| \| --- \| | CC=1C=CC2=C(C=1)NC(N)=N2 | 147.18 | Positive | -16.0 |  | (16) |
|  | 6344-63-4 | C1=CC=C3C(=C1)CC2=C3C=CC=C2N | 181.23 | Positive | -6.7 |  | (14) |
|  | 6344-66-7 | C1=CC=C3C(=C1)CC=2C=CC(=CC=23)N | 181.23 | Positive | -9.1 |  | (14) |
|  | 6375-16-2 | CC=1C=CC(=CC=1N)NC(C)=O | 164.20 | Positive | -7.0 |  | (28) |
|  | 6377-12-4 | C=1C=CC2=C(C=1)C=3C(N2)=CC=C(C=3)N | 182.22 | Positive | -27.3 |  | (29) |
|  | 6575-11-7 | C1=CC(=C(C#N)C(=C1)N)Cl | 152.58 | Negative | 14.1 |  | (5) |
|  | 7083-63-8 | C1=CC=C3C(=C1)CC=2C=CC=C(C=23)N | 181.23 | Positive | -16.0 |  | (14) |
|  | 7132-67-4 | C1=CC=C3C(=C1)C(=C2C=C(C=CC2=N3)O)N | 210.23 | Positive | -23.1 |  | (30) |
|  | 7252-84-8 | COC=1C=CC(N)=NN=1 | 125.13 | Negative | -2.7 |  | (5) |
|  | 7269-58-1 | C(C3C(C(C(N2C=NC1=C(N=C(N)N=C12)NO)O3)O)O)O | 298.26 | Positive | 8.0 |  |  |
|  | 7493-63-2 | C=CCOC(C1=CC=CC=C1N)=O | 177.20 | Negative | 6.3 |  | https://tools.niehs.nih.gov/cebs3/ntpViews/?activeTab=detail&studyNumber=342529 |
|  | 7570-36-7 | C(=CC1=CC=C(C=C1)N)C2=CC=C(C=C2)Cl | 229.71 | Positive | -16.9 |  | (18) |
|  | 7570-37-8 | COC=1C=CC(=CC=1)C=CC=2C=CC(=CC=2)N | 225.29 | Positive | -23.1 |  | (31) |
|  | 7756-96-9 | CCCCOC(C1=CC=CC=C1N)=O | 193.24 | Negative | 6.3 |  | https://tools.niehs.nih.gov/cebs3/ntpViews/?activeTab=detail&studyNumber=729369 |
|  | 7779-16-0 | C1CCC(CC1)OC(C2=CC=CC=C2N)=O | 219.28 | Negative | 5.3 |  | https://tools.niehs.nih.gov/cebs3/ntpViews/?activeTab=detail&studyNumber=828926 |
|  | 7779-77-3 | CC(C)COC(C1=CC=CC=C1N)=O | 193.24 | Negative | 6.2 |  | https://tools.niehs.nih.gov/cebs3/ntpViews/?activeTab=detail&studyNumber=521900 |
|  | 10312-55-7 | C1=CC(=C(C=C1C(O)=O)N)C(O)=O | 181.15 | Negative | 15.6 |  | (6) |
|  | 13024-49-2 | C1=CC=C(C=C1)CCC2=CC=C(C=C2)N | 197.28 | Positive | -7.1 |  | (18) |
|  | \| 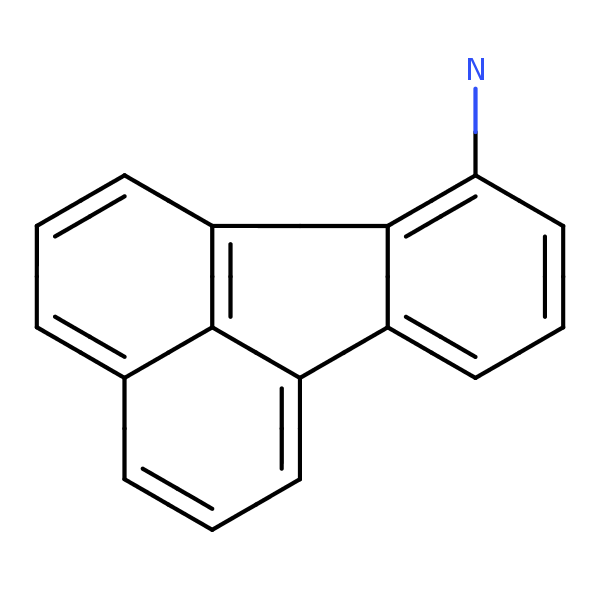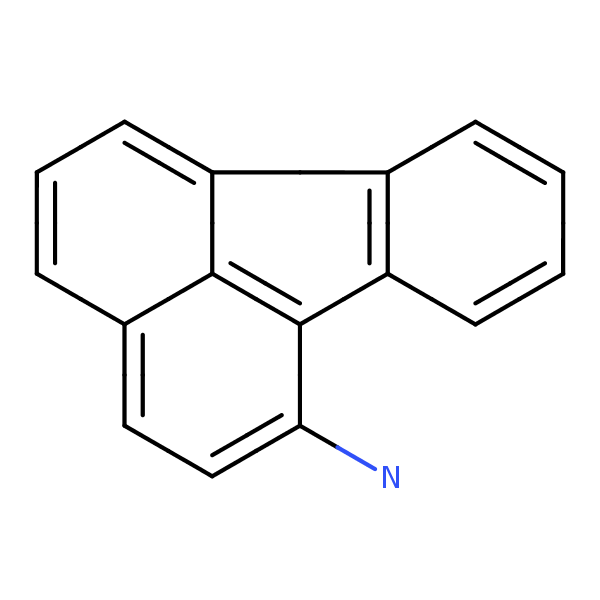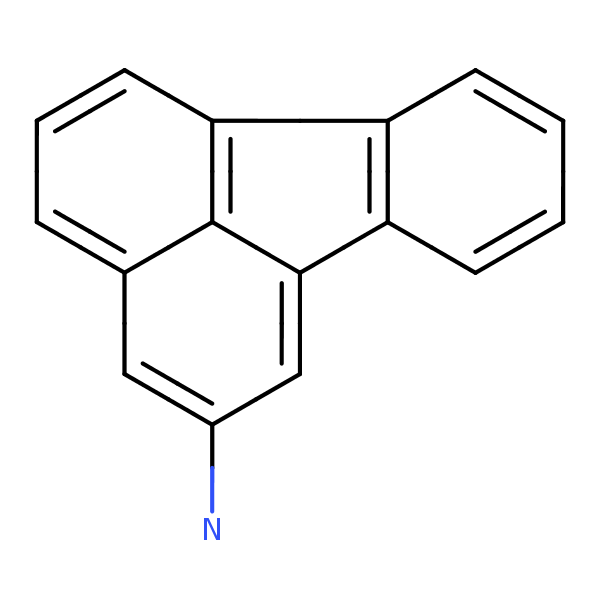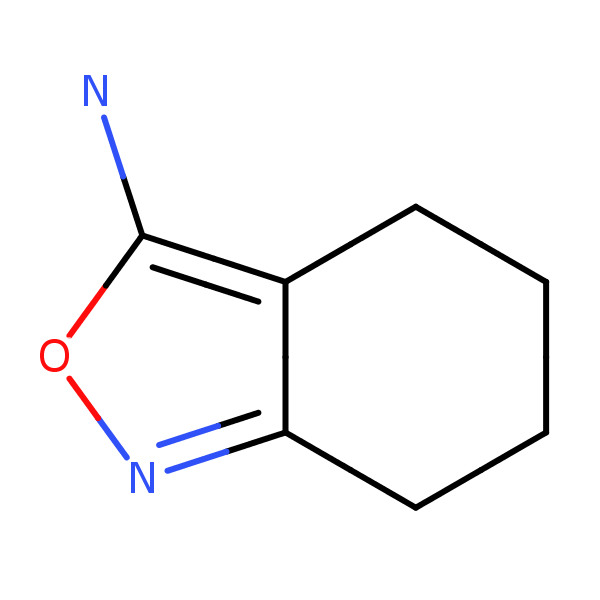13054-47-2 \| \| --- \| | C1CCC=2C(C1)=C(N)ON=2 | 138.17 | Positive | -39.0 | Ring Opening | (5) |
|  | 13177-25-8 | C=1C=CC3=C(C=1)C2=CC=CC=4C2=C3C(=CC=4)N | 217.27 | Positive | -16.5 |  | (14) |
|  | 13177-26-9 | C1=CC=C2C(=C1)C4=CC=CC3=CC(=CC2=C34)N | 217.27 | Positive | -12.9 |  | (14) |
|  | 13177-27-0 | C2=CC=1C=CC=C4C=1C(=C2)C=3C=CC=C(C=34)N | 217.27 | Positive | -17.6 |  | (14) |
|  | 13306-69-9 | CC1=CC(=CC=C1N)OP(OC)(OC)=S | 247.25 | Positive | -8.0 |  | (32) |
|  | 13394-86-0 | CC1=CC=CC=C1C2=CC=C(C(C)=C2)N | 197.28 | Positive | -14.9 |  | (1) |
|  | 13629-82-8 | CC=1C=CC=C(C=1)C2=CC=C(C(C)=C2)N | 197.28 | Positive | -16.8 |  | (1) |
|  | \| 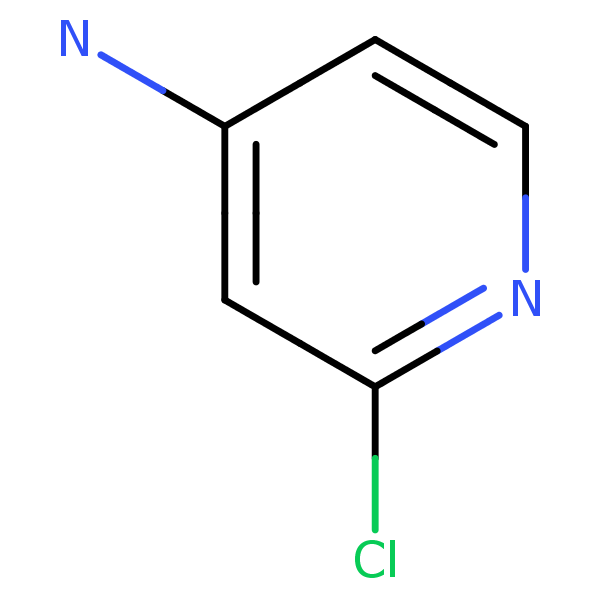14432-12-3 \| \| --- \| | C1=CN=C(C=C1N)Cl | 128.56 | Negative | 27.1 |  | (5) |
|  | 14438-32-5 | CC1=CC(C)=C(C(=C1)C(O)=O)N | 165.19 | Positive | -2.3 |  | (6) |
|  | 14861-17-7 | C1=CC(=C(C=C1Cl)Cl)OC2=CC=C(C=C2)N | 254.11 | Positive | -14.2 |  | (33) |
|  | 16452-01-0 | CC=1C=CC(=CC=1OC)N | 137.18 | Positive | -6.9 |  | https://tools.niehs.nih.gov/cebs3/ntpViews/?activeTab=detail&studyNumber=004480 |
|  | 17026-81-2 | CCOC=1C=CC(=CC=1N)NC(C)=O | 194.23 | Positive | -15.6 |  | https://tools.niehs.nih.gov/cebs3/ntpViews/?activeTab=detail&studyNumber=851003 |
|  | 17075-03-5 | C1=CC=2C=CC=3C=CC=C4C(=CC(=C1)C=2C=34)N | 217.27 | Positive | -22.5 |  | (14) |
|  | 17418-58-5 | C1=CC=C(C=C1)OC=2C=C(C4=C(C=2N)C(C3=CC=CC=C3C4=O)=O)O | 331.32 | Positive | -16.0 |  | https://echa.europa.eu/nl/registration-dossier/-/registered-dossier/16393/7/7/2 |
|  | 17423-48-2 | C1=CC=C3C(=C1)C=CC=2C=CC=C(C=23)N | 193.24 | Negative | -17.0 |  | (14) |
|  | 18437-58-6 | CC1=CC(=CC=N1)N | 108.14 | Negative | 20.0 |  | (5) |
|  | 19798-81-3 | C1=CC(Br)=NC(=C1)N | 173.01 | Negative | 16.8 |  | (6) |
|  | 19952-47-7 | C1=CC(=C2C(=C1)SC(N)=N2)Cl | 184.65 | Negative | -3.7 |  | http://tools.niehs.nih.gov/cebs3/ntpViews/?activeTab=detail&studyNumber=147420 |
|  | 20744-39-2 | C1=CN=NC=C1N | 95.10 | Negative | 29.5 |  | (5) |
|  | 21802-46-0 | C1=CC=C(C=C1)C(C2=CC=CC=C2)(C3=CC=CC=C3)N5C=NC4=C(N)N=CN=C45 | 377.44 | Positive | -3.3 |  | (13) |
|  | 23045-12-7 | CC=1C=CC=3C(C=1)=C(C2=CC=CC=C2N=3)N | 208.26 | Positive | -20.0 |  | (34) |
|  | 23250-39-7 | C1=CC=C3C(=C1)C(=C2C=C(C=CC2=N3)Cl)N | 228.68 | Positive | -14.6 |  | (34) |
|  | 24340-76-9 | CC1=NSC(=C1)N | 114.17 | Negative | 0.6 |  | (5) |
|  | 24367-94-0 | CC(NC1=CC=C(C=C1)CC2=CC=C(C=C2)N)=O | 240.30 | Negative | -10.1 |  | (9) |
|  | 24544-04-5 | CC(C)C=1C=CC=C(C(C)C)C=1N | 177.29 | Negative | -12.6 |  | https://echa.europa.eu/nl/registration-dossier/-/registered-dossier/10001/7/7/2 |
|  | 24549-06-2 | CCC1=CC=CC(C)=C1N | 135.21 | Negative | -10.3 |  | https://echa.europa.eu/nl/registration-dossier/-/registered-dossier/12644/7/7/2 |
|  | 26175-68-8 | CN(C)S(C=1C=C(C(=CC=1Cl)N)Cl)(=O)=O | 269.15 | Negative | 17.3 |  | https://echa.europa.eu/nl/registration-dossier/-/registered-dossier/7596/7/7/2 |
|  | 26049-71-8 | C=1C=C(C=CC=1C2=CSC(=N2)NN)N | 206.27 | Positive | -13.1 |  | (25) |
|  | 26306-61-6 | C=1C=C(C=CC=1N)OC2=C(C=C(C=C2Cl)Cl)Cl | 288.56 | Positive | -14.3 |  | (35) |
|  | 26148-68-5 | C1=CC=C3C(=C1)C=2C=CC(N)=NC=2N3 | 183.21 | Positive | -8.7 |  | (16) |
|  | 28443-50-7 | C1=CC(=C(C=C1Cl)O)N | 143.57 | Negative | -10.9 |  | https://echa.europa.eu/nl/registration-dossier/-/registered-dossier/12346/7/7/2 |
|  | 29927-08-0 | CC1=CC2=C(C=C1C)SC(N)=N2 | 178.25 | Positive | -11.2 |  | https://tools.niehs.nih.gov/cebs3/ntpViews/?activeTab=detail&studyNumber=546581 |
|  | 30489-67-9 | CN2C1=CC3=CC=CC=C3C=C1N=C2N | 197.24 | Positive | -14.0 |  | (36) |
|  | 31272-21-6 | CC=2C(C(C1=CC=CC=C1F)=O)=C(N)N(C)N=2 | 233.24 | Positive | -8.5 |  | (37) |
|  | 24526-64-5 | CN2CC(C1=CC=CC=C1)C=3C=CC=C(C=3C2)N | 238.33 | Negative | -9.1 |  | (38) |
|  | 33421-40-8 | C1=CC=C(C=C1)C=2C=CC(N)=NC=2 | 170.21 | Positive | -4.0 |  | (6) |
|  | 36193-75-6 | \| 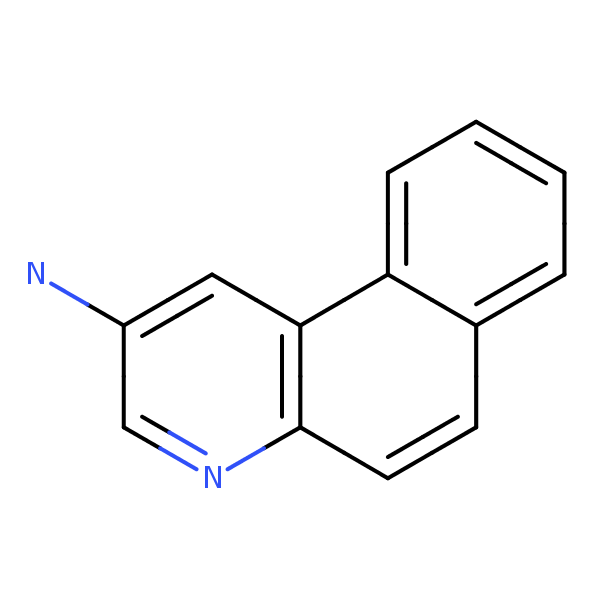C1=CC=C3C(=C1)C=CC2=C3C=C(C=N2)N \| \| --- \| | 194.23 | Positive | -12.3 |  | (39) |
|  | 39860-12-3 | CC=1C=CC2=C(C=1)N=C(N)N2C | 161.20 | Positive | -17.7 |  | (16) |
|  | 40483-47-4 | CC1=NC(N)=NO1 | 99.09 | Negative | -28.5 | Ring Opening | (5) |
|  | 41200-96-8 | CC(C)OC=1C=C(C(=CC=1Cl)Cl)N | 220.10 | Positive | 0.0 |  | https://echa.europa.eu/nl/registration-dossier/-/registered-dossier/12596/7/7/2 |
|  | 42016-93-3 | C1=CC(=C(C=C1I)Cl)N | 253.47 | Positive | 5.7 |  | https://pubchem.ncbi.nlm.nih.gov/bioassay/1259407#sid=363907384 |
|  | 42753-71-9 | \| 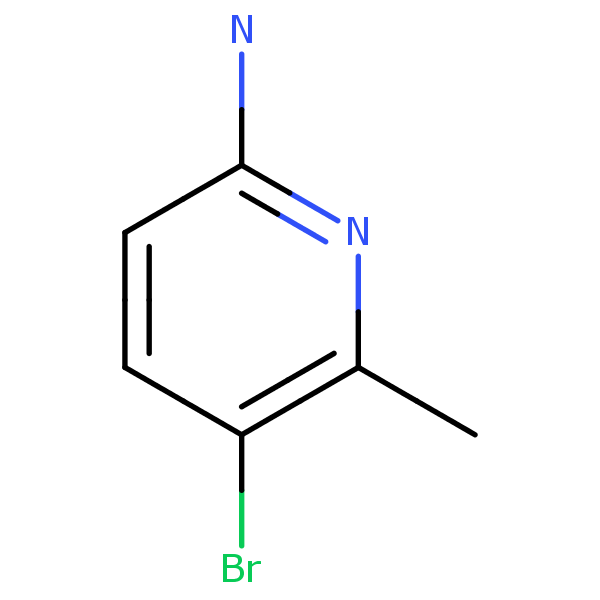CC1=C(C=CC(N)=N1)Br \| \| --- \| | 360.05 | Positive | 13.0 |  | (6) |
|  | 49701-24-8 | CNS(C=1C=C(C(=CC=1OC)N)OC)(=O)=O | 246.28 | Negative | -3.0 |  | https://echa.europa.eu/nl/registration-dossier/-/registered-dossier/5400/7/7/2 |
|  | 50419-58-4 | CC=1C=CC(=C(C=1C)N)C(O)=O | 165.19 | Negative | 2.3 |  | (6) |
|  | 51276-85-8 | C1=CC2=C(C=C1O)N=C(N)N2 | 149.15 | Positive | -12.2 |  | (16) |
|  | 51357-79-0 | C1=CC=2C(C=C1Cl)=C(N)SN=2 | 184.65 | Positive | -17.4 |  | (40) |
|  | 52727-57-8 | COC(C=1C=C(C=CC=1N)Br)=O | 230.06 | Negative | 11.0 |  | (6) |
|  | 53473-85-1 | C=1C=C2C(=CC=1N)C=NS2 | 150.20 | Positive | -9.2 |  | (41) |
|  | 54150-69-5 | COC=1C=C(C(=CC=1)N)OC | 153.18 | Positive | -27.6 |  | https://tools.niehs.nih.gov/cebs3/ntpViews/?activeTab=detail&studyNumber=792648 |
|  | 54381-16-7 | C=1C=C(C=CC=1N)N(CCO)CCO | 196.25 | Negative | -32.4 |  | https://echa.europa.eu/nl/registration-dossier/-/registered-dossier/23320/7/7/2 |
|  | 54779-53-2 | CC=1C4=CN=CC=C4C(C)=C3C=1C=2C=C(C=CC=2N3)N | 261.32 | Positive | -28.0 |  | https://pubchem.ncbi.nlm.nih.gov/bioassay/1259407#sid=363904431 |
|  | 54810-82-1 | CC=1C=C(C=C(C)C=1N)C2=CC=CC=C2 | 197.28 | Positive | -19.3 |  | (42) |
|  | 56341-08-3 | CC(C)(C)NCC(C1=CC(=C(C(=C1)Cl)N)C(F)(F)F)O | 310.74 | Negative | 8.0 |  | https://pubchem.ncbi.nlm.nih.gov/bioassay/1259407#sid=363903238 |
|  | 58658-11-0 | C=1C=CC=3C(C=1)=C(C2=CC=CC=C2N=3)NC4=CC=C(C=C4)N | 285.34 | Positive | -31.1 |  | (43) |
|  | 59683-60-2 | COC=1C=C(C=CC=1N)OC=2C=CC(=CC=2Cl)Cl | 284.14 | Positive | -25.8 |  | (33) |
|  | 60877-79-4 | C1=C(C=C(C=2C1=C(N)SN=2)Cl)Cl | 219.09 | Positive | -16.3 |  | (44) |
|  | 61166-04-9 | CN2C3=CC=CC=C3C=1C=C(C=CC=12)N | 196.25 | Positive | -30.2 |  | (45) |
|  | 62450-06-0 | CC=1C(=NC(=C2C=1NC=3C=CC=CC2=3)C)N | 211.26 | Positive | -16.2 |  | (46) |
|  | 62450-07-1 | CC2=C3C1=CC=CC=C1NC3=CC(N)=N2 | 197.24 | Positive | -11.6 |  | (46) |
|  | 63467-52-7 | C=1C=C(C=CC=1CC(=O)[O-])N | 150.15 | Positive | -146.2 |  | https://pubchem.ncbi.nlm.nih.gov/bioassay/1259407#sid=363901107 |
|  | 63655-40-3 | C1=CC2=C(C=C1C#N)NC(N)=N2 | 158.16 | Positive | -2.6 |  | (16) |
|  | 64099-27-0 | COC=2C=1C=CC=C(C=1SN=2)N | 180.23 | Positive | -18.9 |  | (47) |
|  | 64099-28-1 | COC=2C=1C=C(C=CC=1SN=2)N | 180.23 | Positive | -15.9 |  | (47) |
|  | 67199-66-0 | C1=CC=C3C(=C1)C2=NC4=CC=C(C=C4CN2C3=O)N | 249.27 | Positive | -21.5 |  | (48) |
|  | 67410-22-4 | C1=CC=C3C(=C1)C=CC=2C=CC(N)=NC=23 | 194.23 | Positive | -5.1 |  | https://pubchem.ncbi.nlm.nih.gov/bioassay/1259408#sid=363897780 |
|  | 67545-00-0 | CN1C=C(N)N=N1 | 98.11 | Negative | -12.9 |  | (5) |
|  | 67730-10-3 | C=1C=CN3C(C=1)=NC=2C=CC(N)=NC=23 | 184.20 | Positive | -21.1 |  | (16) |
|  | 67730-11-4 | CC1=CC=CN3C1=NC=2C=CC(N)=NC=23 | 198.22 | Positive | -22.7 |  | (16) |
|  | 68006-83-7 | CC3=CC=2C1=CC=CC=C1NC=2N=C3N | 197.24 | Positive | -12.1 |  | (16) |
|  | 68302-57-8 | CC(C)C=1C=CC3=C(C=1)C(C2=CC(=C(N)N=C2O3)C(O)=O)=O | 298.29 | Negative | 37.1 |  | https://www.accessdata.fda.gov/drugsatfda_docs/nda/2004/021727s000_OraDiscTOC.cfm |
|  | 71711-58-5 | CC2=C(C)C4=C(C1=CC=CC=C1N=C4C3=CC=CC=C23)N | 272.34 | Positive | -25.9 |  | (49) |
|  | 74420-00-1 | C1C=NC=2C1=C(C=CN=2)N | 133.15 | Negative | 19.6 |  | (6) |
|  | 74441-06-8 | C=1C=C(C=CC=1C(NC2=CC=C(C=C2)C(N)=O)=O)N | 255.27 | Negative | 9.7 |  | https://echa.europa.eu/nl/registration-dossier/-/registered-dossier/11122/7/7/2 |
|  | 76180-96-6 | CN3C=2C=CC1=C(C=CC=N1)C=2N=C3N | 198.22 | Positive | -22.9 |  | (50) |
|  | 77094-11-2 | CC2=CC1=C(C=CC=N1)C3=C2N(C)C(N)=N3 | 212.25 | Positive | -23.6 |  | (50) |
|  | 77618-99-6 | CSC=1C=CC(N)=NC=1 | 140.21 | Positive | -13.4 |  | (6) |
|  | 77500-04-0 | CC=2C=NC=1C=CC3=C(C=1N=2)N=C(N)N3C | 213.24 | Positive | -21.5 |  | (50) |
|  | 78069-95-1 | C=1C=NC=C(C=1N2CCNCC2)N | 178.23 | Negative | -27.5 |  | (6) |
|  | 78411-56-0 | CC=3C=NC1=C(C=CC2=C1N=C(N)N2C)N=3 | 213.24 | Positive | -21.3 |  | (51) |
|  | 79642-27-6 | C1=CC=C3C(=C1)C2=C(C=CN=C2N)N3 | 183.21 | Positive | -11.4 |  | (52) |
|  | 83407-42-5 | CC2=CC1=C(C=CC=N1)C(=C2NC)N | 187.24 | Negative | -41.7 |  | (50) |
|  | 83506-93-8 | C=1C(=C(C=C(C=1F)F)N)C(O)=O | 173.12 | Negative | 18.0 |  | (5) |
|  | 88133-11-3 | CCOCCC2=C(C1=CC=CC=C1)N=C(N)N3C2=NC=N3 | 283.33 | Negative | 2.9 |  | (50) |
|  | \| 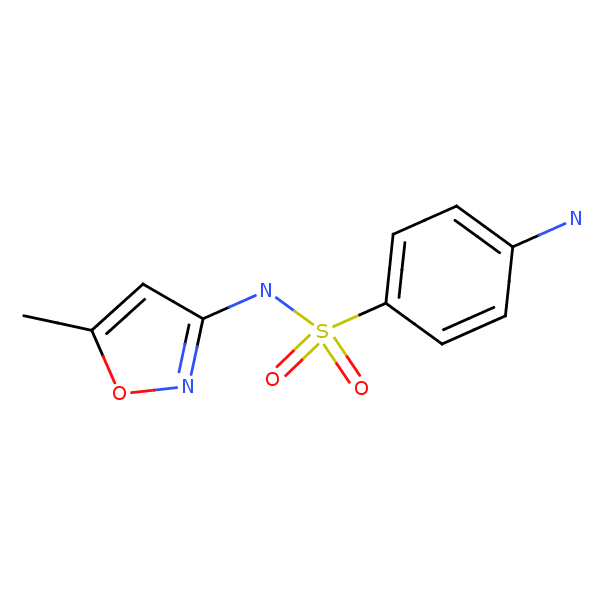89459-43-8 \| \| --- \| | CN(C)CCNC(C3=CC=CC2=C(C1=CC=CC=C1N=C23)N)=O | 308.38 | Positive | -10.5 |  | (53) |
|  | 89738-68-6 | C1CC1C=2C=CC(N)=NC=2 | 134.18 | Positive | 0.3 |  | (6) |
|  | 89795-79-9 | C=1C=C2C(=C(C=1)N)SN=C2 | 150.20 | Positive | -11.7 |  | (47) |
|  | 90223-04-4 | CN2C=1C=CC(=CC=1N=C2N)O | 163.18 | Negative | -16.3 |  | (16) |
|  | 90594-13-1 | C3=CC2=NC=1C=C(C=NC=1N2C=C3)N | 184.20 | Positive | -10.4 |  | (54) |
|  | 90597-22-1 | C2=CN(C1C=C(CO)C(C1O)O)C(N=C2N)=O | 239.23 | Negative | 50.6 |  | https://pubchem.ncbi.nlm.nih.gov/bioassay/1259407#sid=363899721 |
|  | 91575-28-9 | CC(NC=2C=CC(CC=1C=CC(=C(C=1)Cl)N)=CC=2Cl)=O | 309.19 | Positive | -6.5 |  | (55) |
|  | 92180-79-5 | CC=3C(C)=NC1=C(C=CC2=C1N=C(N)N2C)N=3 | 227.27 | Positive | -23.2 |  | (56) |
|  | 92758-43-5 | C1=CC=C2C(=C1)C=C3C=CC=4C=CC(=C5C=CC2=C3C=45)N | 267.32 | Positive | -33.9 |  | (57) |
|  | 93535-04-7 | C2=CC(C=CC1=CC=C(C=C1)N)=CC(=C2)Cl | 229.71 | Positive | -16.1 |  | (31) |
|  | 95186-19-9 | C1=CC=C(C=C1)C3=C(CCO)C2=NC=NN2C(N)=N3 | 255.28 | Negative | 4.7 |  | (58) |
|  | 95896-78-9 | CC1=CC3=C(C2=C1N(C)C(N)=N2)N=C(C)C=N3 | 227.27 | Positive | -22.1 |  | (56) |
|  | 96287-03-5 | CC1=CC=CN3C1=NC2=C(C)C(=CN=C23)N | 212.25 | Positive | -16.2 |  | (54) |
|  | 96799-03-0 | C=2C=C(C1=CC(N)=NN1)SC=2 | 165.22 | Negative | -11.6 |  | (5) |
|  | 97136-66-8 | CC1=CC=C(C=C1)C=CC2=CC=C(C=C2)N | 209.29 | Positive | -20.9 |  | (59) |
|  | 97389-17-8 | CC1=CC3=C(C2=C1N(C)C(N)=N2)N=CC(C)=N3 | 227.27 | Positive | -22.1 |  | (56) |
|  | 97919-22-7 | C1=CC(=C2C(=C1)N=C(C=N2)NS(C3=CC=C(C=C3)N)(=O)=O)Cl | 334.78 | Negative | 22.6 |  | https://pubchem.ncbi.nlm.nih.gov/bioassay/1259407#sid=363898639 |
|  | 98151-92-9 | C1=CC=C2C(=C1)C=C3C=CC5=C(C=CC=4C=CC2=C3C=45)N | 267.32 | Positive | -33.1 |  | (60) |
|  | 99523-86-1 | CC=1C=CC=2C(C=1)=C(N)SN=2 | 164.23 | Positive | -22.9 |  | (44) |
|  | \| 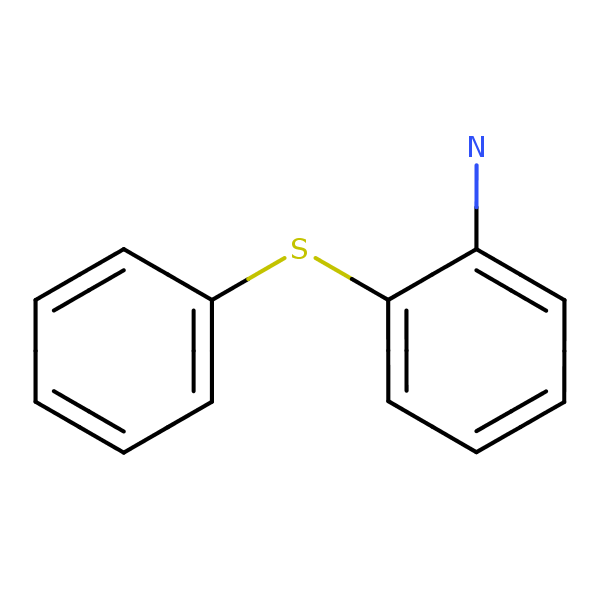99601-30-6 \| \| --- \| | CCN2C=1C=CC3=C(C=1N=C2N)N=CC(C)=N3 | 227.27 | Positive | -22.1 |  | (51) |
|  | 99601-31-7 | CCN2C=1C=CC3=C(C=1N=C2N)N=C(C)C=N3 | 227.27 | Positive | -22.2 |  | (51) |
|  | 99601-32-8 | CCN3C=2C(C)=CC1=C(C=CC=N1)C=2N=C3N | 226.28 | Positive | -24.5 |  | (51) |
|  | 99601-33-9 | CCN3C=2C=CC1=C(C=CC=N1)C=2N=C3N | 212.25 | Positive | -23.8 |  | (51) |
|  | 100114-58-7 | C1=CN=C(C=C1N)CO | 124.14 | Negative | 22.8 |  | (5) |
|  | 101821-43-6 | C1=CC2=C(C=C1C(=N)N)NC(=C2N)C(=N)N | 216.24 | Negative | -36.7 |  | (19) |
|  | 102408-25-3 | CN2C=1C=3C=CC=NC=3C=CC=1N=C2N | 198.22 | Positive | -21.9 |  | (16) |
|  | 103139-94-2 | CC1=CC3=C(C2=C1N=CC(C)=N2)N=C(N)N3C | 227.27 | Positive | -24.9 |  | (56) |
|  | 103748-25-0 | CN2C=1C=CC(=CC=1N=C2N)Cl | 181.62 | Positive | -12.0 |  | (16) |
|  | 105650-23-5 | CN2C=1C=C(C=NC=1N=C2N)C3=CC=CC=C3 | 224.26 | Positive | -15.3 |  | (61) |
|  | 108043-88-5 | CN3C=2C=CC1=C(C=CC(N1)=O)C=2N=C3N | 214.22 | Positive | -14.1 |  | (62) |
|  | 108618-28-6 | C2=CC(C=CC1=CC=C(C=C1)N)=CC(=C2)C#N | 220.27 | Positive | -13.3 |  | (31) |
|  | 108354-48-9 | CC1=CC3=C(C2=C1N(C)C(N)=N2)N=CC=N3 | 213.24 | Positive | -20.3 |  | (56) |
|  | 108618-29-7 | C(=CC1=CC=C(C=C1)N)C2=CC=C(C=C2)C#N | 220.27 | Positive | -12.7 |  | (31) |
|  | 110963-29-6 | C1=CC=C2C(=C1)C(=C(C(NN)=O)N2)N | 190.20 | Positive | -23.4 |  | (63) |
|  | 115084-47-4 | C1=CC(=C(C=C1CC=2C=CC(=C(C=2)Cl)NO)Cl)N | 283.15 | Positive | -5.0 |  | (52) |
|  | 115609-71-7 | CC1=CC3=C(C2=C1N=C(C)C=N2)N=C(N)N3C | 227.27 | Positive | -24.7 |  | (56) |
|  | 117760-93-7 | C1=CC=C3C(=C1)C=4C=CC2=C(C=CC(C2O)O)C=4C=C3N | 277.32 | Positive | -21.1 |  | (64) |
|  | 119260-67-2 | C1=CC(=C(C=C1CC=2C=CC(=C(C=2)Cl)N=O)Cl)N | 281.14 | Negative | -0.8 |  | (52) |
|  | 122349-91-1 | CC1=CC(C)=NC=2C=C(C(C)=CC1=2)N | 186.25 | Positive | -8.3 |  | (65) |
|  | 122365-34-8 | C1=CC=C(C=C1)C(C2=CC=CC=C2)N4C=NC3=C(N)N=CN=C34 | 301.35 | Positive | -0.5 |  | (13) |
|  | 122365-36-0 | C1=CC=C(C=C1)C(C2=CC=CC=C2)(C3=CC=CC=C3)N5C=NC4=C(N)N(=CN=C45)=O | 393.44 | Positive | -14.9 |  | (13) |
|  | 129117-54-0 | CC2=CC(=C(C)C=3C=1C=C(C=CC=1NC2=3)OC)N | 240.30 | Positive | -34.0 |  | (45) |
|  | \| 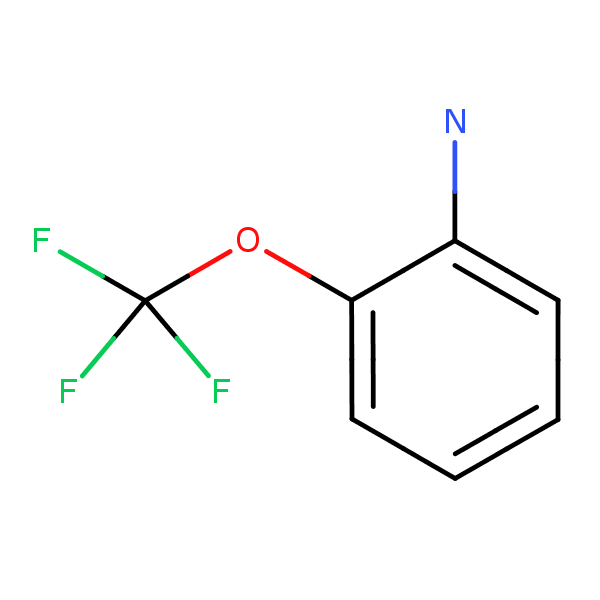130005-62-8 \| \| --- \| | CC2=CC(=C(C)C=3C=1C=C(C=CC=1NC2=3)O)N | 226.27 | Positive | -31.9 |  | (45) |
|  | 132674-71-6 | C1=CC=C3C(=C1)C(=C2C(CCCC2=O)=N3)N | 212.25 | Positive | 8.0 |  | (6) |
|  | 132898-07-8 | CC1=CC3=C(C2=C1N(C)C(N)=N2)N=C(C)C(C)=N3 | 241.29 | Positive | -23.7 |  | (56) |
|  | 135086-99-6 | C1=CC=C(C=C1)C(=NNC(C3=C(C2=CC=CC=C2N3)N)=O)[H] | 278.31 | Negative | -33.8 |  | (63) |
|  | 141691-41-0 | CN2C=1C=CC(=CC=1N=C2N)C#N | 172.19 | Positive | -7.2 |  | (16) |
|  | 141691-42-1 | CN2C=1C=C(C=CC=1N=C2N)C#N | 172.19 | Positive | -6.7 |  | (16) |
|  | 141890-75-7 | C3=CC1=C(C=CC2=C1N=C(N)S2)N=C3 | 201.25 | Positive | -12.4 |  | (66) |
|  | 146177-57-3 | CC1=CC3=C(C2=C1N=CC=N2)N=C(N)N3C | 213.24 | Positive | -23.2 |  | (56) |
|  | 146177-58-4 | CC1=CC3=C(C2=C1N=C(C)C(C)=N2)N=C(N)N3C | 241.29 | Positive | -26.6 |  | (56) |
|  | 146177-59-5 | CC1=C(C)C3=C(C2=C1N=CC=N2)N=C(N)N3C | 227.27 | Positive | -23.5 |  | (56) |
|  | 146177-60-8 | CC=2C=NC=1C(C)=C(C)C3=C(C=1N=2)N=C(N)N3C | 241.29 | Positive | -25.3 |  | (56) |
|  | 146177-61-9 | CC=2C=NC=1C3=C(C(=C(C=1N=2)C)C)N(C(=N3)N)C | 241.29 | Positive | -25.2 |  | (56) |
|  | 146177-62-0 | CC1=C(C)C3=C(C2=C1N=C(C)C(C)=N2)N=C(N)N3C | 255.32 | Positive | -26.8 |  | (56) |
|  | 146177-64-2 | CC1=CC4=C(C3=C1N=CC(C2=CC=CC=C2)=N3)N=C(N)N4C | 289.34 | Positive | -26.0 |  | (56) |
|  | 146177-65-3 | CC=1C=C4C(=C2N=CC(=NC=12)C=3C=CC=CC=3)N=C(N4C)N | 289.34 | Positive | -25.5 |  | (56) |
|  | 147293-15-0 | CN3C2=CC=C1C=NC=CC1=C2N=C3N | 198.22 | Positive | -22.3 |  | (67) |
|  | 147293-13-8 | CN3C=2C=CC=1C=CC=NC=1C=2N=C3N | 198.22 | Positive | -24.7 |  | (67) |
|  | 147293-14-9 | CN3C=2C=CC1=CC=NC=C1C=2N=C3N | 198.22 | Positive | -21.8 |  | (67) |
|  | 147815-03-0 | C1=CON=C1N | 84.08 | Negative | 21.1 |  | (5) |
|  | 148193-30-0 | C1=CC2=C(C=C1N)C(Cl)=NS2 | 184.65 | Positive | -7.1 |  | (47) |
|  | 148193-31-1 | C1=CC2=C(C(=C1)N)SN=C2Cl | 184.65 | Positive | -10.1 |  | (47) |
|  | 148193-33-3 | COC=2C=1C=CC(=CC=1SN=2)N | 180.23 | Positive | -0.4 |  | (47) |
|  | 148193-37-7 | C1=CC2=C(C=C1N)SNC2=O | 166.20 | Negative | 11.5 |  | (47) |
|  | 153954-29-1 | CC=2C=NC=1C=C(CO)C3=C(C=1N=2)N=C(N)N3C | 243.26 | Positive | -22.3 |  | (68) |
|  | 154028-32-7 | COC2=CC=CC(C=CC1=CC=C(C=C1)N)=C2 | 225.29 | Positive | -17.9 |  | (31) |
|  | 155014-05-4 | C=12C(=NC=C(C=1)F)C=CC=C2N | 162.16 | Positive | -5.8 |  | (69) |
|  | 156215-58-6 | CN3C2=CC1=CC=CC=C1N=C2N=C3N | 198.22 | Positive | -5.8 |  | (36) |
|  | 156243-39-9 | CC=2C=NC1=CC3=C(C(C)=C1N=2)N(C)C(N)=N3 | 227.27 | Positive | -10.9 |  | (70) |
|  | 157730-35-3 | CN3C=2C=C1C=CN=CC1=NC=2N=C3N | 199.21 | Positive | -0.5 |  | (36) |
|  | 157996-59-3 | C1=CC=C3C(=C1)C(=C2C=C(C=CC2=N3)Br)N | 273.13 | Positive | -12.7 |  | (34) |
|  | 159783-22-9 | C1(=C(C(=CN=C1)F)N)F | 130.10 | Negative | 22.0 |  | (6) |
|  | 161697-03-6 | CC1=C(C=CC2=C1N=C(C)C(C)=N2)N | 187.24 | Positive | -10.6 |  | (71) |
|  | 166964-09-6 | CC=1C(=C(N)ON=1)Cl | 132.55 | Positive | 7.6 |  | (5) |
|  | 194479-75-9 | CC=1C=CC=3C(C=1)=C(C=2C=C(C)C=CC=2N=3)N | 222.29 | Positive | -22.5 |  | https://pubchem.ncbi.nlm.nih.gov/bioassay/1259407#sid=363905935 |
|  | 194479-79-3 | C1=CC=C3C(=C1)C(=C2C=C(C=CC2=N3)I)N | 320.13 | Positive | -12.2 |  | (11) |
|  | 268568-11-2 | CCOC(C=1C=C(C=CC=1N)I)=O | 291.09 | Negative | 11.4 |  | (6) |
|  | 314084-63-4 | CCC=1C=C(C=C(CC)C=1N)C2=CC=CC=C2 | 225.33 | Negative | -19.5 |  | (42) |
|  | 324579-90-0 | C1CC1C2=CSC(=N2)N | 140.21 | Positive | -13.4 | Ring Opening | (5) |
|  | 389104-55-6 | CC(C)(C)C=1C2=CC=CC=C2C=CC=1N | 199.29 | Negative | -22.2 |  | (42) |
|  | 389104-59-0 | CC(C)(C)C=2C=3CC1=CC=CC=C1C=3C=CC=2N | 237.34 | Negative | -23.4 |  | (42) |
|  | \| 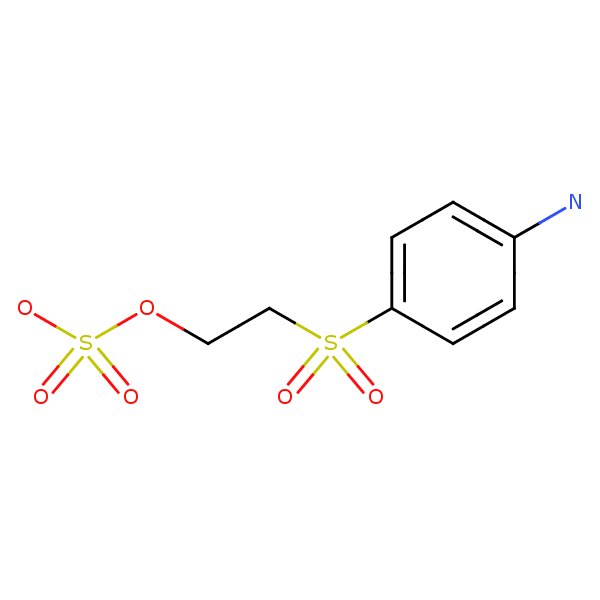389104-60-3 \| \| --- \| | CCC1=C(C=CC(=C1)C2=CC=CC=C2)N | 197.28 | Positive | -16.4 |  | (42) |
|  | 389104-57-8 | CC(C)C=2C=3CC1=CC=CC=C1C=3C=CC=2N | 223.31 | Positive | -21.1 |  | (42) |
|  | 389104-62-5 | CC(C)C=1C=C(C=C(C(C)C)C=1N)C2=CC=CC=C2 | 253.38 | Negative | -21.3 |  | (42) |
|  | 438056-69-0 | C=1C=C(C=CC=1N)N2CCOCC2=O | 192.21 | Positive | -20.1 |  | https://echa.europa.eu/nl/registration-dossier/-/registered-dossier/6284/7/7/2 |
|  | 827614-64-2 | CC2(C)C(C)(C)OB(C=1C=CC(N)=NC=1)O2 | 220.08 | Positive | 6.9 |  | (6) |
|  | 1002726-62-6 | C1=CC2=C(N=C1N)NC(C(O2)(C)C)=O | 193.20 | Negative | -3.9 |  | (6) |
|  | 1186310-74-6 | C=12C=COC=1C(=CC=N2)N | 134.14 | Negative | 9.7 |  | (6) |
|  | NO_CAS | CC(C)(C=1C=CC=C(C=1)N)C(=O)OC | 193.2 | Positive | -2.4 |  | - |
|  | NO_CAS | CC=1C=C(C=C(C)C=1Br)N | 200.1 | Positive | 1.1 |  |  |
|  | NO_CAS | C1=CC(=C(C=C1C#N)N)O | 134.1 | Positive | -3.7 |  |  |
|  | NO_CAS | C1=CC(=C(C=C1C(N)=O)N)F | 154.1 | Negative | 4.6 |  |  |
|  | NO_CAS | C1=CC(=C(C=C1C(O)=O)N)F | 155.1 | Positive | 7.3 |  |  |
|  | NO_CAS | COC=1C=CC(=CC=1N)C#N | 148.2 | Positive | -6.7 |  |  |
|  | NO_CAS | Undisclosed | 430.4 | Negative | 10.3 |  |  |
|  | NO_CAS | Undisclosed | 151.2 | Positive | -8.9 |  |  |
|  | NO_CAS | Undisclosed | 293.1 | Negative | 25.0 |  |  |
|  | NO_CAS | Undisclosed | 321.1 | Negative | 22.4 |  |  |
|  | NO_CAS | Undisclosed | 381.1 | Negative | 20.3 |  |  |
|  | NO_CAS | Undisclosed | 471.1 | Negative | 22.4 |  |  |
|  | NO_CAS | Undisclosed | 352.1 | Negative | -40.3 | Ring Opening |  |
|  | NO_CAS | Undisclosed | 414.0 | Negative | -22.2 | Ring Opening |  |
|  | NO_CAS | Undisclosed | 159.1 | Positive | -7.4 |  |  |
|  | NO_CAS | Undisclosed | 181.0 | Positive | -59.1 |  |  |
|  | NO_CAS | Undisclosed | 234.1 | Positive | -8.6 | Two or more amines detected. The first site is used. |  |
|  | NO_CAS | Undisclosed | 227.1 | Positive | -4.5 | Two or more amines detected. The first site is used. |  |
|  | NO_CAS | Undisclosed | 346.1 | Negative | 2.9 | Two or more amines detected. The first site is used. |  |
|  | NO_CAS | Undisclosed | 129.0 | Negative | 9.0 |  |  |
|  | NO_CAS | Undisclosed | 366.1 | Negative | -6.2 | Two or more amines detected. The first site is used. |  |
|  | NO_CAS | Undisclosed | 248.1 | Negative | -34.4 |  |  |
|  | NO_CAS | Undisclosed | 171.0 | Negative | 15.6 |  |  |
|  | NO_CAS | Undisclosed | 348.0 | Positive | -26.4 | Two or more amines detected. The first site is used. |  |
|  | NO_CAS | Undisclosed | 352.0 | Positive | -1.3 | Two or more amines detected. The first site is used. |  |
|  | NO_CAS | Undisclosed | 111.1 | Negative | -0.3 |  |  |
|  | NO_CAS | Undisclosed | 282.0 | Negative | 36.3 |  |  |
|  | NO_CAS | Undisclosed | 111.1 | Positive | -1.6 |  |  |
|  | NO_CAS | Undisclosed | 231.1 | Negative | 2.6 |  |  |
|  | 27492-84-8 | COC=1C=C(C=CC=1C(=O)OC)N | 181.2 | Negative | 9.7 |  |  |
|  | 1190837-07-0 | COC=1C=C2C(=CC=1C(=O)OC)C(=CC=N2)OC=3C=CC(=CC=3F)N | 342.3 | Positive | -3.7 |  |  |
|  | 399-96-2 | C1=CC(=C(C=C1N)F)O | 127.1 | Positive | -5.0 |  |  |
|  | 1190837-13-8 | CNC(C=1C=C2C(=CC=1OC)N=CC=C2OC=3C=CC(=CC=3F)N)=O | 341.3 | Positive | -5.7 |  |  |
|  | NO_CAS | Undisclosed | 159.2 | Positive | -11.8 |  |  |
|  | NO_CAS | Undisclosed | 129.1 | Negative | 7.5 |  |  |
|  | NO_CAS | Undisclosed | 145.6 | Positive | 4.2 |  |  |
|  | NO_CAS | Undisclosed | 147.1 | Negative | 16.5 |  |  |
|  | NO_CAS | Undisclosed | 125.1 | Positive | -2.4 |  |  |
|  | NO_CAS | Undisclosed | 173.2 | Positive | -18.4 |  |  |
|  | NO_CAS | Undisclosed | 197.6 | Positive | 13.7 |  |  |
|  | NO_CAS | Undisclosed | 161.1 | Positive | 5.9 |  |  |
|  | NO_CAS | Undisclosed | 145.6 | Negative | 14.2 |  |  |
|  | NO_CAS | Undisclosed | 163.6 | Negative | 11.9 |  |  |
|  | NO_CAS | Undisclosed | 129.1 | Negative | 18.0 |  |  |
|  | NO_CAS | Undisclosed | 144.1 | Positive | 27.5 |  |  |
|  | NO_CAS | Undisclosed | 190.0 | Positive | 3.9 |  |  |
|  | NO_CAS | Undisclosed | 208.0 | Negative | 11.8 |  |  |
|  | NO_CAS | Undisclosed | 136.1 | Positive | 8.3 |  |  |
|  | NO_CAS | Undisclosed | 179.1 | Negative | 10.1 |  |  |
|  | NO_CAS | Undisclosed | 163.6 | Positive | 4.3 |  |  |
|  | NO_CAS | Undisclosed | 220.3 | Negative | -29.3 |  |  |
|  | NO_CAS | Undisclosed | 234.3 | Positive | -29.3 |  |  |
|  | NO_CAS | Undisclosed | 378.4 | Positive | 9.4 |  |  |
|  | NO_CAS | Undisclosed | 414.3 | Negative | 19.9 |  |  |
|  | NO_CAS | Undisclosed | 416.4 | Negative | 20.7 |  |  |
|  | NO_CAS | Undisclosed | 434.4 | Negative | 23.6 |  |  |
|  | NO_CAS | Undisclosed | 466.4 | Negative | 21.2 |  |  |
|  | NO_CAS | Undisclosed | 97.1 | Negative | -22.6 |  |  |
|  | NO_CAS | Undisclosed | 133.1 | Negative | -13.1 |  |  |
|  | NO_CAS | Undisclosed | 387.4 | Negative | -3.9 |  |  |
|  | NO_CAS | Undisclosed | 329.2 | Negative | -4.5 |  |  |
|  | NO_CAS | Undisclosed | 446.4 | Negative | 15.2 |  |  |
|  | NO_CAS | Undisclosed | 472.4 | Negative | 10.9 |  |  |
|  | NO_CAS | Undisclosed | 472.4 | Negative | -1.3 |  |  |
|  | NO_CAS | Undisclosed | 492.4 | Negative | 2.3 |  |  |
|  | NO_CAS | Undisclosed | 502.4 | Negative | 18.8 |  |  |
|  | NO_CAS | Undisclosed | 490.5 | Negative | 9.5 |  |  |
|  | NO_CAS | Undisclosed | 460.4 | Negative | -3.6 |  |  |
|  | NO_CAS | Undisclosed | 472.4 | Negative | 12.8 |  |  |
|  | NO_CAS | Undisclosed | 151.2 | Positive | 0.9 |  |  |
|  | NO_CAS | Undisclosed | 141.6 | Negative | -0.2 |  |  |
|  | NO_CAS | Undisclosed | 452.4 | Negative | 13.1 |  |  |
|  | NO_CAS | Undisclosed | 249.3 | Negative | 0.7 |  |  |
|  | NO_CAS | Undisclosed | 355.4 | Negative | 25.9 |  |  |
|  | NO_CAS | Undisclosed | 248.3 | Negative | -2.0 |  |  |
|  | NO_CAS | Undisclosed | 464.9 | Negative | 32.0 |  |  |
|  | NO_CAS | Undisclosed | 451.3 | Negative | 32.7 |  |  |
|  | NO_CAS | Undisclosed | 357.4 | Negative | 1.5 |  |  |
|  | NO_CAS | Undisclosed | 401.4 | Negative | 27.4 |  |  |
|  | NO_CAS | Undisclosed | 242.3 | Negative | -43.2 | Ring Opening |  |
|  | NO_CAS | Undisclosed | 329.3 | Negative | 10.5 |  |  |
|  | NO_CAS | Undisclosed | 221.7 | Negative | 9.5 |  |  |
|  | NO_CAS | Undisclosed | 395.3 | Negative | -9.5 |  |  |
|  | NO_CAS | Undisclosed | 367.2 | Negative | -6.2 |  |  |
|  | NO_CAS | Undisclosed | 208.2 | Negative | 9.8 |  |  |
|  | NO_CAS | Undisclosed | 194.2 | Positive | -27.7 |  |  |
|  | NO_CAS | Undisclosed | 402.5 | Positive | -15.3 |  |  |
|  | NO_CAS | Undisclosed | 204.2 | Positive | -13.0 |  |  |
|  | NO_CAS | Undisclosed | 162.1 | Negative | 20.6 |  |  |
|  | NO_CAS | Undisclosed | 175.1 | Positive | 14.9 |  |  |
|  | NO_CAS | Undisclosed | 177.1 | Negative | 30.9 |  |  |
|  | NO_CAS | Undisclosed | 179.1 | Positive | 24.4 |  |  |
|  | NO_CAS | Undisclosed | 197.1 | Positive | 33.4 |  |  |
|  | NO_CAS | Undisclosed | 202.1 | Positive | 25.1 |  |  |
|  | NO_CAS | Undisclosed | 203.1 | Negative | 29.0 |  |  |
|  | NO_CAS | Undisclosed | 215.0 | Negative | 31.9 |  |  |
|  | NO_CAS | Undisclosed | 218.2 | Negative | 23.6 |  |  |
|  | NO_CAS | Undisclosed | 219.1 | Negative | 12.3 |  |  |
|  | NO_CAS | Undisclosed | 241.0 | Negative | 32.6 |  |  |
|  | NO_CAS | Undisclosed | 281.3 | Negative | -23.7 |  |  |
|  | NO_CAS | Undisclosed | 341.3 | Negative | 9.8 |  |  |
|  | NO_CAS | Undisclosed | 370.4 | Negative | 28.3 |  |  |
|  | NO_CAS | Undisclosed | 396.4 | Negative | 30.4 |  |  |
|  | NO_CAS | Undisclosed | 410.5 | Negative | 29.1 |  |  |
|  | NO_CAS | Undisclosed | 439.4 | Negative | 42.2 |  |  |
|  | NO_CAS | Undisclosed | 439.5 | Negative | 29.3 |  |  |
|  | NO_CAS | Undisclosed | 499.5 | Negative | -24.9 |  |  |
|  | NO_CAS | Undisclosed | 412.3 | Negative | 0.9 |  |  |
|  | \| 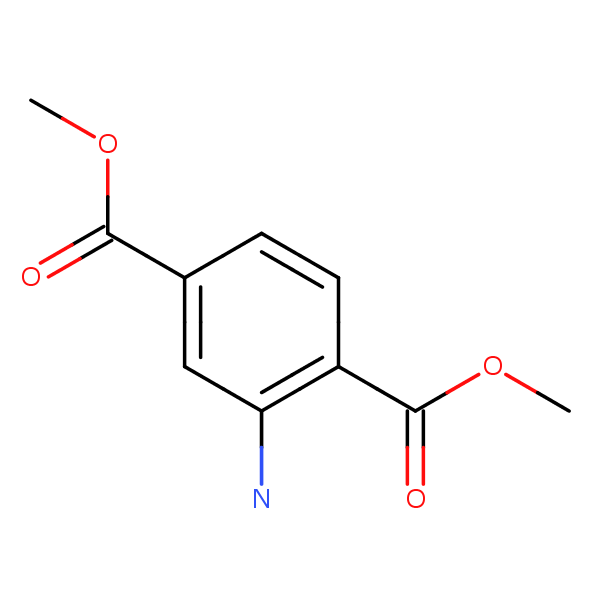NO_CAS \| \| --- \| | Undisclosed | 385.5 | Positive | -9.7 |  |  |
|  | NO_CAS | Undisclosed | 397.4 | Negative | 16.5 |  |  |
|  | NO_CAS | Undisclosed | 401.4 | Positive | -5.7 |  |  |
|  | NO_CAS | Undisclosed | 399.9 | Positive | -7.2 |  |  |
|  | NO_CAS | Undisclosed | 390.5 | Positive | 0.1 |  |  |
|  | NO_CAS | Undisclosed | 398.3 | Negative | 1.1 |  |  |
|  | NO_CAS | Undisclosed | 396.4 | Negative | 0.1 |  |  |
|  | NO_CAS | Undisclosed | 421.4 | Negative | 9.1 |  |  |
|  | NO_CAS | Undisclosed | 370.4 | Negative | -1.8 |  |  |
|  | NO_CAS | Undisclosed | 380.3 | Negative | 0.8 |  |  |
|  | NO_CAS | Undisclosed | 399.3 | Negative | -0.9 |  |  |
|  | NO_CAS | Undisclosed | 411.4 | Negative | -1.7 |  |  |
|  | NO_CAS | Undisclosed | 405.3 | Negative | 6.0 |  |  |
|  | NO_CAS | Undisclosed | 410.4 | Positive | -11.9 |  |  |
|  | NO_CAS | Undisclosed | 405.3 | Negative | 9.9 |  |  |
|  | NO_CAS | Undisclosed | 396.3 | Positive | -12.3 |  |  |
|  | NO_CAS | Undisclosed | 155.1 | Positive | 19.6 |  |  |
|  | NO_CAS | Undisclosed | 258.0 | Negative | 16.0 |  |  |
|  | NO_CAS | Undisclosed | 204.3 | Negative | -5.5 |  |  |
|  | NO_CAS | Undisclosed | 220.0 | Negative | 16.4 |  |  |
|  | NO_CAS | Undisclosed | 118.1 | Negative | 10.0 |  |  |
|  | NO_CAS | NC1=CC=C(I)C=C1F | 237.0 | Negative | 5.2 |  |  |
|  | NO_CAS | NC1=CC=C(C=C1F)N1C=CC=N1 | 177.2 | Positive | -14.4 |  |  |
|  | NO_CAS | NC1=NC=CC=C1Br | 173.0 | Negative | 15.2 |  |  |
|  | NO_CAS | NC1=NC=CC=C1C1=CC=C(OC2=CC=CC=C2)C=C1 | 262.3 | Negative | -9.8 |  |  |
|  | NO_CAS | NC1=CC=C(F)C=C1Cl | 145.6 | Positive | 0.5 |  |  |
|  | NO_CAS | NC1=CC(O)=CC=C1F | 127.1 | Positive | 2.9 |  |  |
|  | NO_CAS | CCOC(=O)C1=C(N)SC(=C1C)C1=CC=C(C=C1)[N+]([O-])=O | 306.3 | Positive | -8.5 |  |  |
|  | NO_CAS | COC1=NN=C(N)C=C1 | 125.1 | Negative | -2.7 |  |  |
|  | NO_CAS | O(C)c1c(OC)cc2nccc(Oc3cnc(N)cc3)c2c1 | 297.3 | Positive | -6.2 |  |  |
|  | NO_CAS | Clc1c(-c2c(N)[nH]nc2C)ccc(OC)c1 | 237.7 | Negative | -19.1 |  |  |
|  | NO_CAS | Brc1c(N)cc(F)cc1 | 190.0 | Negative | 12.2 |  |  |
|  | NO_CAS | FC(F)(F)c1c(N)cc(C2(C(=O)OC)CC2)cc1 | 259.2 | Negative | 13.3 |  |  |
|  | NO_CAS | O(c1ccc(N2C(=O)N([C@]3CNCC3)c3ncnc(N)c23)cc1)c1ccccc1 | 387.4 | Negative | -8.5 |  |  |
|  | NO_CAS | Fc1ccc(Oc2cc(Oc3ccc(C(C(=O)OC)(C)C)cc3)cc(N)c2)cc1 | 395.4 | Negative | 2.5 |  |  |
|  | NO_CAS | O(c1ncc(N)cn1)c1ccc(B2OC(C)(C)C(C)(C)O2)cc1 | 313.2 | Negative | -12.4 |  |  |
|  | NO_CAS | Brc1c(N)cc(C(F)(F)F)cc1 | 240.0 | Negative | 14.2 |  |  |
|  | NO_CAS | FC(F)(F)c1cc(N)c(-c2cnccc2)cc1 | 238.2 | Negative | 2.8 |  |  |
|  | NO_CAS | Brc1c(N)ncc(F)c1 | 191.0 | Negative | 13.6 |  |  |
|  | NO_CAS | O=C(OCC)CCCc1c(N)cc(C#N)cc1 | 232.3 | Negative | -0.4 |  |  |
|  | NO_CAS | Oc1cnc(N)cc1 | 110.1 | Negative | -3.1 |  |  |
|  | NO_CAS | Fc1nc(N)ccc1 | 112.1 | Negative | 20.5 |  |  |
|  | NO_CAS | Fc1c(C(=O)O)c(N)ccc1 | 155.1 | Negative | 17.1 |  |  |
|  | NO_CAS | O=C(O)c1cc(C)c(N)cc1 | 151.2 | Negative | 6.4 |  |  |
|  | NO_CAS | Clc1c(N)cc(C(O)=N)c(F)c1 | 188.6 | Positive | -0.6 |  |  |
|  | NO_CAS | Clc1c(C(O)=N)cc(N)c(F)c1 | 188.6 | Positive | 0.0 |  |  |
|  | NO_CAS | Fc1c(N)cc(C(O)=NC)c(F)c1 | 186.2 | Negative | -2.0 |  |  |
|  | NO_CAS | Fc1c(C(O)=N)cc(N)cc1 | 154.1 | Positive | -1.9 |  |  |
|  | NO_CAS | Nc1cnc(C)cc1 | 108.1 | Negative | -2.7 |  |  |
|  | NO_CAS | Nc1onc(C)c1 | 98.1 | Positive | -36.9 | Ring Opening |  |
|  | NO_CAS | Clc1c(N)c(Cl)cc(OC(F)(F)F)c1 | 246.0 | Negative | 1.4 |  |  |
|  | NO_CAS | O=C(C#CC)N1C[C@](N2C(=O)N(c3ccc(Oc4ccccc4)cc3)c3c(N)ncnc23)CC1 | 453.5 | Negative | -6.6 |  |  |
|  | NO_CAS | FC(F)(F)c1cc(NC(O)=Nc2cnc(Oc3ccc(-c4c(N)ncc(F)c4)cc3)nc2)c(-c2cnccc2)cc1 | 561.5 | Negative | -8.1 |  |  |
|  | NO_CAS | Undisclosed | 395.4 | Positive | -3.5 |  |  |
|  | NO_CAS | Undisclosed | 306.4 | Negative | -15.0 |  |  |
|  | NO_CAS | Undisclosed | 350.5 | Positive | -4.6 |  |  |
|  | NO_CAS | Undisclosed | 240.3 | Positive | -26.1 |  |  |
|  | NO_CAS | Undisclosed | 367.4 | Negative | 8.1 |  |  |
|  | NO_CAS | Undisclosed | 339.3 | Positive | 5.8 |  |  |
|  | NO_CAS | Undisclosed | 353.3 | Positive | 8.8 |  |  |
|  | NO_CAS | Undisclosed | 382.4 | Negative | 4.3 |  |  |
|  | NO_CAS | Undisclosed | 382.4 | Negative | 4.2 |  |  |
|  | NO_CAS | Undisclosed | 400.4 | Negative | 11.5 |  |  |
|  | NO_CAS | Undisclosed | 400.4 | Negative | 11.4 |  |  |
|  | NO_CAS | Undisclosed | 382.4 | Negative | 10.4 |  |  |
|  | NO_CAS | Undisclosed | 243.6 | Negative | -81.6 |  |  |
|  | NO_CAS | Undisclosed | 500.5 | Negative | -13.9 |  |  |
|  | NO_CAS | Undisclosed | 309.8 | Negative | 6.4 |  |  |
|  | NO_CAS | Undisclosed | 260.2 | Negative | 1.2 |  |  |
|  | NO_CAS | Undisclosed | 246.1 | Negative | 7.1 |  |  |
|  | NO_CAS | Undisclosed | 413.3 | Negative | 8.1 |  |  |
|  | NO_CAS | Undisclosed | 428.4 | Negative | -0.4 |  |  |
|  | NO_CAS | Undisclosed | 412.4 | Negative | 3.1 |  |  |
|  | NO_CAS | Undisclosed | 412.4 | Negative | 4.4 |  |  |
|  | NO_CAS | Undisclosed | 350.4 | Negative | -8.8 |  |  |
|  | NO_CAS | CCOC(=O)C1=C(N(N=C1)[C@H]1CCOC1)C1=C(N)C=C(C=C1)C1=C(C)C=NC(OC)=C1C | 436.5 | Negative | -1.2 |  |  |
|  | NO_CAS | Undisclosed | 374.2 | Negative | -134.1 |  |  |
|  | NO_CAS | Undisclosed | 247.7 | Negative | -8.9 |  |  |
|  | NO_CAS | Undisclosed | 252.3 | Negative | -3.4 |  |  |
|  | NO_CAS | Undisclosed | 265.3 | Negative | -0.6 |  |  |
|  | NO_CAS | COC(=O)C1=CC(Br)=CC(N)=C1C | 244.1 | Negative | 3.0 |  |  |
|  | NO_CAS | Undisclosed | 623.7 | Negative | -179.5 |  |  |
|  | NO_CAS | Undisclosed | 435.6 | Negative | -23.4 |  |  |
|  | NO_CAS | Undisclosed | 337.9 | Negative | -20.0 |  |  |
|  | NO_CAS | Undisclosed | 281.4 | Positive | -6.9 |  |  |
|  | NO_CAS | Undisclosed | 260.2 | Positive | -10.9 |  |  |
|  | NO_CAS | Undisclosed | 411.5 | Positive | -16.2 |  |  |
|  | NO_CAS | Undisclosed | 430.0 | Positive | -18.3 |  |  |
|  | NO_CAS | Undisclosed | 267.7 | Positive | -9.1 |  |  |
|  | NO_CAS | Undisclosed | 441.8 | Positive | -14.4 |  |  |
|  | NO_CAS | NC1=CC(Cl)=C(CC2=CC=C(N)C=C2Cl)C=C1 | 267.2 | Positive | -6.3 |  |  |
|  | NO_CAS | undisclosed | 304.2 | Positive | -33.4 |  |  |
|  | NO_CAS | undisclosed | 266.1 | Positive | -39.0 |  |  |
|  | NO_CAS | undisclosed | 243.0 | Positive | 6.6 |  |  |
|  | 4815-30-9 | CCOC(C=1C(C)=C(C(=O)OCC)SC=1N)=O | 257.0 | Positive | -3.4 |  |  |
|  | 1152519-69-1 | C1CC1C2=NC=C(C=N2)N | 135.0 | Negative | -3.2 |  |  |
|  | NO_CAS | undisclosed | 276.1 | Negative | -8.5 | Ring Opening |  |
|  | NO_CAS | undisclosed | 536.1 | Negative | -6.7 |  |  |
|  | NO_CAS | undisclosed | 380.1 | Negative | -9.6 | Ring Opening |  |
|  | NO_CAS | undisclosed | 399.1 | Negative | -8.1 | Ring Opening |  |
|  | 1603-91-4 | C=1(N=C(SC=1)N)C | 114 | Negative | -11.1 | Ring Opening |  |
|  | NO_CAS | undisclosed | 288.1 | Positive | -22.0 |  |  |
|  | 767-63-5 | C=1C=C(C=2C(C=1)=NON=2)N | 135.0 | Negative | 3.4 |  |  |
|  | NO_CAS | undisclosed | 160.0 | Positive | -15.8 |  |  |
|  | NO_CAS | undisclosed | 110.0 | Positive | -23.2 |  |  |
|  | NO_CAS | undisclosed | 292.1 | Negative | -6.2 |  |  |
|  | NO_CAS | undisclosed | 274 | Positive | -11.0 | Ring Opening |  |
|  | 948571-47-9 | C(CO)N1C=C(C=N1)N | 127.0 | Positive | -20.9 |  |  |
|  | 1346136-01-3 | COC(C2=CC=C(CCC1=CC=C(C=C1)N)C=C2)=O | 255.1 | Positive | -4.6 |  |  |
|  | NO_CAS | undisclosed | 434.1 | Negative | -18.7 |  |  |
|  | NO_CAS | undisclosed | 347.1 | Negative | -11.6 |  |  |
|  | \| 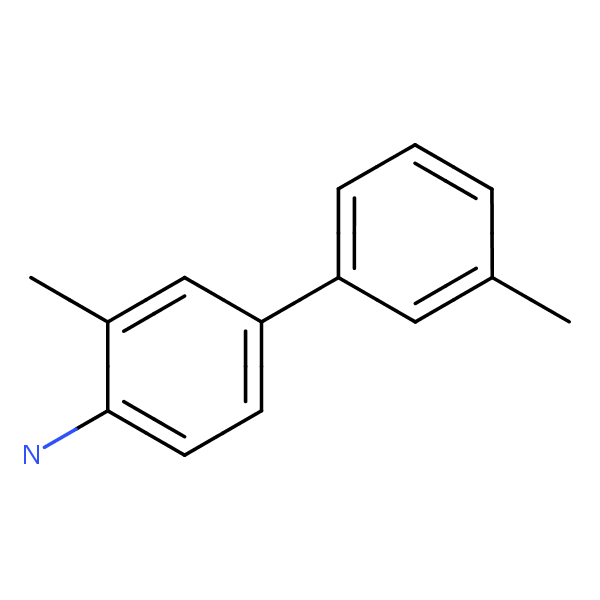NO_CAS \| \| --- \| | undisclosed | 347.1 | Negative | -13.3 |  |  |
|  | 368-71-8 | C1=CC(=C(C=C1C(F)(F)F)N)N | 176.0 | Positive | -18.0 | Two or more amines detected. The first site is used. |  |
|  | NO_CAS | undisclosed | 215.9 | Positive | -12.3 | Ring Opening |  |
|  | NO_CAS | undisclosed | 293.8 | Positive | -5.4 |  |  |
|  | NO_CAS | undisclosed | 382.1 | Negative | -39.2 |  |  |
|  | NO_CAS | undisclosed | 236.1 | Negative | 7.0 |  |  |
|  | NO_CAS | undisclosed | 274.2 | Positive | -33.3 |  |  |
|  | 6315-89-5 | COC=1C=CC(=CC=1OC)N | 153.0 | Negative | -17.9 |  |  |
|  | NO_CAS | undisclosed | 437.2 | Negative | 0.9 |  |  |
|  | NO_CAS | undisclosed | 232.9 | Negative | 13.9 |  |  |
|  | NO_CAS | undisclosed | 358.8 | Negative | 17.0 |  |  |
|  | 2241721-73-1 | BrC1=C(F)C(N)=C(C(N)=O)C=C1I | 357.8 | Negative | 12.9 |  |  |
|  | NO_CAS | Undisclosed | Less than 500 | Positive | -19.2 |  |  |
|  | NO_CAS | Undisclosed | Less than 500 | Negative | -2.3 |  |  |
|  | 873056-62-3 | C1=CC=C(C(=C1)C=2C=CC(=C(C=2)F)F)N | Less than 500 | Positive | -3.9 |  |  |
|  | NO_CAS | Undisclosed | Greater than 500 | Negative | -35.9 |  |  |
|  | NO_CAS | Undisclosed | Less than 500 | Negative | -16.3 |  |  |
|  | 124409-98-9 | CC(COC1=C(C=CC(=C1F)F)N)O | Less than 500 | Positive | -5.4 |  |  |
|  | NO_CAS | Undisclosed | Less than 500 | Positive | -13.6 | Two or more amines detected. The first site is used. |  |
|  | NO_CAS | Undisclosed | Greater than 500 | Positive | 28.4 |  |  |
|  | NO_CAS | CCCCCCSC1=CC=C(C=C1)N | Less than 500 | Positive | -23.0 |  |  |
|  | NO_CAS | Undisclosed | Less than 500 | Positive | 38.1 |  |  |
|  | NO_CAS | Undisclosed | Less than 500 | Negative | -30.3 |  |  |
|  | NO_CAS | Undisclosed | Less than 500 | Negative | -11.6 | Ring Opening |  |
|  | NO_CAS | Undisclosed | Less than 500 | Negative | -4.7 |  |  |
|  | NO_CAS | Undisclosed | Less than 500 | Negative | 32.6 |  |  |
|  | NO_CAS | Undisclosed | Greater than 500 | Positive | -26.7 | Two or more amines detected. The first site is used. |  |
|  | NO_CAS | Undisclosed | Less than 500 | Negative | 0.9 |  |  |
|  | NO_CAS | Undisclosed | Less than 500 | Negative | 19.0 |  |  |
|  | NO_CAS | Undisclosed | Greater than 500 | Negative | -19.2 |  |  |
|  | NO_CAS | Undisclosed | Less than 500 | Positive | -4.3 | Two or more amines detected. The first site is used. |  |
|  | NO_CAS | Undisclosed | Less than 500 | Negative | 21.5 |  |  |
|  | NO_CAS | Undisclosed | Less than 500 | Negative | 29.1 |  |  |
|  | NO_CAS | Undisclosed | Greater than 500 | Negative | -1.1 |  |  |
|  | NO_CAS | Undisclosed | Less than 500 | Negative | 4.3 |  |  |
|  | NO_CAS | Undisclosed | Less than 500 | Negative | 9.2 |  |  |
|  | NO_CAS | Undisclosed | Less than 500 | Negative | -12.3 | Two or more amines detected. The first site is used. |  |
|  | NO_CAS | Undisclosed | Less than 500 | Negative | -6.5 | Ring Opening |  |
|  | NO_CAS | Undisclosed | Less than 500 | Positive | -1.0 |  |  |
|  | NO_CAS | Undisclosed | Less than 500 | Negative | 9.1 |  |  |
|  | NO_CAS | Undisclosed | Less than 500 | Negative | -21.2 |  |  |
|  | NO_CAS | Undisclosed | Less than 500 | Positive | -13.6 |  |  |
|  | NO_CAS | Undisclosed | Less than 500 | Negative | -7.2 | Ring Opening |  |
|  | NO_CAS | CON=C(C(Cl)=O)C1=NC(=N)SN1 | Less than 500 | Positive | 13.3 |  |  |
|  | NO_CAS | Undisclosed | Less than 500 | Negative | -18.7 |  |  |
|  | NO_CAS | Undisclosed | Greater than 500 | Negative | -3.3 | Two or more amines detected. The first site is used. |  |
|  | 3224-15-5 | CC(CCOC=1C=C(C3=C(C=1N)C(C2=CC=CC=C2C3=O)=O)O)O | Less than 500 | Positive | -17.9 |  |  |
|  | 85720-86-1 | CC1=CC(=C(C=C1N=NC=2C=CC(=C(C=2)C(=O)O)O)OC)N | Less than 500 | Positive | -20.8 |  |  |
|  | NO_CAS | Undisclosed | Less than 500 | Negative | 18.2 |  |  |
|  | NO_CAS | Undisclosed | Less than 500 | Positive | -19.3 | Two or more amines detected. The first site is used. |  |
|  | NO_CAS | Undisclosed | Less than 500 | Positive | 3.2 |  |  |
|  | 40718-14-7 | C1=CC(=CC(=C1)OC=2C=CC(=CC=2Cl)N)C(F)(F)F | Less than 500 | Positive | -6.2 |  |  |
|  | NO_CAS | Undisclosed | Less than 500 | Negative | 4.4 |  |  |
|  | NO_CAS | Undisclosed | Less than 500 | Positive | -12.4 |  |  |
|  | NO_CAS | Undisclosed | Greater than 500 | Negative | 15.3 |  |  |
|  | \| 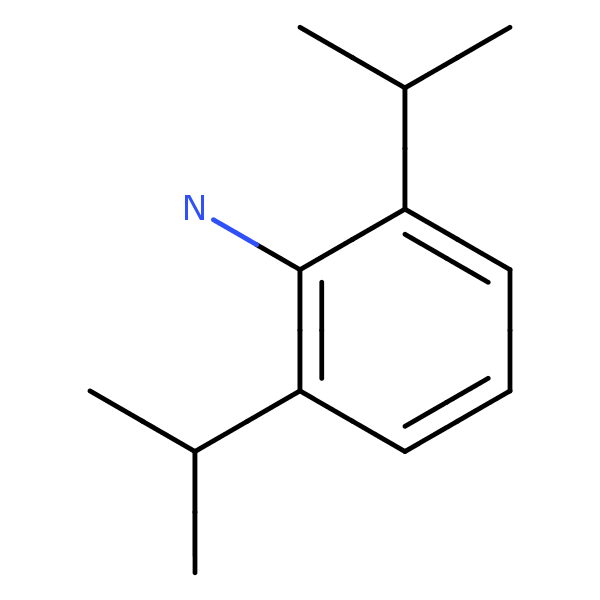NO_CAS \| \| --- \| | Undisclosed | Less than 500 | Negative | -28.7 | Two or more amines detected. The first site is used. |  |
|  | NO_CAS | Undisclosed | Less than 500 | Negative | -2.0 |  |  |
|  | NO_CAS | Undisclosed | Greater than 500 | Negative | -7.0 | Ring Opening |  |
|  | NO_CAS | Undisclosed | Less than 500 | Negative | -1.9 |  |  |
|  | NO_CAS | Undisclosed | Less than 500 | Positive | -19.8 |  |  |
|  | 108-72-5 | C1=C(C=C(C=C1N)N)N | Less than 500 | Positive | -2.1 | Two or more amines detected. The first site is used. |  |
|  | \| 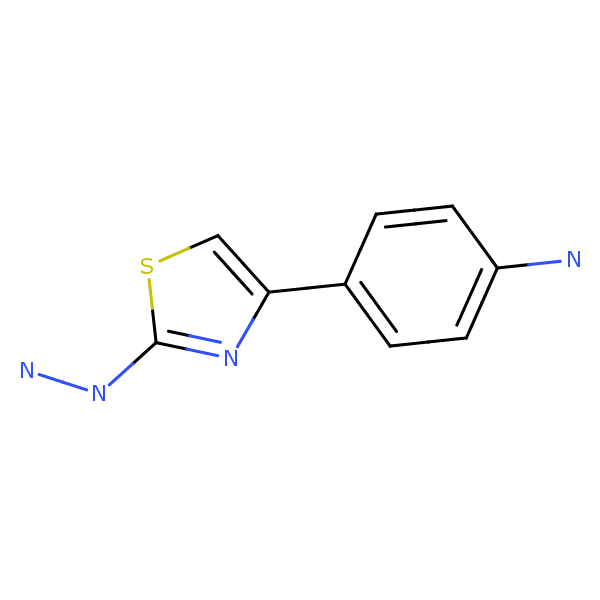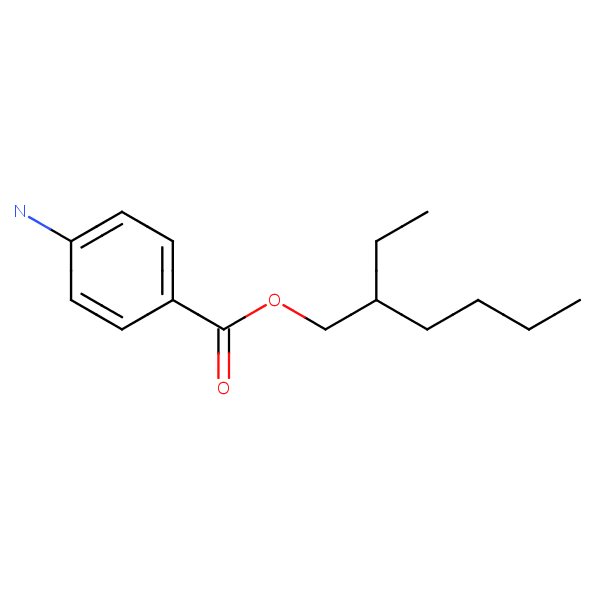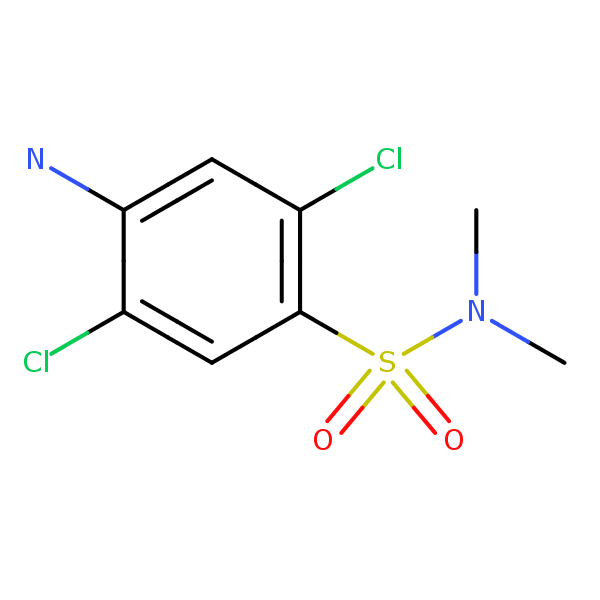NO_CAS \| \| --- \| | Undisclosed | Less than 500 | Negative | 7.2 |  |  |
|  | NO_CAS | C1=CC=C(C=C1)OCCOC=4C=C3N=C(C=2C=CC=C(C=2)N)NN3N=4 | Less than 500 | Positive | 5.0 |  |  |
|  | NO_CAS | Undisclosed | Less than 500 | Negative | -19.5 |  |  |
|  | NO_CAS | Undisclosed | Less than 500 | Negative | 22.7 |  |  |
|  | NO_CAS | Undisclosed | Greater than 500 | Negative | -12.1 |  |  |
|  | NO_CAS | Undisclosed | Less than 500 | Negative | -17.6 | Ring Opening |  |
|  | NO_CAS | Undisclosed | Less than 500 | Positive | 4.9 |  |  |
|  | NO_CAS | Undisclosed | Less than 500 | Negative | -22.2 | Two or more amines detected. The first site is used. |  |
|  | NO_CAS | Undisclosed | Less than 500 | Negative | -17.7 |  |  |
|  | NO_CAS | Undisclosed | Greater than 500 | Negative | -8.9 | Two or more amines detected. The first site is used. |  |
|  | NO_CAS | Undisclosed | Less than 500 | Negative | -19.8 | Ion2 QM Failed. |  |
|  | NO_CAS | Undisclosed | Less than 500 | Positive | 8.5 |  |  |
|  | \| 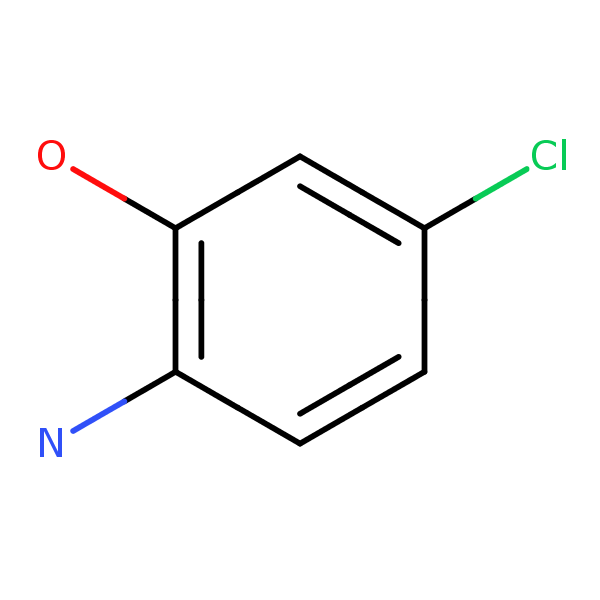NO_CAS \| \| --- \| | Undisclosed | Less than 500 | Negative | -24.1 |  |  |
|  | NO_CAS | Undisclosed | Less than 500 | Negative | 4.8 |  |  |
|  | NO_CAS | Undisclosed | Less than 500 | Negative | -20.1 | Two or more amines detected. The first site is used. |  |
|  | 24573-61-3 | C=1C=C(C=CC=1N)S(C=2C=CC(=CC=2S(N)(=O)=O)N(=O)=O)(=O)=O | Less than 500 | Positive | 18.3 |  |  |
|  | NO_CAS | Undisclosed | Less than 500 | Negative | 15.9 |  |  |
|  | NO_CAS | Undisclosed | Less than 500 | Negative | -13.8 |  |  |
|  | NO_CAS | Undisclosed | Less than 500 | Negative | 11.2 |  |  |
|  | NO_CAS | Undisclosed | Less than 500 | Positive | -20.3 |  |  |
|  | NO_CAS | Undisclosed | Less than 500 | Positive | -20.7 | Two or more amines detected. The first site is used. |  |
|  | NO_CAS | Undisclosed | Greater than 500 | Negative | 17.6 |  |  |
|  | NO_CAS | Undisclosed | Less than 500 | Negative | 14.6 |  |  |
|  | NO_CAS | Undisclosed | Greater than 500 | Negative | -3.7 |  |  |
|  | NO_CAS | Undisclosed | Less than 500 | Negative | 54.4 | Two or more amines detected. The first site is used. |  |
|  | NO_CAS | Undisclosed | Less than 500 | Negative | -11.3 | Two or more amines detected. The first site is used. |  |
|  | NO_CAS | Undisclosed | Less than 500 | Negative | -27.4 |  |  |
|  | NO_CAS | Undisclosed | Greater than 500 | Negative | -17.0 |  |  |
|  | NO_CAS | Undisclosed | Less than 500 | Positive | -17.5 | Two or more amines detected. The first site is used. |  |
|  | NO_CAS | Undisclosed | Less than 500 | Negative | -1.3 |  |  |
|  | NO_CAS | Undisclosed | Less than 500 | Positive | 17.0 | Two or more amines detected. The first site is used. |  |
|  | NO_CAS | Undisclosed | Less than 500 | Negative | 7.9 |  |  |
|  | 118430-73-2 | CC(C)(C)C=1C=C(N)N(C)N=1 | Less than 500 | Positive | -18.7 |  |  |
|  | NO_CAS | Undisclosed | Greater than 500 | Negative | -4.0 | Ring Opening |  |
|  | NO_CAS | Undisclosed | Less than 500 | Negative | -5.2 |  |  |
|  | NO_CAS | Undisclosed | Less than 500 | Positive | -3.6 |  |  |
|  | NO_CAS | Undisclosed | Less than 500 | Negative | 55.1 | Two or more amines detected. The first site is used. |  |
|  | NO_CAS | Undisclosed | Greater than 500 | Negative | -32.9 |  |  |
|  | NO_CAS | Undisclosed | Less than 500 | Negative | 1.3 |  |  |
|  | NO_CAS | Undisclosed | Less than 500 | Negative | -8.5 | Two or more amines detected. The first site is used. |  |
|  | \| 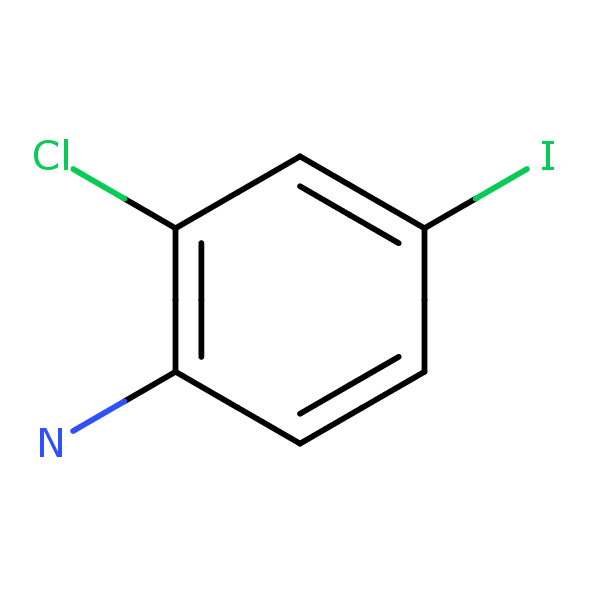NO_CAS \| \| --- \| | Undisclosed | Greater than 500 | Negative | 38.1 | Ring Opening |  |
|  | NO_CAS | Undisclosed | Less than 500 | Negative | -10.7 | Ring Opening |  |
|  | NO_CAS | Undisclosed | Less than 500 | Negative | -13.4 |  |  |
|  | NO_CAS | Undisclosed | Less than 500 | Negative | 31.2 |  |  |
|  | NO_CAS | Undisclosed | Less than 500 | Negative | 3.1 |  |  |
|  | NO_CAS | Undisclosed | Less than 500 | Positive | -23.2 |  |  |
|  | NO_CAS | Undisclosed | Less than 500 | Negative | 6.4 |  |  |
|  | NO_CAS | Undisclosed | Less than 500 | Positive | -27.7 |  |  |
|  | \| 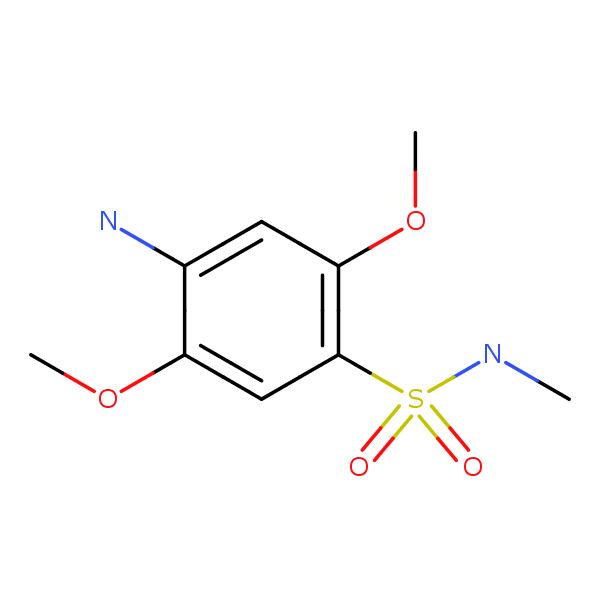NO_CAS \| \| --- \| | Undisclosed | Greater than 500 | Negative | 5.2 |  |  |
|  | NO_CAS | Undisclosed | Greater than 500 | Negative | -10.5 |  |  |
|  | NO_CAS | Undisclosed | Less than 500 | Negative | -17.6 | Two or more amines detected. The first site is used. |  |
|  | NO_CAS | Undisclosed | Less than 500 | Negative | -38.2 |  |  |
|  | NO_CAS | Undisclosed | Less than 500 | Positive | -21.9 |  |  |
|  | NO_CAS | Undisclosed | Greater than 500 | Negative | 10.5 |  |  |
|  | NO_CAS | Undisclosed | Greater than 500 | Negative | 63.8 | Ion2 QM Failed. |  |
|  | NO_CAS | Undisclosed | Less than 500 | Negative | -11.0 | Two or more amines detected. The first site is used. |  |
|  | NO_CAS | Undisclosed | Less than 500 | Negative | 56.1 |  |  |
|  | 621-95-4 | C(CC1=CC=C(C=C1)N)C2=CC=C(C=C2)N | Less than 500 | Positive | -10.3 | Two or more amines detected. The first site is used. |  |
|  | NO_CAS | Undisclosed | Less than 500 | Negative | 2.8 |  |  |
|  | NO_CAS | CCCCCCCCCCCCOC=1C=CC(=CC=1N)N | Less than 500 | Positive | -18.0 | Two or more amines detected. The first site is used. |  |
|  | NO_CAS | Undisclosed | Less than 500 | Negative | 27.4 |  |  |
|  | NO_CAS | Undisclosed | Less than 500 | Negative | -12.2 |  |  |
|  | NO_CAS | Undisclosed | Greater than 500 | Negative | -17.8 |  |  |
|  | NO_CAS | Undisclosed | Less than 500 | Negative | 1.5 |  |  |
|  | 97-28-9 | CC2=CC(=CC=C2CC1=CC=C(C=C1C)N)N | Less than 500 | Positive | -12.8 | Two or more amines detected. The first site is used. |  |
|  | \| 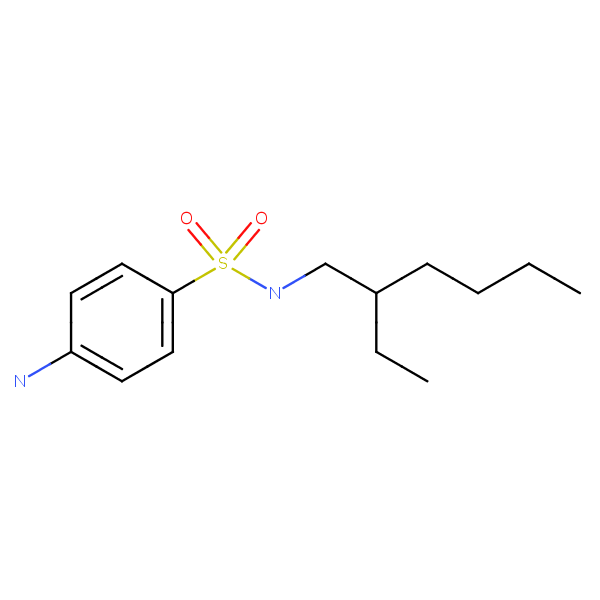NO_CAS \| \| --- \| | Undisclosed | Greater than 500 | Negative | 21.6 |  |  |
|  | NO_CAS | Undisclosed | Less than 500 | Negative | 8.4 |  |  |
|  | NO_CAS | Undisclosed | Less than 500 | Negative | -12.3 |  |  |
|  | NO_CAS | Undisclosed | Less than 500 | Negative | -20.6 |  |  |
|  | \| 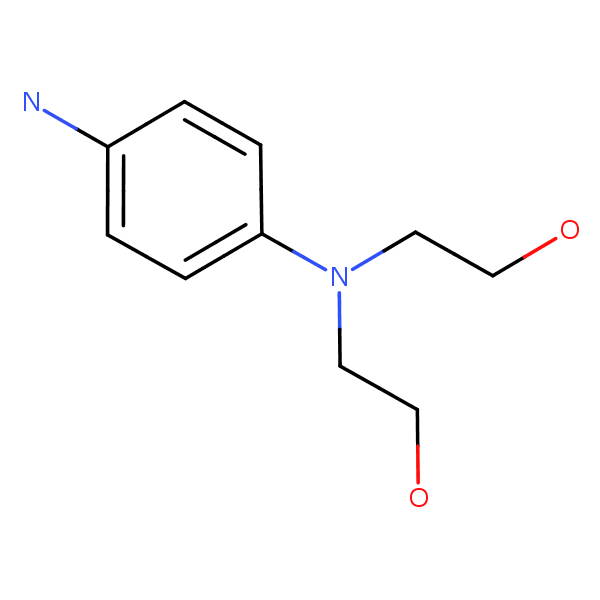NO_CAS \| \| --- \| | Undisclosed | Greater than 500 | Negative | -15.0 | Two or more amines detected. The first site is used. |  |
|  | NO_CAS | Undisclosed | Less than 500 | Negative | 28.9 |  |  |
|  | NO_CAS | Undisclosed | Less than 500 | Positive | -1.5 |  |  |
|  | NO_CAS | Undisclosed | Less than 500 | Positive | -9.8 |  |  |
|  | NO_CAS | Undisclosed | Less than 500 | Negative | 12.9 |  |  |
|  | NO_CAS | Undisclosed | Less than 500 | Negative | 18.0 |  |  |
|  | NO_CAS | Undisclosed | Less than 500 | Positive | -7.7 |  |  |
|  | \| 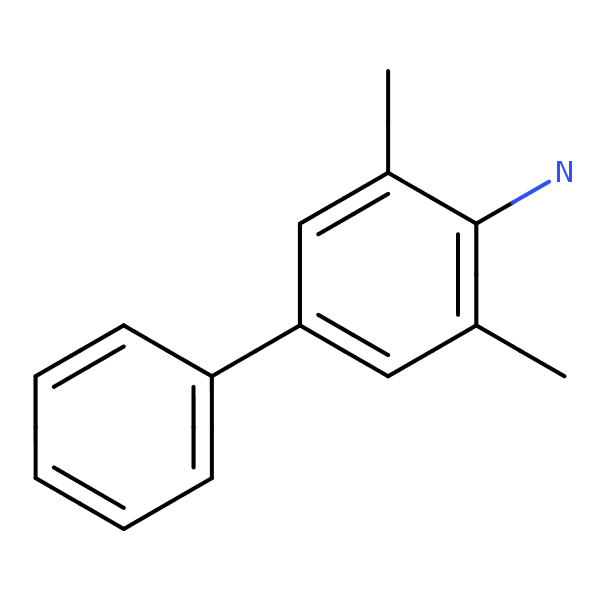NO_CAS \| \| --- \| | Undisclosed | Greater than 500 | Negative | 11.2 |  |  |
|  | NO_CAS | Undisclosed | Less than 500 | Negative | -4.4 |  |  |
|  | NO_CAS | Undisclosed | Less than 500 | Positive | -6.2 |  |  |
|  | NO_CAS | Undisclosed | Less than 500 | Negative | 11.5 |  |  |
|  | NO_CAS | Undisclosed | Greater than 500 | Negative | -14.2 | Two or more amines detected. The first site is used. |  |
|  | NO_CAS | Undisclosed | Less than 500 | Positive | -20.9 |  |  |
|  | NO_CAS | Undisclosed | Less than 500 | Positive | -2.0 |  |  |
|  | NO_CAS | Undisclosed | Less than 500 | Negative | 21.3 |  |  |
|  | NO_CAS | Undisclosed | Less than 500 | Negative | -15.9 |  |  |
|  | NO_CAS | Undisclosed | Greater than 500 | Negative | 29.7 |  |  |
|  | \| 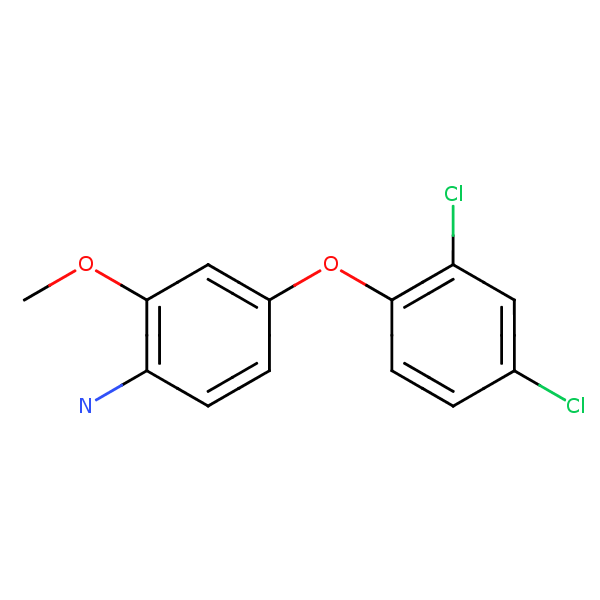NO_CAS \| \| --- \| | Undisclosed | Less than 500 | Negative | 18.4 |  |  |
|  | NO_CAS | Undisclosed | Less than 500 | Negative | -28.1 |  |  |
|  | NO_CAS | Undisclosed | Less than 500 | Positive | 14.4 |  |  |
|  | NO_CAS | Undisclosed | Greater than 500 | Negative | -0.2 | Two or more amines detected. The first site is used. |  |
|  | NO_CAS | Undisclosed | Greater than 500 | Negative | -6.2 |  |  |
|  | 53814-79-2 | C1CCC(CC1)OC(C2=CC(=C4C(=C2N)C(C3=CC=CC=C3C4=O)=O)N(=O)=O)=O | Less than 500 | Positive | 36.3 |  |  |
|  | NO_CAS | Undisclosed | Less than 500 | Negative | 4.2 |  |  |
|  | NO_CAS | Undisclosed | Greater than 500 | Negative | -12.2 |  |  |
|  | NO_CAS | Undisclosed | Less than 500 | Negative | -11.4 | Ring Opening |  |
|  | NO_CAS | Undisclosed | Less than 500 | Negative | -2.8 |  |  |
|  | NO_CAS | Undisclosed | Less than 500 | Negative | 11.8 |  |  |
|  | NO_CAS | COC1=C(C=CC(=C1F)F)N | Less than 500 | Positive | -6.9 |  |  |
|  | NO_CAS | Undisclosed | Less than 500 | Negative | -2.6 |  |  |
|  | NO_CAS | Undisclosed | Less than 500 | Negative | -3.5 |  |  |
|  | NO_CAS | Undisclosed | Less than 500 | Negative | 6.5 |  |  |
|  | NO_CAS | Undisclosed | Less than 500 | Negative | -7.5 | Ring Opening |  |
|  | NO_CAS | Undisclosed | Less than 500 | Negative | 4.7 |  |  |
|  | 1575-37-7 | C1=CC(=C(C=C1Br)N)N | Less than 500 | Positive | -28.2 | Two or more amines detected. The first site is used. |  |
|  | NO_CAS | Undisclosed | Less than 500 | Negative | -5.9 | Two or more amines detected. The first site is used. |  |
|  | NO_CAS | Undisclosed | Less than 500 | Positive | -9.6 |  |  |
|  | NO_CAS | CNS(C3=CC=C1C(C=CC(=C1N=NC=2C=CC(=CC=2O)N(=O)=O)N)=C3)(=O)=O | Less than 500 | Positive | -6.2 |  |  |
|  | NO_CAS | CCC(C)(C)C1=CC=C(C=C1)OCC(=NNC2=CC=C(C=C2)N)O | Less than 500 | Positive | -23.3 |  |  |
|  | NO_CAS | Undisclosed | Greater than 500 | Positive | -0.3 |  |  |
|  | NO_CAS | Undisclosed | Less than 500 | Negative | -15.1 | Two or more amines detected. The first site is used. |  |
|  | NO_CAS | COC=1C=CC(=CC=1N)C2=CC=CC=C2 | Less than 500 | Positive | -17.8 |  |  |
|  | NO_CAS | Undisclosed | Less than 500 | Negative | -15.3 |  |  |
|  | NO_CAS | Undisclosed | Greater than 500 | Negative | 21.4 |  |  |
|  | NO_CAS | Undisclosed | Less than 500 | Negative | 11.2 |  |  |
|  | NO_CAS | Undisclosed | Less than 500 | Negative | 32.5 |  |  |
|  | 14235-81-5 | C#CC1=CC=C(C=C1)N | Less than 500 | Positive | -5.9 |  |  |
|  | NO_CAS | Undisclosed | Less than 500 | Negative | -12.2 | Two or more amines detected. The first site is used. |  |
|  | NO_CAS | Undisclosed | Less than 500 | Negative | 26.4 |  |  |
|  | NO_CAS | Undisclosed | Greater than 500 | Negative | 25.7 |  |  |
|  | NO_CAS | Undisclosed | Greater than 500 | Negative | 57.1 | Ring Opening |  |
|  | NO_CAS | Undisclosed | Greater than 500 | Positive | -3.2 |  |  |
|  | NO_CAS | Undisclosed | Less than 500 | Positive | -19.6 |  |  |
|  | NO_CAS | Undisclosed | Less than 500 | Negative | -34.9 |  |  |
|  | NO_CAS | Undisclosed | Less than 500 | Negative | -4.5 |  |  |
|  | NO_CAS | C=1C(C#N)=C(N)SC=1N(=O)=O | Less than 500 | Positive | 17.4 |  |  |
|  | NO_CAS | Undisclosed | Less than 500 | Negative | 15.0 |  |  |
|  | NO_CAS | Undisclosed | Less than 500 | Negative | -1.0 |  |  |
|  | NO_CAS | C1=C(C(=CC(=C1N)O)O)N | Less than 500 | Positive | -22.6 | Two or more amines detected. The first site is used. |  |
|  | NO_CAS | Undisclosed | Less than 500 | Negative | 6.6 |  |  |
|  | NO_CAS | Undisclosed | Less than 500 | Negative | 19.9 |  |  |
|  | NO_CAS | Undisclosed | Less than 500 | Negative | -0.3 |  |  |
|  | NO_CAS | Undisclosed | Less than 500 | Positive | -15.7 |  |  |
|  | NO_CAS | Undisclosed | Less than 500 | Positive | -17.4 |  |  |
|  | NO_CAS | Undisclosed | Less than 500 | Negative | -24.4 |  |  |
|  | NO_CAS | Undisclosed | Less than 500 | Negative | -4.6 |  |  |
|  | NO_CAS | Undisclosed | Greater than 500 | Negative | -30.3 |  |  |
|  | NO_CAS | Undisclosed | Less than 500 | Positive | 10.7 |  |  |
|  | NO_CAS | Undisclosed | Greater than 500 | Negative | 16.2 | Two or more amines detected. The first site is used. |  |
|  | NO_CAS | Undisclosed | Less than 500 | Negative | -3.2 |  |  |
|  | 55011-44-4 | CC2=CC=3C=1C=CC(=C(C)C=1S(C=3C=C2N)(=O)=O)N | Less than 500 | Positive | -19.7 | Two or more amines detected. The first site is used. |  |
|  | \| 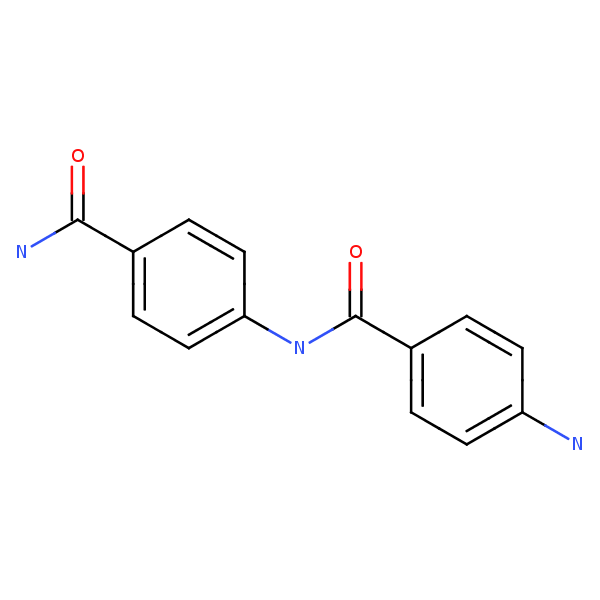757251-54-0 \| \| --- \| | C1=CC(=C(C=C1OC=2C=CN=C(C=2)C(N)=O)F)N | Less than 500 | Positive | -10.3 |  |  |
|  | NO_CAS | Undisclosed | Less than 500 | Negative | 7.7 |  |  |
|  | NO_CAS | Undisclosed | Less than 500 | Negative | -28.4 |  |  |
|  | NO_CAS | Undisclosed | Greater than 500 | Negative | -9.2 | Two or more amines detected. The first site is used. |  |
|  | NO_CAS | Undisclosed | Less than 500 | Negative | -1.6 |  |  |
|  | NO_CAS | Undisclosed | Less than 500 | Negative | 10.9 |  |  |
|  | NO_CAS | Undisclosed | Less than 500 | Negative | -17.0 |  |  |
|  | NO_CAS | Undisclosed | Greater than 500 | Negative | -9.3 | Two or more amines detected. The first site is used. |  |
|  | NO_CAS | Undisclosed | Less than 500 | Negative | -16.9 |  |  |
|  | 89466-18-2 | COC1=C(C=CC(Br)=N1)N | Less than 500 | Positive | -10.4 |  |  |
|  | 91215-79-1 | CCN(C(C)C)C1=CC=C(C=C1)N | Less than 500 | Positive | -36.5 |  |  |
|  | NO_CAS | Undisclosed | Less than 500 | Negative | -18.2 |  |  |
|  | NO_CAS | Undisclosed | Less than 500 | Negative | -13.3 |  |  |
|  | NO_CAS | Undisclosed | Greater than 500 | Positive | -13.0 |  |  |
|  | NO_CAS | Undisclosed | Less than 500 | Negative | -17.9 |  |  |
|  | NO_CAS | Undisclosed | Less than 500 | Negative | 4.5 |  |  |
|  | NO_CAS | Undisclosed | Less than 500 | Negative | -12.0 |  |  |
|  | NO_CAS | CCN(CC)C1=C(C=CC(C)=N1)N | Less than 500 | Positive | -25.5 |  |  |
|  | 100953-52-4 | C1=CC=C(C(=C1)N)NC2=CC=C(C=C2)Br | Less than 500 | Positive | -27.3 |  |  |
|  | NO_CAS | Undisclosed | Greater than 500 | Negative | -7.4 |  |  |
|  | NO_CAS | Undisclosed | Less than 500 | Positive | -28.1 | Two or more amines detected. The first site is used. |  |
|  | NO_CAS | Undisclosed | Less than 500 | Negative | -8.6 |  |  |
|  | NO_CAS | Undisclosed | Greater than 500 | Negative | -19.9 |  |  |
|  | NO_CAS | Undisclosed | Less than 500 | Negative | -6.1 | Two or more amines detected. The first site is used. |  |
|  | NO_CAS | Undisclosed | Less than 500 | Negative | -10.0 | Two or more amines detected. The first site is used. |  |
|  | NO_CAS | Undisclosed | Less than 500 | Negative | 0.5 |  |  |
|  | NO_CAS | Undisclosed | Less than 500 | Positive | -0.1 | Ring Opening |  |
|  | NO_CAS | Undisclosed | Less than 500 | Negative | -6.9 | Ring Opening |  |
|  | NO_CAS | Undisclosed | Less than 500 | Negative | 0.5 |  |  |
|  | NO_CAS | Undisclosed | Less than 500 | Positive | 2.2 | Two or more amines detected. The first site is used. |  |
|  | NO_CAS | Undisclosed | Less than 500 | Negative | 43.3 |  |  |
|  | NO_CAS | Undisclosed | Less than 500 | Positive | -8.4 |  |  |
|  | NO_CAS | Undisclosed | Less than 500 | Negative | -17.3 | Two or more amines detected. The first site is used. |  |
|  | NO_CAS | Undisclosed | Less than 500 | Negative | 28.3 |  |  |
|  | NO_CAS | Undisclosed | Less than 500 | Negative | -8.3 |  |  |
|  | NO_CAS | Undisclosed | Greater than 500 | Negative | -21.6 |  |  |
|  | 4194-40-5 | C1=CC(=C(C=C1C=2C=CC(=C(C=2)N)O)N)O | Less than 500 | Positive | -14.4 | Two or more amines detected. The first site is used. |  |
|  | NO_CAS | Undisclosed | Less than 500 | Negative | -8.9 |  |  |
|  | NO_CAS | Undisclosed | Less than 500 | Positive | -19.8 |  |  |
|  | NO_CAS | Undisclosed | Less than 500 | Negative | 0.7 |  |  |
|  | NO_CAS | Undisclosed | Less than 500 | Positive | -15.1 | Two or more amines detected. The first site is used. |  |
|  | NO_CAS | Undisclosed | Less than 500 | Negative | 3.8 |  |  |
|  | 89923-62-6 | CCC(=NC1=CC=CC(=C1)NC2=CC(=C(C4=C2C(C3=CC=CC=C3C4=O)=O)N)S(O)(=O)=O)O | Less than 500 | Positive | -16.7 |  |  |
|  | NO_CAS | Undisclosed | Less than 500 | Negative | 4.3 |  |  |
|  | NO_CAS | Undisclosed | Less than 500 | Negative | -7.6 | Ring Opening |  |
|  | NO_CAS | Undisclosed | Less than 500 | Negative | 0.4 | Two or more amines detected. The first site is used. |  |
|  | NO_CAS | Undisclosed | Greater than 500 | Negative | -0.1 | Two or more amines detected. The first site is used. |  |
|  | NO_CAS | Undisclosed | Less than 500 | Negative | -33.7 |  |  |
|  | NO_CAS | Undisclosed | Less than 500 | Positive | 47.0 |  |  |
|  | NO_CAS | Undisclosed | Less than 500 | Positive | -19.8 | Two or more amines detected. The first site is used. |  |
|  | NO_CAS | Undisclosed | Less than 500 | Negative | 30.1 |  |  |
|  | NO_CAS | Undisclosed | Less than 500 | Negative | 8.4 |  |  |
|  | NO_CAS | Undisclosed | Less than 500 | Negative | -24.9 |  |  |
|  | NO_CAS | Undisclosed | Less than 500 | Negative | -15.9 |  |  |
|  | NO_CAS | Undisclosed | Less than 500 | Negative | 24.5 | Two or more amines detected. The first site is used. |  |
|  | NO_CAS | Undisclosed | Less than 500 | Negative | -30.0 |  |  |
|  | NO_CAS | Undisclosed | Less than 500 | Negative | -11.8 | Ring Opening |  |
|  | NO_CAS | Undisclosed | Less than 500 | Negative | -3.2 |  |  |
|  | NO_CAS | Undisclosed | Less than 500 | Negative | -3.2 | Ring Opening |  |
|  | NO_CAS | Undisclosed | Less than 500 | Negative | -11.0 | Ring Opening |  |
|  | NO_CAS | Undisclosed | Greater than 500 | Negative | -1.0 |  |  |
|  | NO_CAS | Undisclosed | Less than 500 | Positive | -0.5 |  |  |
|  | 27048-04-0 | C1=CC(Cl)=NC(=C1N(=O)=O)N | Less than 500 | Positive | 43.4 |  |  |
|  | 6976-04-1 | C=1C=C(C=CC=1N)S | Less than 500 | Positive | -15.9 |  |  |
|  | NO_CAS | Undisclosed | Greater than 500 | Negative | -3.9 |  |  |
|  | NO_CAS | Undisclosed | Greater than 500 | Negative | 58.0 |  |  |
|  | NO_CAS | Undisclosed | Less than 500 | Negative | 12.9 |  |  |
|  | NO_CAS | Undisclosed | Less than 500 | Negative | -35.1 |  |  |
|  | NO_CAS | Undisclosed | Less than 500 | Positive | -22.0 |  |  |
|  | NO_CAS | Undisclosed | Greater than 500 | Negative | -13.7 |  |  |
|  | NO_CAS | Undisclosed | Less than 500 | Negative | -24.6 |  |  |
|  | NO_CAS | Undisclosed | Less than 500 | Negative | -17.5 |  |  |
|  | NO_CAS | Undisclosed | Less than 500 | Positive | -23.5 | Two or more amines detected. The first site is used. |  |
|  | NO_CAS | Undisclosed | Less than 500 | Negative | -19.7 | Ring Opening |  |
|  | NO_CAS | Undisclosed | Less than 500 | Negative | 1.4 |  |  |
|  | NO_CAS | Undisclosed | Less than 500 | Negative | -27.5 | Two or more amines detected. The first site is used. |  |
|  | NO_CAS | Undisclosed | Less than 500 | Negative | -0.5 |  |  |
|  | NO_CAS | Undisclosed | Less than 500 | Negative | 1.2 |  |  |
|  | NO_CAS | Undisclosed | Less than 500 | Negative | -26.5 | Two or more amines detected. The first site is used. |  |
|  | NO_CAS | Undisclosed | Greater than 500 | Negative | 0.3 |  |  |
|  | NO_CAS | Undisclosed | Greater than 500 | Negative | -12.5 |  |  |
|  | 63402-26-6 | C=1C=C(C=CC=1N)NN=CO | Less than 500 | Positive | -18.0 |  |  |
|  | NO_CAS | Undisclosed | Less than 500 | Negative | -13.1 |  |  |
|  | NO_CAS | Undisclosed | Less than 500 | Negative | 8.6 |  |  |
|  | NO_CAS | Undisclosed | Less than 500 | Negative | -0.6 |  |  |
|  | NO_CAS | CC1=CC(=C(C(=N1)SC)N)SC | Less than 500 | Positive | -25.5 |  |  |
|  | NO_CAS | Undisclosed | Less than 500 | Negative | -0.4 |  |  |
|  | NO_CAS | Undisclosed | Less than 500 | Negative | 25.0 |  |  |
|  | NO_CAS | Undisclosed | Less than 500 | Negative | 51.6 |  |  |
|  | NO_CAS | Undisclosed | Less than 500 | Negative | -11.3 |  |  |
|  | NO_CAS | Undisclosed | Less than 500 | Negative | -9.2 |  |  |
|  | NO_CAS | Undisclosed | Less than 500 | Negative | -20.2 |  |  |
|  | NO_CAS | Undisclosed | Less than 500 | Negative | -15.5 |  |  |
|  | NO_CAS | Undisclosed | Less than 500 | Negative | 11.9 |  |  |
|  | NO_CAS | Undisclosed | Less than 500 | Negative | -26.0 |  |  |
|  | NO_CAS | Undisclosed | Less than 500 | Positive | 26.2 |  |  |
|  | NO_CAS | Undisclosed | Less than 500 | Negative | -9.7 | Ring Opening |  |
|  | NO_CAS | Undisclosed | Less than 500 | Negative | 10.7 |  |  |
|  | NO_CAS | C1=C(C(=CC2=C1OCC(=N2)O)N)F | Less than 500 | Positive | -6.4 |  |  |
|  | NO_CAS | Undisclosed | Less than 500 | Negative | 14.8 | Ring Opening |  |
|  | NO_CAS | Undisclosed | Less than 500 | Negative | 5.8 |  |  |
|  | NO_CAS | Undisclosed | Less than 500 | Negative | -13.0 |  |  |
|  | NO_CAS | Undisclosed | Less than 500 | Negative | -13.4 |  |  |
|  | NO_CAS | Undisclosed | Less than 500 | Negative | -37.3 |  |  |
|  | NO_CAS | Undisclosed | Less than 500 | Negative | -21.4 |  |  |
|  | NO_CAS | Undisclosed | Less than 500 | Negative | -13.5 |  |  |
|  | NO_CAS | Undisclosed | Less than 500 | Negative | -47.0 | Two or more amines detected. The first site is used. |  |
|  | NO_CAS | Undisclosed | Less than 500 | Negative | -18.0 | Two or more amines detected. The first site is used. |  |
|  | NO_CAS | Undisclosed | Greater than 500 | Positive | -22.7 |  |  |
|  | NO_CAS | Undisclosed | Less than 500 | Negative | -21.2 |  |  |
|  | NO_CAS | Undisclosed | Less than 500 | Negative | -2.2 |  |  |
|  | NO_CAS | Undisclosed | Less than 500 | Negative | 6.9 |  |  |
|  | NO_CAS | C1(=C(C(=C(C(=C1I)N)I)C(Cl)=O)I)C(Cl)=O | Greater than 500 | Positive | 25.1 |  |  |
|  | NO_CAS | Undisclosed | Less than 500 | Negative | -4.2 |  |  |
|  | NO_CAS | Undisclosed | Less than 500 | Negative | -0.4 |  |  |
|  | NO_CAS | Undisclosed | Greater than 500 | Positive | -15.5 |  |  |
|  | NO_CAS | Undisclosed | Less than 500 | Positive | -2.9 |  |  |
|  | NO_CAS | Undisclosed | Less than 500 | Negative | -4.9 |  |  |
|  | NO_CAS | Undisclosed | Less than 500 | Negative | -13.6 |  |  |
|  | NO_CAS | Undisclosed | Less than 500 | Negative | 34.0 |  |  |
|  | NO_CAS | Undisclosed | Less than 500 | Negative | 9.9 |  |  |
|  | NO_CAS | Undisclosed | Less than 500 | Negative | -31.8 |  |  |
|  | NO_CAS | Undisclosed | Less than 500 | Positive | -48.3 |  |  |
|  | NO_CAS | Undisclosed | Less than 500 | Positive | -11.1 |  |  |
|  | NO_CAS | Undisclosed | Greater than 500 | Negative | 2.3 |  |  |
|  | 417722-93-1 | COC=1C=C2C(=CC=1C(=N)O)C(=CC=N2)OC=3C=CC(=C(C=3)Cl)N | Less than 500 | Positive | -9.9 |  |  |
|  | NO_CAS | Undisclosed | Less than 500 | Negative | -5.7 | Ring Opening |  |
|  | NO_CAS | Undisclosed | Less than 500 | Negative | -0.8 |  |  |
|  | NO_CAS | Undisclosed | Less than 500 | Negative | -22.2 |  |  |
|  | 4097-22-7 | C3CC(=N2=C[N-]C1=C(N)N=CN=C12)[O+]=C3CO | Less than 500 | Positive | 0.4 |  |  |
|  | NO_CAS | Undisclosed | Less than 500 | Negative | -2.6 | Ring Opening |  |
|  | NO_CAS | Undisclosed | Less than 500 | Negative | -39.7 |  |  |
|  | NO_CAS | Undisclosed | Less than 500 | Positive | -15.1 |  |  |
|  | NO_CAS | Undisclosed | Less than 500 | Negative | -46.4 | Two or more amines detected. The first site is used. |  |
|  | NO_CAS | Undisclosed | Less than 500 | Negative | -6.4 | Ring Opening |  |
|  | NO_CAS | Undisclosed | Less than 500 | Positive | -11.5 |  |  |
|  | NO_CAS | Undisclosed | Less than 500 | Positive | -35.7 |  |  |
|  | NO_CAS | Undisclosed | Greater than 500 | Negative | -4.3 |  |  |
|  | 2479-46-1 | C1=CC(=CC(=C1)OC2=CC=C(C=C2)N)OC3=CC=C(C=C3)N | Less than 500 | Positive | -19.9 | Two or more amines detected. The first site is used. |  |
|  | NO_CAS | Undisclosed | Less than 500 | Negative | -3.3 |  |  |
|  | NO_CAS | Undisclosed | Less than 500 | Negative | -3.9 | Two or more amines detected. The first site is used. |  |
|  | NO_CAS | COC1=CC(=C(C=C1N)OC)N=NC2=CC=C(C=C2)C(O)=O | Less than 500 | Positive | -17.7 |  |  |
|  | NO_CAS | Undisclosed | Less than 500 | Negative | 6.8 |  |  |
|  | NO_CAS | Undisclosed | Less than 500 | Negative | 32.6 |  |  |
|  | NO_CAS | Undisclosed | Less than 500 | Negative | 10.8 |  |  |
|  | 7621-86-5 | C=1C=C(C=CC=1C=3NC=2C=CC(=CC=2N=3)N)N | Less than 500 | Positive | -21.8 | Two or more amines detected. The first site is used. |  |
|  | NO_CAS | Undisclosed | Less than 500 | Negative | 55.3 | Ring Opening |  |
|  | NO_CAS | Undisclosed | Less than 500 | Negative | -14.2 |  |  |
|  | NO_CAS | Undisclosed | Less than 500 | Negative | -17.0 |  |  |
|  | NO_CAS | Undisclosed | Less than 500 | Negative | -21.9 |  |  |
|  | NO_CAS | Undisclosed | Less than 500 | Negative | -4.3 | Ring Opening |  |
|  | NO_CAS | Undisclosed | Less than 500 | Negative | 48.5 | Two or more amines detected. The first site is used. |  |
|  | NO_CAS | Undisclosed | Less than 500 | Positive | -11.0 |  |  |
|  | NO_CAS | Undisclosed | Greater than 500 | Negative | -2.3 |  |  |
|  | NO_CAS | Undisclosed | Less than 500 | Negative | 9.6 |  |  |
|  | NO_CAS | Undisclosed | Less than 500 | Negative | -13.5 |  |  |
|  | NO_CAS | Undisclosed | Less than 500 | Positive | -3.8 |  |  |
|  | NO_CAS | Undisclosed | Less than 500 | Negative | -19.8 | Two or more amines detected. The first site is used. |  |
|  | 66422-95-5 | C1=CC(=C(C=C1N)N)OCCO | Less than 500 | Positive | -14.6 | Two or more amines detected. The first site is used. |  |
|  | NO_CAS | Undisclosed | Less than 500 | Positive | -14.9 | Ring Opening |  |
|  | NO_CAS | Undisclosed | Greater than 500 | Negative | -23.5 |  |  |
|  | NO_CAS | Undisclosed | Greater than 500 | Negative | 21.9 |  |  |
|  | NO_CAS | Undisclosed | Less than 500 | Negative | -11.8 |  |  |
|  | NO_CAS | Undisclosed | Less than 500 | Negative | -6.5 |  |  |
|  | NO_CAS | Undisclosed | Greater than 500 | Negative | -13.6 | Ring Opening |  |
|  | NO_CAS | Undisclosed | Less than 500 | Negative | 5.1 |  |  |
|  | NO_CAS | Undisclosed | Less than 500 | Positive | -10.4 | Two or more amines detected. The first site is used. |  |
|  | NO_CAS | Undisclosed | Less than 500 | Negative | -0.5 |  |  |
|  | NO_CAS | Undisclosed | Less than 500 | Positive | -8.1 |  |  |
|  | NO_CAS | Undisclosed | Less than 500 | Positive | 10.0 |  |  |
|  | NO_CAS | Undisclosed | Less than 500 | Negative | 4.4 |  |  |
|  | NO_CAS | Undisclosed | Less than 500 | Negative | -10.3 | Two or more amines detected. The first site is used. |  |
|  | NO_CAS | Undisclosed | Greater than 500 | Negative | -5.4 |  |  |
|  | NO_CAS | Undisclosed | Less than 500 | Positive | 0.0 |  |  |
|  | NO_CAS | Undisclosed | Less than 500 | Negative | 12.0 |  |  |
|  | NO_CAS | Undisclosed | Less than 500 | Negative | 8.8 |  |  |
|  | NO_CAS | Undisclosed | Greater than 500 | Negative | -14.1 | Two or more amines detected. The first site is used. |  |
|  | NO_CAS | Undisclosed | Less than 500 | Positive | 52.9 | Two or more amines detected. The first site is used. |  |
|  | NO_CAS | Undisclosed | Less than 500 | Negative | -33.1 |  |  |
|  | NO_CAS | Undisclosed | Less than 500 | Negative | -21.4 |  |  |
|  | NO_CAS | Undisclosed | Greater than 500 | Negative | -9.8 |  |  |
|  | NO_CAS | Undisclosed | Less than 500 | Negative | -22.3 | Two or more amines detected. The first site is used. |  |
|  | NO_CAS | Undisclosed | Less than 500 | Negative | -31.4 |  |  |
|  | NO_CAS | Undisclosed | Less than 500 | Negative | -1.6 |  |  |
|  | NO_CAS | Undisclosed | Less than 500 | Negative | -3.1 |  |  |
|  | NO_CAS | Undisclosed | Less than 500 | Negative | -10.3 | Ring Opening |  |
|  | NO_CAS | Undisclosed | Less than 500 | Negative | -16.7 | Ring Opening |  |
|  | NO_CAS | Undisclosed | Less than 500 | Negative | 35.9 |  |  |
|  | NO_CAS | Undisclosed | Less than 500 | Negative | -2.4 |  |  |
|  | NO_CAS | Undisclosed | Less than 500 | Negative | -19.1 |  |  |
|  | NO_CAS | Undisclosed | Less than 500 | Negative | -10.1 | Ring Opening |  |
|  | NO_CAS | Undisclosed | Less than 500 | Negative | 3.2 |  |  |
|  | NO_CAS | Undisclosed | Less than 500 | Negative | -19.3 |  |  |
|  | NO_CAS | Undisclosed | Less than 500 | Negative | -11.5 | Ring Opening |  |
|  | NO_CAS | Undisclosed | Less than 500 | Negative | -5.6 |  |  |
|  | NO_CAS | Undisclosed | Greater than 500 | Negative | -8.1 |  |  |
|  | NO_CAS | C=1C=C(C=CC=1C=2C=CC(=C(C=2)N)O)Br | Less than 500 | Positive | -12.1 |  |  |
|  | NO_CAS | Undisclosed | Less than 500 | Positive | -17.9 |  |  |
|  | NO_CAS | Undisclosed | Less than 500 | Negative | -19.9 |  |  |
|  | NO_CAS | Undisclosed | Less than 500 | Negative | -26.2 |  |  |
|  | NO_CAS | COC1=CC=C(C=C1)N=NC2=CC=C(C=C2)N | Less than 500 | Positive | -13.5 |  |  |
|  | NO_CAS | Undisclosed | Less than 500 | Negative | -15.7 |  |  |
|  | NO_CAS | Undisclosed | Less than 500 | Negative | 1.1 |  |  |
|  | NO_CAS | Undisclosed | Less than 500 | Negative | -19.1 | Two or more amines detected. The first site is used. |  |
|  | 57131-19-8 | C1=CC=C(C=C1)NN=C3C(=CC2=CC(=C(C(=C2C3=O)N)N=NC4=CC=C(C=C4)C(=NC5=CC=C(C=C5)N=NC=6C=CC(=CC=6N)N)O)S(O)(=O)=O)S(O)(=O)=O | Greater than 500 | Positive | -22.6 | Two or more amines detected. The first site is used. |  |
|  | NO_CAS | Undisclosed | Less than 500 | Positive | -7.7 |  |  |
|  | NO_CAS | Undisclosed | Less than 500 | Positive | 61.4 |  |  |
|  | NO_CAS | Undisclosed | Less than 500 | Positive | -10.0 | Two or more amines detected. The first site is used. |  |
|  | NO_CAS | Undisclosed | Less than 500 | Negative | -6.2 |  |  |
|  | NO_CAS | Undisclosed | Less than 500 | Negative | 81.6 |  |  |
|  | NO_CAS | Undisclosed | Less than 500 | Negative | 22.6 |  |  |
|  | NO_CAS | Undisclosed | Less than 500 | Negative | -37.4 |  |  |
|  | NO_CAS | Undisclosed | Less than 500 | Negative | 13.1 |  |  |
|  | NO_CAS | Undisclosed | Less than 500 | Negative | 14.7 |  |  |
|  | NO_CAS | Undisclosed | Less than 500 | Negative | -10.4 | Two or more amines detected. The first site is used. |  |
|  | NO_CAS | Undisclosed | Greater than 500 | Negative | -7.0 |  |  |
|  | NO_CAS | Undisclosed | Less than 500 | Negative | 50.3 | Two or more amines detected. The first site is used. |  |
|  | NO_CAS | Undisclosed | Less than 500 | Negative | 1.0 | Two or more amines detected. The first site is used. |  |
|  | NO_CAS | Undisclosed | Greater than 500 | Negative | 6.9 | Two or more amines detected. The first site is used. |  |
|  | NO_CAS | Undisclosed | Less than 500 | Negative | -1.1 | Two or more amines detected. The first site is used. |  |
|  | NO_CAS | Undisclosed | Less than 500 | Negative | -22.5 |  |  |
|  | NO_CAS | Undisclosed | Less than 500 | Negative | -9.1 |  |  |
|  | NO_CAS | Undisclosed | Less than 500 | Negative | 50.8 | Two or more amines detected. The first site is used. |  |
|  | NO_CAS | Undisclosed | Less than 500 | Positive | -8.7 |  |  |
|  | NO_CAS | Undisclosed | Greater than 500 | Positive | 8.5 | Two or more amines detected. The first site is used. |  |
|  | NO_CAS | Undisclosed | Less than 500 | Negative | -10.1 | Ring Opening |  |
|  | NO_CAS | Undisclosed | Less than 500 | Negative | -7.8 | Two or more amines detected. The first site is used. |  |
|  | NO_CAS | Undisclosed | Less than 500 | Positive | -19.1 |  |  |
|  | NO_CAS | Undisclosed | Greater than 500 | Negative | -19.5 |  |  |
|  | NO_CAS | Undisclosed | Less than 500 | Negative | -9.0 |  |  |
|  | NO_CAS | Undisclosed | Less than 500 | Negative | 16.2 |  |  |
|  | NO_CAS | Undisclosed | Less than 500 | Negative | -7.3 |  |  |
|  | NO_CAS | Undisclosed | Greater than 500 | Negative | -16.5 |  |  |
|  | 105757-36-6 | CN2C=CC(=CC=C1C=CC(C=C1)=[NH2+])C=C2 | Less than 500 | Positive | 55.4 |  |  |
|  | NO_CAS | Undisclosed | Less than 500 | Negative | 11.9 |  |  |
|  | NO_CAS | Undisclosed | Less than 500 | Negative | 6.4 |  |  |
|  | NO_CAS | Undisclosed | Less than 500 | Negative | 1.2 |  |  |
|  | NO_CAS | Undisclosed | Less than 500 | Negative | -2.3 |  |  |
|  | NO_CAS | Undisclosed | Less than 500 | Negative | 11.0 |  |  |
|  | NO_CAS | Undisclosed | Greater than 500 | Negative | -8.3 |  |  |
|  | NO_CAS | Undisclosed | Less than 500 | Negative | 4.6 |  |  |
|  | 2011-66-7 | C1=CC=C(C(=C1)C(C=2C=C(C=CC=2N)N(=O)=O)=O)Cl | Less than 500 | Positive | 24.7 |  |  |
|  | NO_CAS | Undisclosed | Less than 500 | Negative | 14.6 |  |  |
|  | NO_CAS | Undisclosed | Less than 500 | Negative | -4.8 |  |  |
|  | NO_CAS | Undisclosed | Less than 500 | Negative | -4.6 |  |  |
|  | NO_CAS | Undisclosed | Less than 500 | Negative | -10.5 | Ring Opening |  |
|  | NO_CAS | Undisclosed | Less than 500 | Negative | 5.4 |  |  |
|  | NO_CAS | Undisclosed | Less than 500 | Negative | -4.3 |  |  |
|  | NO_CAS | Undisclosed | Greater than 500 | Negative | -14.7 |  |  |
|  | NO_CAS | Undisclosed | Greater than 500 | Negative | 72.2 | Two or more amines detected. The first site is used. |  |
|  | NO_CAS | Undisclosed | Less than 500 | Negative | 4.9 |  |  |
|  | NO_CAS | Undisclosed | Less than 500 | Positive | -2.7 |  |  |
|  | NO_CAS | Undisclosed | Greater than 500 | Negative | -15.8 | Two or more amines detected. The first site is used. |  |
|  | NO_CAS | Undisclosed | Less than 500 | Negative | 1.0 |  |  |
|  | NO_CAS | Undisclosed | Less than 500 | Positive | -22.5 |  |  |
|  | NO_CAS | Undisclosed | Less than 500 | Negative | -25.1 |  |  |
|  | NO_CAS | Undisclosed | Less than 500 | Negative | -11.2 |  |  |
|  | NO_CAS | Undisclosed | Less than 500 | Negative | 22.9 |  |  |
|  | NO_CAS | Undisclosed | Greater than 500 | Negative | 16.1 |  |  |
|  | NO_CAS | Undisclosed | Less than 500 | Negative | -23.4 |  |  |
|  | NO_CAS | Undisclosed | Greater than 500 | Negative | -28.5 |  |  |
|  | NO_CAS | Undisclosed | Less than 500 | Negative | -8.9 |  |  |
|  | NO_CAS | Undisclosed | Less than 500 | Negative | 32.8 | Ring Opening |  |
|  | NO_CAS | Undisclosed | Greater than 500 | Negative | 17.7 |  |  |
|  | NO_CAS | Undisclosed | Less than 500 | Negative | 22.5 |  |  |
|  | NO_CAS | Undisclosed | Less than 500 | Negative | -15.7 |  |  |
|  | NO_CAS | Undisclosed | Greater than 500 | Negative | 22.5 | Two or more amines detected. The first site is used. |  |
|  | NO_CAS | Undisclosed | Less than 500 | Negative | 32.6 |  |  |
|  | NO_CAS | Undisclosed | Greater than 500 | Negative | -3.9 | Two or more amines detected. The first site is used. |  |
|  | NO_CAS | Undisclosed | Less than 500 | Negative | -9.9 |  |  |
|  | NO_CAS | Undisclosed | Less than 500 | Negative | -6.5 |  |  |
|  | NO_CAS | Undisclosed | Less than 500 | Negative | -26.6 |  |  |
|  | NO_CAS | Undisclosed | Less than 500 | Negative | -6.4 | Ring Opening |  |
|  | NO_CAS | Undisclosed | Greater than 500 | Negative | -9.4 | Ring Opening |  |
|  | \| 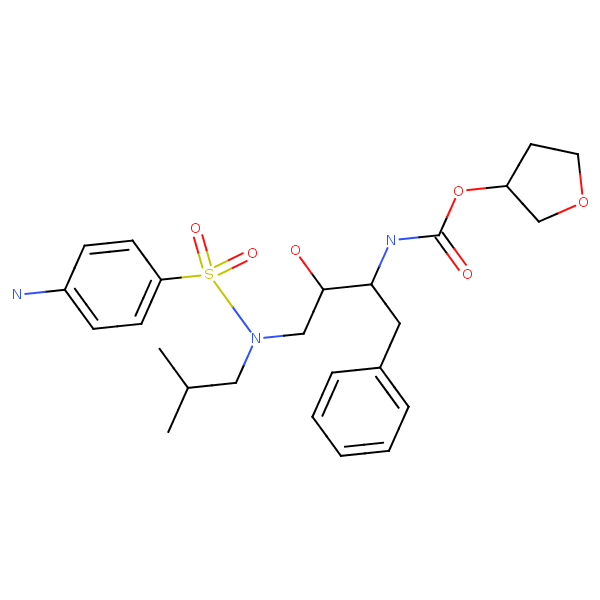NO_CAS \| \| --- \| | Undisclosed | Less than 500 | Negative | -11.4 | Two or more amines detected. The first site is used. |  |
|  | NO_CAS | Undisclosed | Greater than 500 | Negative | 62.7 |  |  |
|  | NO_CAS | Undisclosed | Less than 500 | Negative | -20.0 | Two or more amines detected. The first site is used. |  |
|  | NO_CAS | Undisclosed | Less than 500 | Negative | -17.2 | Two or more amines detected. The first site is used. |  |
|  | NO_CAS | C=1C=C(C=CC=1C2=CSC(=C2C#N)N)N | Less than 500 | Positive | -17.8 | Two or more amines detected. The first site is used. |  |
|  | NO_CAS | Undisclosed | Less than 500 | Negative | -22.5 | Two or more amines detected. The first site is used. |  |
|  | NO_CAS | Undisclosed | Less than 500 | Negative | 0.4 |  |  |
|  | NO_CAS | Undisclosed | Less than 500 | Negative | -38.1 | Ring Opening |  |
|  | NO_CAS | Undisclosed | Less than 500 | Negative | -29.9 |  |  |
|  | NO_CAS | Undisclosed | Less than 500 | Negative | 2.8 |  |  |
|  | NO_CAS | Undisclosed | Less than 500 | Negative | 4.4 |  |  |
|  | NO_CAS | Undisclosed | Greater than 500 | Negative | -13.5 |  |  |
|  | NO_CAS | Undisclosed | Less than 500 | Negative | 22.8 |  |  |
|  | NO_CAS | Undisclosed | Less than 500 | Negative | 17.5 |  |  |
|  | \| 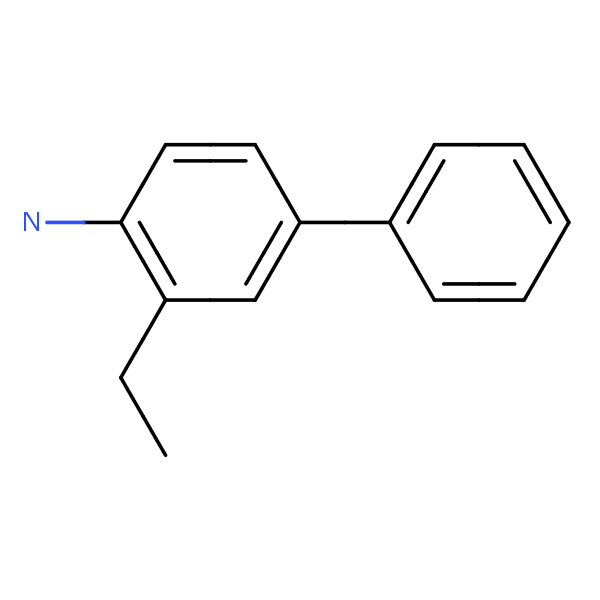NO_CAS \| \| --- \| | Undisclosed | Less than 500 | Negative | -12.1 |  |  |
|  | NO_CAS | Undisclosed | Less than 500 | Negative | 36.1 |  |  |
|  | NO_CAS | Undisclosed | Less than 500 | Positive | 15.8 |  |  |
|  | NO_CAS | Undisclosed | Less than 500 | Negative | 2.1 |  |  |
|  | NO_CAS | Undisclosed | Less than 500 | Negative | -25.1 | Two or more amines detected. The first site is used. |  |
|  | NO_CAS | Undisclosed | Less than 500 | Negative | -8.2 |  |  |
|  | NO_CAS | Undisclosed | Greater than 500 | Negative | -10.8 |  |  |
|  | \| 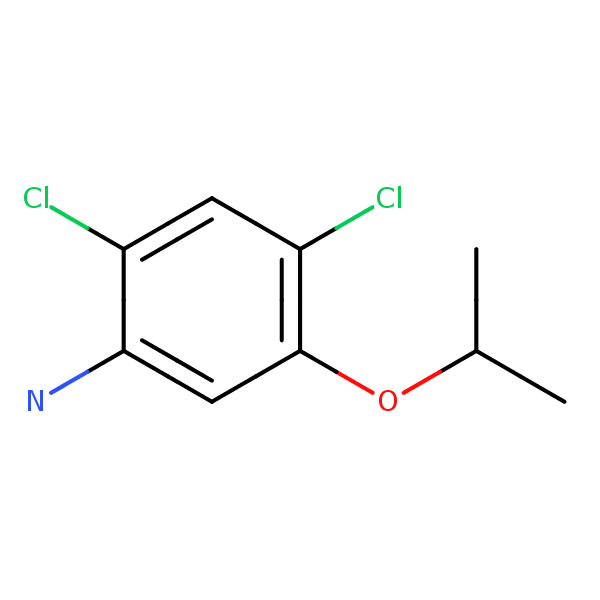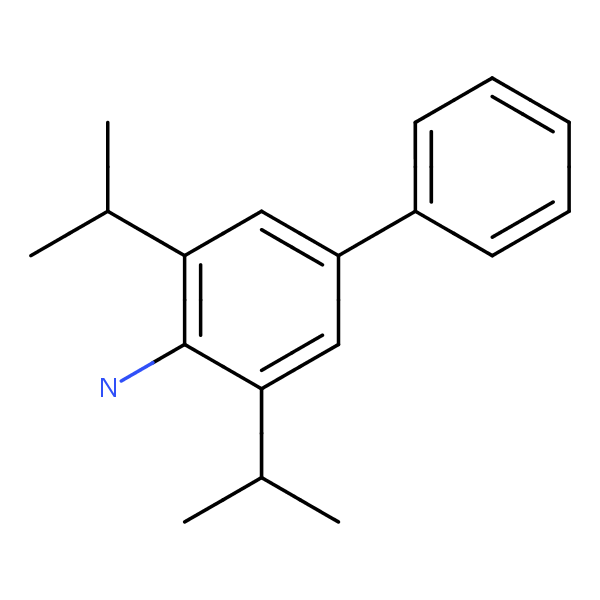NO_CAS \| \| --- \| | Undisclosed | Less than 500 | Negative | 10.1 |  |  |
|  | 41608-64-4 | COC=1C=C(C=CC=1N)C(=O)OC | Less than 500 | Positive | -6.9 |  |  |
|  | NO_CAS | Undisclosed | Less than 500 | Negative | -11.9 |  |  |
|  | 122520-80-3 | CC(C1=CC=C(C=C1)N)=C(C#N)C#N | Less than 500 | Positive | 5.8 |  |  |
|  | NO_CAS | Undisclosed | Less than 500 | Negative | 20.9 |  |  |
|  | NO_CAS | Undisclosed | Less than 500 | Negative | -14.3 | Two or more amines detected. The first site is used. |  |
|  | NO_CAS | Undisclosed | Less than 500 | Negative | -10.8 |  |  |
|  | NO_CAS | Undisclosed | Greater than 500 | Negative | -6.4 |  |  |
|  | NO_CAS | Undisclosed | Less than 500 | Negative | 4.8 |  |  |
|  | NO_CAS | C=CCN(CC=C)C=1C=CC(=CC=1N)N | Less than 500 | Positive | -29.4 | Two or more amines detected. The first site is used. |  |
|  | NO_CAS | Undisclosed | Less than 500 | Negative | -8.6 |  |  |
|  | NO_CAS | Undisclosed | Less than 500 | Negative | 43.4 |  |  |
|  | NO_CAS | Undisclosed | Greater than 500 | Negative | 10.8 |  |  |
|  | NO_CAS | Undisclosed | Greater than 500 | Positive | -19.0 |  |  |
|  | NO_CAS | Undisclosed | Less than 500 | Negative | -5.9 |  |  |
|  | NO_CAS | Undisclosed | Less than 500 | Positive | -5.4 |  |  |
|  | NO_CAS | Undisclosed | Greater than 500 | Negative | -5.4 |  |  |
|  | NO_CAS | Undisclosed | Greater than 500 | Negative | -11.0 |  |  |
|  | NO_CAS | Undisclosed | Less than 500 | Negative | -19.4 | Two or more amines detected. The first site is used. |  |
|  | NO_CAS | Undisclosed | Less than 500 | Positive | -2.4 |  |  |
|  | NO_CAS | Undisclosed | Less than 500 | Negative | 10.3 |  |  |
|  | NO_CAS | Undisclosed | Less than 500 | Negative | -19.3 |  |  |
|  | NO_CAS | Undisclosed | Less than 500 | Negative | 14.2 |  |  |
|  | NO_CAS | Undisclosed | Less than 500 | Negative | 27.7 |  |  |
|  | NO_CAS | Undisclosed | Greater than 500 | Negative | -9.1 |  |  |
|  | NO_CAS | Undisclosed | Less than 500 | Positive | -21.6 |  |  |
|  | NO_CAS | Undisclosed | Less than 500 | Positive | 5.8 |  |  |
|  | NO_CAS | Undisclosed | Less than 500 | Positive | -12.7 |  |  |
|  | NO_CAS | Undisclosed | Less than 500 | Negative | -40.3 |  |  |
|  | NO_CAS | Undisclosed | Less than 500 | Positive | -16.4 | Two or more amines detected. The first site is used. |  |
|  | 129373-04-2 | CC(C)(C)C=1C=CC(=C(C=1)F)N | Less than 500 | Positive | -7.4 |  |  |
|  | NO_CAS | Undisclosed | Less than 500 | Negative | 3.0 |  |  |
|  | NO_CAS | Undisclosed | Less than 500 | Positive | -9.1 | Two or more amines detected. The first site is used. |  |
|  | NO_CAS | Undisclosed | Greater than 500 | Negative | -15.3 |  |  |
|  | NO_CAS | Undisclosed | Less than 500 | Negative | -3.7 |  |  |
|  | NO_CAS | Undisclosed | Less than 500 | Positive | -47.3 |  |  |
|  | NO_CAS | Undisclosed | Less than 500 | Negative | -20.7 |  |  |
|  | NO_CAS | Undisclosed | Less than 500 | Negative | 17.1 |  |  |
|  | NO_CAS | Undisclosed | Less than 500 | Negative | -4.9 |  |  |
|  | NO_CAS | Undisclosed | Less than 500 | Negative | 51.9 | Two or more amines detected. The first site is used. |  |
|  | 29097-01-6 | COC(C=2C=NN(C1=CC=CC=C1)C=2N)=O | Less than 500 | Positive | -4.2 |  |  |
|  | NO_CAS | Undisclosed | Less than 500 | Negative | 11.8 |  |  |

1. McCann J, Choi E, Yamasaki E, Ames BN. Detection of carcinogens as mutagens in the Salmonella/microsome test: assay of 300 chemicals. Proc Natl Acad Sci U S A. 1975;72(12):5135-9.

2. Mortelmans K, Haworth S, Lawlor T, Speck W, Tainer B, Zeiger E. Salmonella Mutagenicity Tests .2. Results from the Testing of 270 Chemicals. Environ Mutagen. 1986;8:1-119.

3. Goggelmann W, Bauchinger M, Kulka U, Schmid E. Genotoxicity of 4-chloro-o-toluidine in Salmonella typhimurium, human lymphocytes and V79 cells. Mutat Res-Genet Tox. 1996;370(1):39-47.

4. Kuglersteigmeier ME, Friederich U, Graf U, Lutz WK, Maier P, Schlatter C. Genotoxicity of Aniline Derivatives in Various Short-Term Tests. Mutat Res. 1989;211(2):279-89.

5. Ahlberg E, Amberg A, Beilke LD, Bower D, Cross KP, Custer L, et al. Extending (Q)SARs to incorporate proprietary knowledge for regulatory purposes: A case study using aromatic amine mutagenicity. Regul Toxicol Pharmacol. 2016;77:1-12.

6. Greene N, Dobo KL, Kenyon MO, Cheung J, Munzner J, Sobol Z, et al. A practical application of two in silico systems for identification of potentially mutagenic impurities. Regul Toxicol Pharmacol. 2015;72(2):335-49.

7. Heng ZC, Ong T, Nath J. In vitro studies on the genotoxicity of 2,4-dichloro-6-nitrophenol ammonium (DCNPA) and its major metabolite. Mutat Res-Genet Tox. 1996;368(2):149-55.

8. Longnecker DS, Curphey TJ, Daniel DS. Mutagenicity of neutral red. Mutat Res. 1977;48(1):109-11.

9. Tanaka K, Ino T, Sawahata T, Marui S, Igaki H, Yashima H. Mutagenicity of N-Acetyl and N,N'-Diacetyl Derivatives of 3 Aromatic-Amines Used as Epoxy-Resin Hardeners. Mutat Res. 1985;143(1-2):11-5.

10. Fukunaga M, Mizuguchi Y, Yielding LW. The Effects of Metabolic-Activation on the Mutagenicity of Aminoacridines. Chem Pharm Bull. 1987;35(2):792-7.

11. Takahashi K, Kaiya T, Kawazoe Y. Studies on Chemical Carcinogens and Mutagens .43. Structure-Mutagenicity Relationship among Aminoquinolines, Aza-Analogs of Naphthylamine, and Their N-Acetyl Derivatives. Mutat Res. 1987;187(4):191-7.

12. Brown JP, Brown RJ. Mutagenesis by 9,10-anthraquinone derivatives and related compounds in Salmonella typhimurium. Mutat Res. 1976;40(3):203-24.

13. Gorrod JW, Ioannides C, Lam SP, Neville S. Mutagenicity Testing of 9-N-Substituted Adenines and Their N-Oxidation Products. Environ Health Persp. 1993;101:21-6.

14. Later DW, Pelroy RA, Stewart DL, McFall T, Booth GM, Lee ML, et al. Microbial mutagenicity of isomeric two-, three-, and four-ring amino polycyclic aromatic hydrocarbons. Environ Mutagen. 1984;6(4):497-515.

15. Marczylo T, Ioannides C. Bioactivation of 6-Aminochrysene by Animal and Human Hepatic Preparations - Contributions of Microsomal and Cytosolic Enzyme-Systems. Mutagenesis. 1994;9(3):233-9.

16. Smith C, Payne V, Doolittle DJ, Debnath AK, Lawlor T, Hansch C. Mutagenic Activity of a Series of Synthetic and Naturally-Occurring Heterocyclic Amines in Salmonella. Mutat Res. 1992;279(1):61-73.

17. Juneja TR, Kaur B, Gupta RL. Mutagenicity of 4-Nitrodiphenyl Thioether-Derived Products and Their Potential Metabolites. Mutat Res. 1991;263(1):13-9.

18. Sinsheimer JE, Hooberman BH, Das SK, Brezzell MD, You Z. The in vivo and in vitro genotoxicity of aromatic amines in relationship to the genotoxicity of benzidine. Mutat Res. 1992;268(2):255-64.

19. Temcharoen P, Nerapattanakid S, Toskulkao C, Glinsukon T, Paovaro C, Ruchirawat S. Mutagenic Activity of Newly Synthesized Sulfa Drugs to Salmonella-Typhimurium. Mutat Res. 1994;321(4):187-95.

20. Arashidani K, Iwamoto-Tanaka N, Muraoka M, Kasai H. Genotoxicity of ribo- and deoxyribonucleosides of 8-hydroxyguanine, 5-hydroxycytosine, and 2-hydroxyadenine:: induction of SCE in human lymphocytes and mutagenicity in TA 100. Mutat Res-Fund Mol M. 1998;403(1-2):223-7.

21. Mullin CA, Rashid KA, Mumma RO. Mutagenic Potency of Some Conjugated Nitroaromatic Compounds and Its Relationship to Structure. Mutat Res. 1987;188(4):267-74.

22. Cheung YL, Gray TJB, Ioannides C. Mutagenicity of Chrysene, Its Methyl and Benzo Derivatives, and Their Interactions with Cytochromes-P-450 and the Ah-Receptor - Relevance to Their Carcinogenic Potency. Toxicology. 1993;81(1):69-86.

23. Zeiger E, Anderson B, Haworth S, Lawlor T, Mortelmans K. Salmonella mutagenicity tests: IV. Results from the testing of 300 chemicals. Environ Mol Mutagen. 1988;11 Suppl 12:1-157.

24. Watanabe T, Hanasaki Y, Hirayama T, Fukui S. Mutagenicity of Nitro-Substituted and Amino-Substituted Phenazines in Salmonella-Typhimurium. Mutat Res. 1989;225(3):75-82.

25. Rinkus SJ, Legator MS. Chemical Characterization of 465 Known or Suspected Carcinogens and Their Correlation with Mutagenic Activity in the Salmonella-Typhimurium System. Cancer Res. 1979;39(9):3289-318.

26. Uno Y, Matsushita H, Uehiro T, Yasuhara A, Morita M. Mutagenicity of 3-Nitrodibenzofuran and 3-Aminodibenzofuran. Toxicol Lett. 1991;55(1):31-7.

27. Sayama M, Mori M, Shirokawa T, Inoue M, Miyahara T, Kozuka H. Mutagenicity of 2,6-dinitrotoluene and its metabolites, and their related compounds in Salmonella typhimurium. Mutat Res. 1989;226(3):181-4.

28. Mori M, Miyahara T, Moto-o K, Fukukawa M, Kozuka H, Miyagoshi M, et al. Mutagenicity of urinary metabolites of 2,4-dinitrotoluene to Salmonella typhimurium. Chem Pharm Bull (Tokyo). 1985;33(10):4556-63.

29. Andre V, Boissart C, Lechevrel M, Gauduchon P, Letalaer JY, Lancelot JC, et al. Mutagenicity of Nitrosubstituted and Amino-Substituted Carbazoles in Salmonella-Typhimurium .1. Monosubstituted Derivatives of 9h-Carbazole. Mutat Res. 1993;299(1):63-73.

30. Tomosaka H, Anzai K, Hasegawa E, Horigome T, Omata S. Frameshift mutagenicity and DNA intercalation of 9-amino-2-hydroxyacridine, a rat liver S9 metabolite of 9-aminoacridine. Biosci Biotechnol Biochem. 1996;60(4):714-6.

31. You Z, Brezzell MD, Das SK, Hooberman BH, Sinsheimer JE. Substituent Effects on the in-Vitro and in-Vivo Genotoxicity of 4-Aminobiphenyl and 4-Aminostilbene Derivatives. Mutat Res. 1994;320(1-2):45-58.

32. Corbett MD, Wei CI, Johnston JJ, Chang PF, Corbett BR. Mutagenicity of the C-Nitroso Analog of Fenitrothion. Toxicol Lett. 1987;35(2-3):201-7.

33. Tanaka Y, Iwasaki H, Kitamori S. Biodegradation of herbicide chlornitrofen (CNP) and mutagenicity of its degradation products. Water Sci Technol. 1996;34(7-8):15-20.

34. Tomosaka H, Omata S, Hasegawa E, Anzai K. The effects of substituents introduced into 9-aminoacridine on frameshift mutagenicity and DNA binding affinity. Biosci Biotech Bioch. 1997;61(7):1121-5.

35. Matsushita T, Sakuma S, Nakamuro K, Matsui Y. The variation on the mutagenicity of CNP during anaerobic biodegradation. Water Res. 2001;35(11):2589-94.

36. Vikse R, Hatch FT, Winter NW, Knize MG, Grivas S, Felton JS. Structure-mutagenicity relationships of four amino-imidazonaphthyridines and imidazoquinolines. Environ Mol Mutagen. 1995;26(1):79-85.

37. Aust AE, Wold SA. Induction of Bacterial Mutations by Aminopyrazoles, Compounds Which Cause Mammary-Cancer in Rats. Carcinogenesis. 1986;7(12):2019-23.

38. Balbi A, Muscettola G, Staiano N, Martire G, De Lorenzo F. Psychotropic drugs: evaluation of mutagenic effect. Pharmacol Res Commun. 1980;12(5):423-31.

39. Kier LD, Brusick DJ, Auletta AE, Von Halle ES, Brown MM, Simmon VF, et al. The Salmonella typhimurium/mammalian microsomal assay. A report of the U.S. Environmental Protection Agency Gene-Tox Program. Mutat Res. 1986;168(2):69-240.

40. Zani F, Bellotti A, Mazza P. Biological studies on 2,1-benzisothiazole derivatives. II. Evaluation of antimicrobial and genotoxic properties of bz-nitro-, 3-ethylacetate-, 3-amino- and 3-substituted amino 2,1-benzisothiazoles. Farmaco. 1994;40(11):713-9.

41. Mor M, Zani F, Mazza P, Silva C, Bordi F, Morini G, et al. Biological studies on 1,2-benzisothiazole derivatives .5. Antimicrobial properties of N-alkanoic, N-arylalkanoic and N-aryloxyalkanoic derivatives of 1,2-benzisothiazolin-3-one: QSAR study and genotoxicity evaluation. Farmaco. 1996;51(7):493-501.

42. Glende C, Schmitt H, Erdinger L, Engelhardt G, Boche G. Transformation of mutagenic aromatic amines into non-mutagenic species by alkyl substituents. Part I. Alkylation ortho to the amino function. Mutat Res. 2001;498(1-2):19-37.

43. Iwamoto Y, Ferguson LR, Pearson A, Baguley BC. Photo-enhancement of the mutagenicity of 9-anilinoacridine derivatives related to the antitumour agent amsacrine. Mutat Res. 1992;268(1):35-41.

44. Zani F, Bellotti A, Mazza P. Biological Studies on 2,1-Benzisothiazole Derivatives .2. Evaluation of Antimicrobial and Genotoxic Properties of Bz-Nitro-, 3-Ethylacetate-, 3-Amino- and 3-Substitutedamino 2,1-Benzisothiazoles. Farmaco. 1994;49(11):713-9.

45. Andre V, Boissart C, Sichel F, Gauduchon P, LeTalaer JY, Lancelot JC, et al. Mutagenicity of nitro- and amino-substituted carbazoles in Salmonella typhimurium .3. Methylated derivatives of 9H-carbazole. Mutat Res-Gen Tox En. 1997;389(2-3):247-60.

46. Decloitre F, Hamon G, Martin M, Thybaud-Lambay V. Mutagenic activation of 3-amino-1,4-dimethyl-5H-pyrido(4,3-b)indole(Trp-P-1) and 3-amino-1-methyl-5H-pyrido(4,3-b)indole (Trp-P-2) by primary cultures of adult rat hepatocytes: effect of Aroclor induction in vitro. Mutat Res. 1984;137(2-3):123-32.

47. Zani F, Maggiali CA, Mingiardi MR, Mazza P. Biological Studies on 1,2-Benzisothiazole Derivatives .4. Relationships between Chemical-Structure and Genotoxicity. Farmaco. 1991;46(5):639-46.

48. Stevens GJ, McQueen CA. Species variation in the genotoxicity of batracylin. Toxicol Appl Pharmacol. 1994;126(1):39-44.

49. Walker BA, Rogan EG, Cromwell NH. Mutagenicity of Selected Functionalized Benz(C)Acridines and a Benz(a)Phenazine in the Salmonella-Typhimurium - Microsome Assay. Anticancer Res. 1984;4(6):399-402.

50. Grivas S, Jagerstad M. Mutagenicity of Some Synthetic Quinolines and Quinoxalines Related to Iq, Meiq or Meiqx in Ames Test. Mutat Res. 1984;137(1):29-32.

51. Jagerstad M, Grivas S. The Synthesis and Mutagenicity of the 3-Ethyl Analogs of the Potent Mutagens Iq, Meiq, Meiqx and Its 3,7-Dimethyl Isomer. Mutat Res. 1985;144(3):131-6.

52. Lin JK, Wu SS, Chen JT. Mutagenicities of nitrosated carboline derivatives. Proc Natl Sci Counc Repub China B. 1986;10(4):280-6.

53. Denny WA, Turner PM, Atwell GJ, Rewcastle GW, Ferguson LR. Structure-Activity-Relationships for the Mutagenic Activity of Tricyclic Intercalating Agents in Salmonella-Typhimurium. Mutat Res. 1990;232(2):233-41.

54. Ngoy K, Saintruf G, Demeester C. Mutagenicity of Some Derivatives of Dipyrido[1,2-a-3',2'-D]Imidazoles in Salmonella-Typhimurium with Metabolic-Activation by Rat-Liver and Small-Intestine Subcellular-Fractions. Mutat Res. 1985;156(1-2):53-9.

55. Hesbert A, Bottin MC, Deceaurriz J. Mutagenicity of 4,4'-Methylene-Bis-(2-Chloroaniline) Moca and Its N-Acetyl Derivatives in S-Typhimurium. Int Arch Occ Env Hea. 1985;55(2):169-74.

56. Vikse R, Knapstad A, Klungsoyr L, Grivas S. Mutagenic Activity of the Methyl and Phenyl Derivatives of the Food Mutagen 2-Amino-3-Methylimidazo[4,5-F]Quinoxaline (Iqx) in the Ames Test. Mutat Res. 1993;298(3):207-14.

57. Chou MW, Heflich RH, Fu PP. Metabolism of 1-Nitrobenzo[a]Pyrene by Rat-Liver Microsomes to Potent Mutagenic Metabolites. Carcinogenesis. 1986;7(11):1837-44.

58. Oshiro Y, Piper CE, Garriott ML, Balwierz PS, Soelter SG, Rohrbacher E. Genetic Toxicology Studies with a Tumor Promoter. J Appl Toxicol. 1993;13(2):91-101.

59. Ludolph B, Klein M, Erdinger L, Boche G. The effects of 4′-alkyl substituents on the mutagenic activity of 4-amino- and 4-nitrostilbenes in. Mutat Res-Gen Tox En. 2001;491(1-2):195-209.

60. Chou MW, Heflich RH, Fu PP. Multiple Metabolic Pathways for the Mutagenic Activation of 3-Nitrobenzo[a]Pyrene. Carcinogenesis. 1985;6(8):1235-8.

61. Buonarati MH, Felton JS. Activation of 2-Amino-1-Methyl-6-Phenylimidazo[4,5-Beta]Pyridine (Phip) to Mutagenic Metabolites. Carcinogenesis. 1990;11(7):1133-8.

62. Carman RJ, Vantassell RL, Kingston DGI, Bashir M, Wilkins TD. Conversion of Iq, a Dietary Pyrolysis Carcinogen to a Direct-Acting Mutagen by Normal Intestinal Bacteria of Humans. Mutat Res. 1988;206(3):335-42.

63. Lopezdecerain A, Garcia E, Gullon A. Influence of the Triazine Ring on the Mutagenicity of Triazinoindoles and Some Congeners. Mutagenesis. 1992;7(1):37-9.

64. el-Bayoumy K, Delclos KB, Heflich RH, Walker R, Shiue GH, Hecht SS. Mutagenicity, metabolism and DNA adduct formation of 6-nitrochrysene in Salmonella typhimurium. Mutagenesis. 1989;4(3):235-40.

65. Hirayama T, Watanabe T, Ono M, Fukunaga Y, Fukui S. 7-Amino-2,4,6-Trimethylquinoline as a Mutagenic Pyrolysate Compound of Polyurethane Foam. Mutat Res. 1989;226(3):169-74.

66. Jaen JC, Kropko ML, Theiss JC, Wold S, Caprathe BW, Wise LD. Assessment of Mutagenic Potential in a Series of Compounds Structurally Related to 2-Amino-3-Methylimidazo [4,5-F]Quinoline (Iq). Eur J Med Chem. 1993;28(7-8):547-53.

67. Vikse R, Klungsoyr L, Grivas S. Mutagenic activity of three synthetic isomers of the food carcinogen 2-amino-3-methylimidazo[4,5-f]quinoline (IQ) in the Ames test. Mutat Res. 1993;319(4):273-8.

68. Wakabayashi K, Kim IS, Kurosaka R, Yamaizumi Z, Ushiyama H, Takahashi M, et al. Identification of new mutagenic heterocyclic amines and quantification of known heterocyclic amines. Princess Takamatsu Symp. 1995;23:39-49.

69. Saeki K, Murakami R, Kohara A, Shimizu N, Kawai H, Kawazoe Y, et al. Substituent effect of a fluorine atom on the mutagenicity of nitroquinolines. Mutat Res. 1999;441(2):205-13.

70. Achiwa I, Shiozawa T, Nukaya H, Terao Y. Synthesis and Mutagenicity of a New Mutagen, 2-Amino-1,7,9-Trimethylimidazo-[4,5-G]Quinoxaline, and Its Analog. Chem Pharm Bull. 1994;42(2):408-9.

71. Terao Y, Achiwa I, Kishino S, Matsumura Y, Shiozawa T, Matsushita H. Mutagenic activity of 6-aminoquinoxalines in Salmonella typhimurium. Mutat Res. 1995;346(2):99-105.
